# Elucidating Pro-Inflammatory Cytokine Responses after Traumatic Brain Injury in a Human Stem Cell Model

**DOI:** 10.1089/neu.2017.5155

**Published:** 2018-01-15

**Authors:** Eric Peter Thelin, Claire E. Hall, Kunal Gupta, Keri L.H. Carpenter, Siddharthan Chandran, Peter J. Hutchinson, Rickie Patani, Adel Helmy

**Affiliations:** ^1^ Division of Neurosurgery, Department of Clinical Neurosciences, University of Cambridge, Cambridge, United Kingdom.; ^2^ Department of Clinical Neuroscience, Karolinska Institutet, Stockholm, Sweden.; ^3^ Department of Molecular Neuroscience, Institute of Neurology, University College London, London, United Kingdom.; ^4^ Department of Neurological Surgery, Oregon Health & Science University, Portland, Oregon.; ^5^ Wolfson Brain Imaging Centre, Department of Clinical Neurosciences, University of Cambridge, Cambridge, United Kingdom.; ^6^ Centre for Clinical Brain Sciences, University of Edinburgh, Edinburgh, United Kingdom.; ^7^ The Francis Crick Institute, London, United Kingdom.

**Keywords:** cytokine exposure, human embryonic stem cell-derived neurons, interleukin-1β, interleukin-6, *in vitro*, tumor necrosis factor

## Abstract

Cytokine mediated inflammation likely plays an important role in secondary pathology after traumatic brain injury (TBI). The aim of this study was to elucidate secondary cytokine responses in an *in vitro* enriched (>80%) human stem cell-derived neuronal model. We exposed neuronal cultures to pre-determined and clinically relevant pathophysiological levels of tumor necrosis factor-α (TNF), interleukin-6 (IL-6) and interleukin-1β (IL-1β), shown to be present in the inflammatory aftermath of TBI. Data from this reductionist human model were then compared with our *in vivo* data. Human embryonic stem cell (hESC)-derived neurons were exposed to recombinant TNF (1–10,000 pg/mL), IL-1β (1–10,000 pg/mL), and IL-6 (0.1–1000 ng/mL). After 1, 24, and 72 h, culture supernatant was sampled and analyzed using a human cytokine/chemokine 42-plex Milliplex kit on the Luminex platform. The culture secretome revealed both a dose- and/or time-dependent release of cytokines. The IL-6 and TNF exposure each resulted in significantly increased levels of >10 cytokines over time, while IL-1β increased the level of C-X-C motif chemokine 10 (CXCL10/IP10) alone. Importantly, these patterns are consistent with our *in vivo* (human) TBI data, thus validating our human stem cell-derived neuronal platform as a clinically useful reductionist model. Our data cumulatively suggest that IL-6 and TNF have direct actions, while the action of IL-1β on human neurons likely occurs indirectly through inflammatory cells. The hESC-derived neurons provide a valuable platform to model cytokine mediated inflammation and can provide important insights into the mechanisms of neuroinflammation after TBI.

## Introduction

Traumatic brain injury (TBI) is a common cause of death and disability.^1^ After initial impact, subsequent insults such as impaired cerebral perfusion and disrupted oxygen supply can cause secondary injuries and an increasingly neurotoxic environment for surviving cells.^2,3^ These secondary injuries have been shown to be mediated in part by components of the innate immune system,^4,5^ with inflammatory cytokines being specifically implicated.^6,7^ The cytokine response orchestrating subsequent neuroinflammation in human TBI seems to be determined both temporally and in a dose-dependent manner.^8,9^ Moreover, while it is possible that the inflammatory process is driven at least partly by cells crossing the impaired blood–brain barrier in TBI and spinal cord injury,^10,11^ there is evidence of local cytokine generation by glial cells, central nervous system (CNS) macrophages, and neuronal cells.^8,12,13^ In animal models, neuronal cells have been shown to produce cytokines, both spontaneously and after external stimuli.^7,14–16^ Studies of TBI using human neuron models with exposure to cytokines are scarce, however.^17^

Clinically, cytokines extracted using microdialysis catheters^18^ are secreted by the local cellular milieu.^8^ The inflammatory response in TBI is complex and difficult to study because of factors such as the heterogeneity between different CNS injuries and the limited tools available in the clinical setting.^4^ Moreover, even by using *in vitro* co-cultures of neurons and glial cells, the intricate interplay between different inflammatory reactive cells remains,^7,12,19^ thus complicating interpretation. A reductionist approach using human neuronal monoculture provides an opportunity to directly study specific components of the neuroinflammatory response after TBI, because it allows direct comparisons to be made between the actions of various cytokines in a highly controlled and reproducible system.

The aim of this study is to investigate the consequences of specific cytokines most widely studied in the TBI literature—namely, interleukin-1 beta (IL-1β), interleukin-6 (IL-6), and tumor necrosis factor alpha (TNF)^20^ in human neurons in a dose and time-dependent manner. Specifically, we focused on elucidating secondary cytokine responses to primary canonical pro-inflammatory cytokine exposure, and we validate these data against cytokine patterns seen in the clinical aftermath of TBI. We overcome the inaccessibility to human neuronal models by harnessing directed differentiation of human pluripotent stem cells, which represent a reductionist, reliable, and clinically relevant model system.

## Methods

### Human embryonic stem cell culture

The methods used to generate neuronal cells have been described previously in detail and used in previous studies.^21,22^ Briefly, two human embryonic stem cell (hESC) lines, H9 (WiCell Research Institute) and HuES9 (hES facility, Harvard University) were propagated on a layer of irradiated mouse fibroblasts. These cells were enzymatically dissociated, mechanically triturated, centrifuged, and washed and resuspended in medium consisting of 50% Iscove modified Dulbecco medium (IMDM; Gibco) plus 50% F12 plus Glutamax (Gibco), supplemented with 1.75 mM human recombinant insulin (Roche), 0.38 mM transferrin (Roche), 450 μM of monothioglycerol (Sigma), 10 μL/mL−1 lipids (Sigma), and 5 mg/mL−1 bovine serum albumin fraction V (Sigma).

To generate neural precursors, previously published methods were adapted to generate predominantly (approximately 80%) cerebral neurons.^21,22^ Detached colonies were centrifuged subsequently and washed in fresh medium. Colonies were next chopped at 150 μm intervals using a McIlwain tissue chopper (Mickle Engineering, Gomshall, U.K.) before being plated at low density in chemically defined medium, which consisted of 50% IMDM (Gibco) plus 50% F12 plus Glutamax (Gibco), 1.75 mM human recombinant insulin (Roche), 0.38 mM transferrin (Roche), 450 mM of monothioglycerol (Sigma), 10 mg/mL lipids (Gibco), and 5 mg/mL bovine serum albumin fraction V (Sigma) in 10-cm plastic culture dishes on an orbital shaker. Neural precursors (NPCs) were maintained in chemically defined medium in the presence of 20 ng/mL of FGF2 from day 8.

For terminal differentiation, hESC-NPCs were plated onto poly(d-lysine)/laminin-coated coverslips and cultured in Dulbecco modified Eagle medium (DMEM)/2% B27/1% penicillin/streptomycin/fungizone (PSF), 10 ng/mL−1 BDNF (brain-derived neurotrophic factor; R&D Systems) and 10 ng/mL−1 GDNF (glial cell-derived neurotrophic factor; R&D Systems) in the absence of mitogens. Cells were incubated at 37°C in 5% CO_2_ with replacement of the culture medium every 72 h. Cells were cultured within 24 well plastic plates on coverslips in which the volume of culture medium was kept at 1 mL per well.

### Experimental conditions

Human recombinant IL-1β, IL-6, and TNF (Peprotech EC, London, UK) was sourced as lyophilized powder, which was reconstituted according to the manufacturer's instructions. Serial dilution of this stock solution generated the various concentrations required to generate the final concentrations in Table 1 within the 1 mL wells. Each cytokine was used at four concentrations covering the range of concentrations seen within human microdialysis studies after correction for relative recovery determined *in vivo,*^8^ as well as covering higher doses that might be seen in the early phase of injury before microdialysis monitoring is instituted. Control wells with untreated cells were also sampled to provide a comparator group.

**Table 1.** Summary of Significant Effects of Added Cytokine on Measured Cytokines

	*IL-1β Added*				*IL-6 Added*				*TNF-α Added*			
*Cytokine measured*	*Conc.*	*1 h*	*24 h*	*72 h*	*Conc.*	*1 h*	*24 h*	*72 h*	*Conc.*	*1 h*	*24 h*	*72 h*
EGF								X				
Eotaxin									X			
FGF2							X					
Flt3lig												
Fractalkine							X	X				X
G-CSF												
GM-CSF							X	X	X			
GRO											X	X
IFNα2						X	X					
IFNγ						X	X	X				
IL-1α					X						X	X
IL-1β												
IL-1ra							X					
IL-2												
IL-3												
IL-4					X							
IL-5						X	X					
IL-6											X	
IL-7						X	X	X		X		X
IL-8											X	X
IL-9												
IL-10							X					X
IL-12p40						X	X	X		X		
IL-12p70						X	X	X				
IL-13							X	X				
IL-15												
IL-17												
IP-10	X								X			
MCP-1												
MCP-3											X	X
MDC												
MIP1α								X				
MIP1β							X				X	X
PDGFAA												
PDGFABBB												X
RANTES						X				X	X	X
sCD40L					X							X
sIL-2Ra						X						
TNF-α												
TNF-β						X	X	X				
VEGF												

EGF, epidermal growth factor; FGF2, fibroblast growth factor 2; Flt3lig, Fms-related tyrosine kinase 3 ligand; G-CSF, granulocyte colony-stimulating factor; GM-CSF, granulocyte-macrophage colony-stimulating factor; GRO, chemokine (C-X-C motif) ligand 1 (CXCL1); IFN, interferon; IL, interleukin; IL-1ra, interleukin-1 receptor antagonist; IP-10/IP10, interferon gamma-induced protein 10 (also known as C-X-C motif chemokine 10 (CXCL10)); MCP-1, monocyte chemotactic protein 1 (also known as CCL2); MCP-3, monocyte chemotactic protein-3 (also known as CCL7); MDC, macrophage-derived chemokine (also known as CCL22); MIP1α, macrophage inflammatory protein 1 alpha (also known as CCL3); MIP1β, macrophage inflammatory protein 1 beta (also known as CCL4); PDGF, platelet-derived growth factor; RANTES, regulated on activation, normal T cell expressed and secreted (also known as CCL5); sCD40L, soluble CD40 ligand; sIL-2Ra, soluble Interleukin-2 receptor antagonist; TNF, tumor necrosis factor; VEGF, vascular endothelial growth factor.

The columns with bold borders represent each of the experimental conditions (IL-1β, IL-6, and TNF-α). Within these headings, cytokine induction only affected by concentration difference (“Conc.”) of the added cytokine, as well as on each individual time point (1 h, 24 h, 72 h), are tested in a multivariate analysis of variance. The concentration or time dependent induction of a given cytokine is highlighted with an X if significant (*p* < 0.05). Detailed description of the analyses performed is available in Supplementary Figure 1.

The cytokine solution (10 μL) was added to 990 μL of culture within each well to make up the desired final concentration within 1 mL at the time of medium replacement. Cell cultures were then returned to an incubator. Samples were taken as detailed below. We used two different hESC lines, and 2–3 separate neural inductions per line, giving us 3–5 repeats for each experiment.

### Sample collection and storage

Samples (60 μL) were taken from each well culture supernatant at the given time points. Five baseline samples were taken before adding any cytokine. Further samples were taken at 1 h, 24 h, and 72 h after addition of the respective cytokine (*n* = 3 per time point). When performing the experiments, seeding density was 100,000 cells per well of a 24-well plate. Samples were stored at −80°C until analysis.

### Cytokine analysis

The cell culture supernatants from three wells for each experimental condition were analyzed in duplicate wells using the Milliplex^™^ MultiAnalyte Profiling (MAP) Human Cytokine/Chemokine 42 analyte pre-mixed kit (Millipore Corp, St. Louis, MO) using the manufacturer's instructions as described previously.^23^ The plates were read on a Luminex 200 analyzer (Luminex Corporation, Austin, TX) running STarStation software (Applied Cytometry Systems, Sheffield, UK). Cytokine concentrations were calculated by reference to an eight-point five-parameter logistic standard curve for each cytokine.

### Immunocytochemistry

Neurons were differentiated on poly-D-lysine/laminin coated glass coverslips. These were fixed with 4% fresh paraformaldehyde for 20 min at room temperature before being washed three times with phosphate-buffered saline (PBS). Samples were then blocked for 1 h at room temperature with 0.3% Triton/ PBS/5% goat serum before being incubated overnight with primary antibody in 0.2% Triton/PBS/2% goat serum at 4°C. After three washes in PBS, secondary antibody (goat anti-mouse, Alexa Fluor 488 or 555, 1:1000) in PBS/Hoechst (1:4000) was next applied for 1 h at room temperature. Primary antibodies used included β-III tubulin (1:500; Sigma- Aldrich), orthodenticle homeobox 1 (OTX1), REELIN, T-Box Brain 1 [TBR1] (1:50; Developmental Studies Hybridoma Bank [DSHB], Iowa City), Forkhead Box G1 (FOXG1) (1:100; Abcam), Synapsin (1:500; Calbiochem), microtubule associated protein 2ab (MAP2ab) (1:500; Sigma-Aldrich), gamma-aminobutyric acid (GABA) (1:500; Sigma-Aldrich) and glutamate (1:500; Sigma-Aldrich).

### Clinical cytokine data from patients with TBI

We extracted data from two previously published studies from our group describing the temporal profiles and concentrations of cytokines from patients with severe TBI.^8,24^ In a pilot trial of IL-1 receptor antagonist treatment for TBI, we used brain cytokine concentrations from the placebo group.^24^ Demographic data for all patients are available in Supplementary Table 1 (see online supplementary material at ftp.liebertpub.com), including age, sex, admission Glasgow Coma Scale (GCS) and head computed tomography (CT) verifiable injury as defined by Marshall CT classification (Grade II–IV = diffuse and Grade VI = focal).^25^ As described in detail in the previous studies,^8,24^ patients were monitored for approximately five consecutive days, and cytokine samples were pooled from 6-h periods. While there was some disparity in commencement of monitoring after the traumatic ictus, all data have been corrected to the time of injury to enable comparison with the *in vitro* experiments.

### Statistical analysis

The effect of each of the added cytokines (IL-1β, IL-6, TNF) on the measured cytokines (42 cytokine panel) was analyzed using a two-way mixed analysis of variance (ANOVA) (to compare concentrations of each cytokine analyzed). The time at which the sample was taken (“Time”; 0 (baseline levels), 1 h, 24 h, 72 h) was used as the repeated measure (within subject) variable. The concentration of added cytokine (“Concentration”; untreated, and four concentrations of added cytokine in Table 1) was used as the independent (between subject) variable. No assumptions were made about sphericity of the data, and the more conservative Greenhouse-Geisser method was used to determine the F-ratio significance value for the effect of Time and the interaction of time and concentration (“Time*Concentration”). The conventional ANOVA F-ratio significance value was used for the independent variable “Concentration.” For those cytokines in which the ANOVA significance value was <0.05, *post hoc* tests were used to explore the nature of this contrast.

Tests of contrast within “Time” and the interaction of “Time*Concentration” were tested against three potential models: linear, quadratic, and cubic. The model with the most stringent *p* value is quoted. In cases where the *p* values are identical, the relevant models are listed. In the case of Concentration, the Bonferroni multiple comparison test was used to determine significance values between each of the added concentrations.

In a separate analysis, we have compared the effects of the three added cytokines in a multivariate ANOVA in which the dependent variables were the measured cytokines at three time points (i.e., cytokine measured at 1 h, cytokine measured at 24 h, and cytokine measured at 72 h) and the independent variable was the cytokine added (No cytokine added, IL-1β, IL-6, TNF). In this case, the data from the control wells with untreated are not incorporated into the series of concentrations but are compared as a separate group: “untreated.” For the F-ratios and *post hoc* tests described above, a *p* value of ≤0.05 has been used throughout.

Descriptively, we compared the temporal profiles of cytokine release as well as concentrations *in vivo* from a previous study and compared them with our results.^8,24^

## Results

### hESC-derived neuronal cultures

**FIG. 1.**
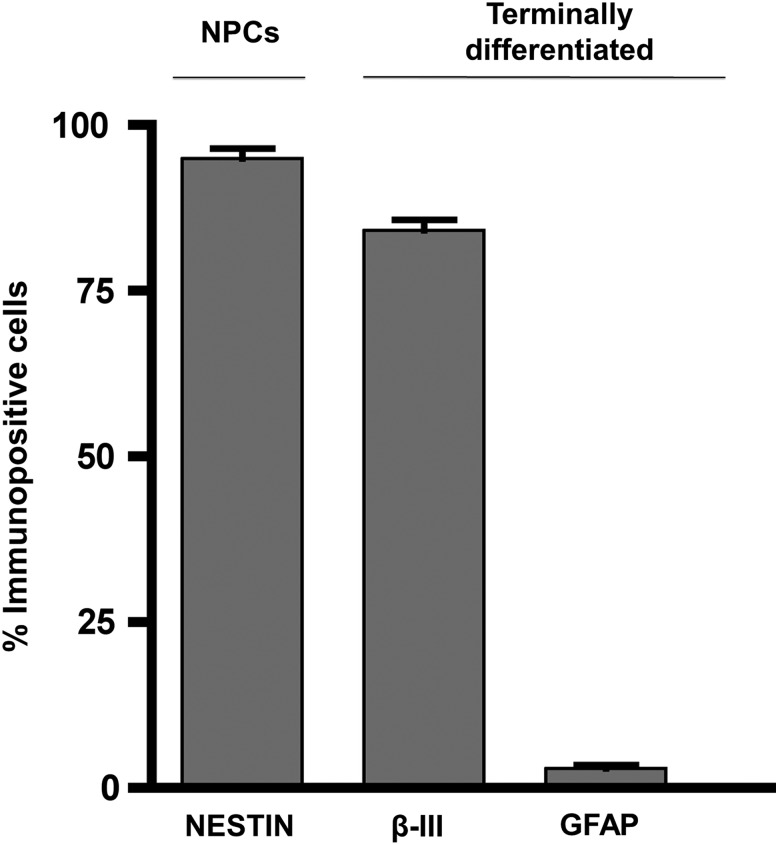
Percentage immunopositive cells after neural induction for NESTIN (95.0 ± 1.5%) and on terminal differentiation for βIII-tubulin (84.1 ± 1.6%) and glial fibrillary acidic protein (GFAP) (2.9 ± 0.6%). Bars represent mean ± standard error of the mean. NPCs, neural precursors.

**FIG. 2.**
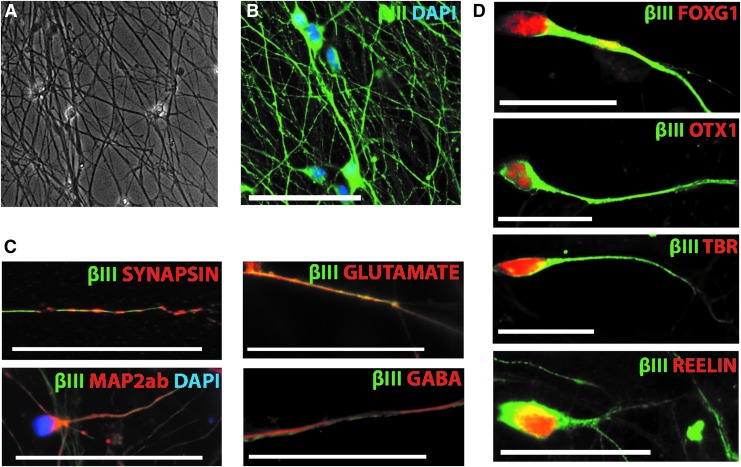
**(A**) Phase contrast image ( × 200) showing the enriched neuronal cultures that were used in the experiments. (**B**) Illustrates immunocytochemistry of the neuronal culture with βIII-tubulin stained neurons (green) and DAPI (blue) as cellular counterstaining (scale bar: 50 μm). (**C**) Confirms neuronal and neurotransmitter markers by staining for synapsin, glutamate, MAP2ab, and GABA (red), DAPI (blue), and βIII-tubulin (green) (scale bar: 50 μm). (**D**) Illustrates cortical markers, resulting as a default regional identity after neural induction by staining for FOXG1, OTX1, TBR1, and REELIN (scale bar: 25μm). βIII-tubulin, Beta-III-tubulin; DAPI, 4',6-diamidino-2-phenylindole; MAP2ab, microtubule associated protein 2ab; GABA, gamma-aminobutyric acid; FOXG1, Forkhead Box G1; OTX1, orthodenticle homeobox 1; TBR, T-Box, Brain 1.

After neural induction, we detected 95.0 ± 1.5% NESTIN positive cells (Fig. 1), suggesting efficient neural conversion consistent with previous studies.^21,22^ This was reinforced by a high enrichment of neurons on terminal differentiation; 84.1 ± 1.6% of total cultures were βIII-tubulin positive and exhibited characteristic neuronal morphology (Fig. 2A,B). Notably only 2.9 ± 0.6% of total cells were glial fibrillary acidic protein (GFAP) positive (Fig. 1), confirming a minimal presence of astrocytes. Less than 1% of the resulting cultures were O4 positive, suggesting negligible oligodendrogenesis (data not shown), which when considered together with approximately 3% astrocyte specification is consistent with the predominantly neuronal (rather than glial) potential of neural precursors soon after neural induction.

The terminally differentiated neuronal cultures also expressed synapsin, glutamate, GABA and MAP2ab (Fig. 2C). In line with the expected/default telencephalic neuronal fate, we also confirmed the expression of cortical neuronal markers including TBR1, OTX1, REELIN, and FOXG1 (Fig. 2D). This enriched neuronal population containing cortical derivatives serves as a reductionist human *in vitro* model for further study.

### Induction of cytokine response

**FIG. 3.**
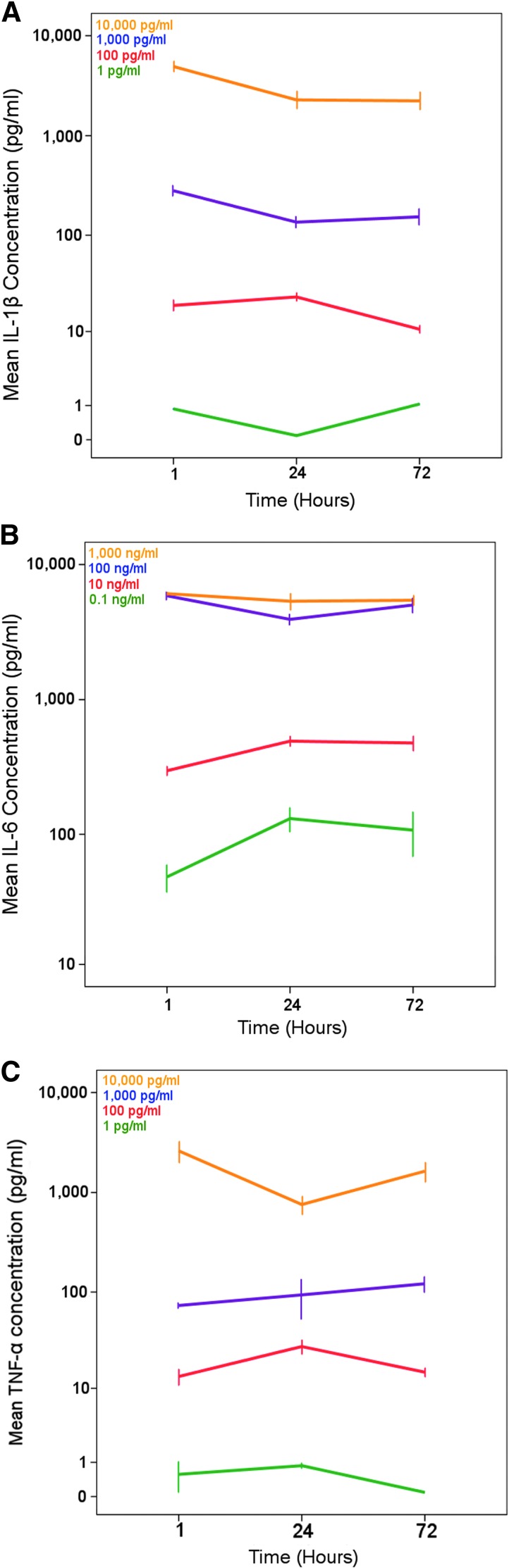
Illustrating the different concentrations of cytokines (y-axis log, error bar illustrates mean ± standard error of the mean) that were used for (**A**) IL-1β, (**B**) IL-6, and (**C**) TNF over time (1, 24, and 72 h x-axis). As can be seen in the Panel legends, the different colors of the lines are escalating concentrations of the added cytokine. The same colors/concentrations also are present in Supplementary Figure 1. IL, interleukin; TNF, tumor necrosis factor.

Cytokine generation from the enriched neuronal cultures was induced by exogenously adding escalating concentrations of IL-1β, IL-6, and TNF analogous to the aftermath of TBI (Fig. 3A–C). The concentrations of exogenously added cytokines are maintained across all time points during the experimental period (Fig. 3). All cytokines assayed were detected at some point within our experimental paradigm, other than transforming growth factor beta (TGFβ).

### Temporal and concentration dependent effect of cytokine-stimulated neuronal cultures

IL-6 and TNF induction resulted in a concentration and time dependent increase of 15 and 11 cytokines, respectively, while IL-1β only showed a concentration dependent induction for IP10 (Table 1). Moreover, cytokine responses to IL-6 and TNF induction were in general different, only overlapping for IL-7. Table 1 summarizes the results from the relevant *post hoc* tests regarding the effects of adding each of the three cytokines on measured cytokine at different concentrations and at each of the time points. Supplementary Table 2 summarizes the results of the relevant *post hoc* tests from the two-way mixed ANOVA performed for each cytokine (Supplementary Table 2 and Supplementary Fig. 1; see online supplementary material at ftp.liebertpub.com).

### Patterns of cytokine release

**FIG. 4.**
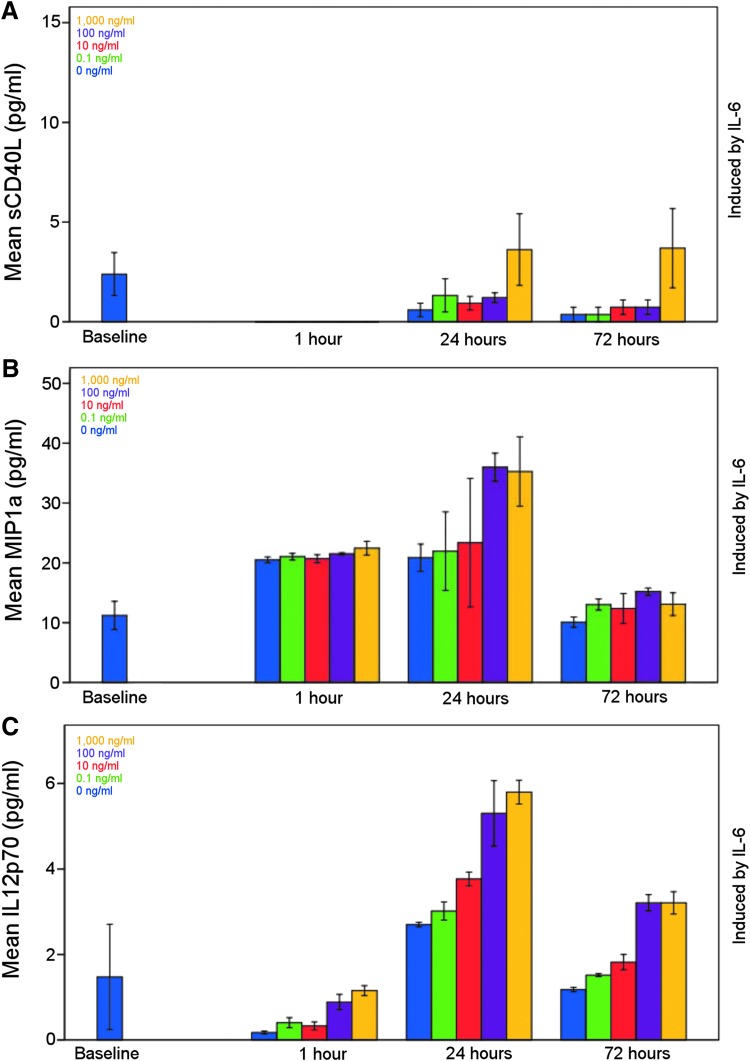
Mean sCD40L (**A**), MIP1a (**B**), and IL-12p70 (**C**) concentrations as induced by IL-6 to exemplify the different release patterns of a concentration dependent, a time dependent, and a time and concentration dependent release of cytokines (as seen in Table 3). Bars representing escalating concentration as seen in the legend included and is mean ± standard error of the mean. sCD40L, soluble CD40 ligand; MIP1α = macrophage inflammatory protein 1 alpha; IL, interleukin.

Some cytokines are produced in a concentration dependent fashion (Fig. 4A, pattern 1). Conversely, some are produced at a given time in response to the added cytokine but do not show a statistically significant effect in response to increasing concentrations of primary cytokine exposure (Fig. 4B, pattern 2). A further pattern was observed where cytokines show both a time and a concentration dependency (Fig. 4C, pattern 3). Table 2 summarizes these three patterns of cytokine production after cytokine exposure, which are exemplified for a subset of cytokines produced in response to IL-6 (Fig. 4). Similar principles, however, are applicable across a wider range of cytokines (Table 2 and Supplementary Fig. 1).

**Table 2.** Summary of Cytokine Induction

	*Cytokine induced*		
*Cytokine added*	*Pattern 1 (concentration dependent)*	*Pattern 2 (time dependent)*	*Pattern 3 (time and concentration dependent)*
IL-1β	IP10		
IL-6	IL-1α, IL-4, sCD40L	IL-1ra, IL-5, IL-13, MIP1α, RANTES	EGF, FGF-2, Fractalkine, GM-CSF, IFNα2, IFNγ, IL-7, IL-12p40, IL-12p70, MIP1β, sIL-2Ra, TNFβ
TNF	Eotaxin, GM-CSF, IP10	Fractalkine, IL-6, IL-10, PDGF ABBB	GRO, IL-1α, IL-7, IL-8, MCP3, MIP1β, RANTES, sCD40L

IP-10, interferon gamma-induced protein 10; sCD40L, soluble CD40 ligand; MIP1α, macrophage inflammatory protein 1 alpha; RANTES, regulated on activation, normal T cell expressed and secreted; EGF, epidermal growth factor; FGF2, fibroblast growth factor 2; GM-CSF, granulocyte-macrophage colony-stimulating factor; IFN, interferon; MIP1β, macrophage inflammatory protein 1 beta; sIL-2Ra, soluble interleukin-2 receptor antagonist; PDGF, platelet-derived growth factor; MCP-3, monocyte chemotactic protein-3.

The three patterns of cytokine induction detected after interleukin (IL)-1β, IL-6, and tumor necrosis factor (TNF) exposure. Pattern 1: concentration dependent but time independent cytokine response; Pattern 2: time dependent but concentration independent cytokine response; Pattern 3: time and concentration dependent cytokine response.

### Cytokine temporal patterns *in vivo* vs. *in vitro*

In previous studies, we have characterized a time-resolved cytokine response after TBI.^8,24,26^ We compared the time dependent increase in the present study with our previous data (Table 3).^8^ Several cytokines, including IL1ra, IL6, IL7, IL8, GRO, MIP1β, and RANTES exhibit similar levels *in vivo* as they do in our *in vitro* model. The concentrations of cytokines were consistently higher in the *in vivo* study,^24^ with some selected cytokines exhibiting a strikingly higher concentration *in vivo* than others (factor >100 × ) (i.e., G-CSF). Other cytokines had a more modest increase (e.g., RANTES factor 5-10x; Table 3).

**Table 3.** Cytokine Temporal Pattern in vivo vs. in vitro

*Cytokine*	*Time to peak* in vivo	*Time to peak* in vitro	*Mean concentration* in vivo *(ECF) (pg/ml)*	*Mean concentration* in vitro *(supernatant) (pg/ml)*	*Factor higher* in vivo
TNF	<24h	–	19.0	1.5	12.7
IL7	Up to 24 h	1–72 h	46.7	26.1	1.8
IL8	Up to 24 h	24–72 h	5871	156	37.6
MIP1α	Up to 36 h	72h	531	25.6	20.7
sCD40L	Up to 48 h	72h	234	1.9	123
GRO	Up to 48 h	24–72 h	1299	67.5	19.2
IL1β	Up to 48 h	–	16.5	1.4	11
PDGF AA	Up to 48 h	–	3690	418	8.8
MIP1β	Up to 60 h	24–72 h	680	27.2	25
RANTES	Up to 60 h	1–72 h	213	40.0	5.3
IL1ra	24–72 h	24h	208	10.2	20.4
IL6	24–72 h	24h	5789	289	20
G-CSF	24–72 h	–	3650	5.2	702
IP10	24–72 h	–	47345	709	66.8
IL12p70	96–144 h	1–72	29.7	3.5	8.5
IL10	96–144 h	24–72 h	73.0	9.1	8.0

TNF, tumor necrosis factor; IL, interleukin; MCP-1, monocyte chemotactic protein 1; sCD40L, soluble CD40 ligand; GRO, chemokine (C-X-C motif) ligand 1; PDGF, platelet-derived growth factor; RANTES, regulated on activation, normal T cell expressed and secreted; G-CSF, granulocyte colony-stimulating factor; IP-10/IP10, interferon gamma-induced protein 10.

Table depicts the temporal release patterns of some cytokines previously analyzed in the aftermath of human TBI,^1^ and concentrations seen in the extracellular fluid after TBI.^2^ Time to peak describes when the highest concentrations could be seen after trauma or cytokine induction. The concentration is compared between the brain extracellular fluid (ECF) as seen after TBI and in the culture supernatant. The factor higher concentration *in vivo* compared with *in vitro* is noted.

1. Helmy, A., Carpenter, K.L., Menon, D.K., Pickard, J.D., and Hutchinson, P.J. (2011). The cytokine response to human traumatic brain injury: temporal profiles and evidence for cerebral parenchymal production. J. Cereb. Blood Flow Metab. 31, 658–670.

2. Helmy, A., Guilfoyle, M.R., Carpenter, K.L., Pickard, J.D., Menon, D.K., and Hutchinson, P.J. (2014). Recombinant human interleukin–1 receptor antagonist in severe traumatic brain injury: a phase II randomized control trial. J. Cereb. Blood Flow Metab. 34, 845–851.

## Discussion

We have established a reproducible platform to measure the secondary cytokine responses in human enriched cortical neurons subjected to primary canonical pro-inflammatory cytokines. Importantly, we based our target concentration ranges on previously determined human TBI conditions. We demonstrated that a wide range of inflammatory mediators is differentially induced by the addition of IL-6 and TNF and that the response was both time- and/or concentration-dependent. Conversely, IL-1β only induced IP10, which likely reflects the absence of glia in our neuronal culture paradigm. Compared with the clinical conditions in brain extracellular fluid (ECF), however, our cytokine concentrations were lower, which is again likely attributable to the contribution of glial cells to the neuroinflammatory response, and argues for future studies focusing on astrocytes and microglia.

The principal CNS immune mediators are long-held to be microglial cells,^27^ while more recently a potent role of astrocytes has been shown,^28–30^ with an intricate interplay between these cell types.^31,32^ Studies on neurons as immunocompetent cells are scarce, but it has been shown that they can produce major histocompatibility complex (MHC) 1 mRNA in response to inflammatory (interferon [IFN]γ) stimulation,^33^ which results in diverse immunological responses to different stimuli.^34,35^ Moreover, it has been suggested that the immunological actions of neurons could cause ongoing neuroinflammation in neurodegenerative diseases,^36,37^ which could in theory be initiated, or augmented, by TBI. Importantly, while an increase of commonly considered pro-inflammatory cytokines such as IL-1β and IL-6 have been associated with an unfavorable outcome for patients with TBI,^38,39^ we believe it would be overly simplistic to draw conclusions from individual cytokine levels because of the complex and dynamic cellular interplay that orchestrates the inflammatory response. Indeed, these processes have been shown to exert both harmful and beneficial capabilities after brain injury.^40,41^

In this study, we have deliberately chosen not to separate downstream assayed cytokines into pro- and anti-inflammatory groups. Moreover, to give cytokines a distinctively pro- and anti-inflammatory role might not be accurate,^42^ and from our experiences using anti-inflammatory treatment (IL-1 receptor antagonist in TBI), the response is complex, and different pathways and clusters may form making it difficult to determine specific inflammatory preference.^26^ Thus, we looked at patterns incorporating several cytokines, a similar approach as we recommend in the clinical situation.^43,44^ In summary, we observed production of different patterns of cytokines in enriched human cortical neuronal cultures after cytokine induction, which may alter pathophysiological processes in both potentially beneficial and unfavorable ways in the *in vivo* situation.

While it has been shown that cultured cells of the CNS produce cytokines,^12,45,46^ this has been in a disparate range of cultures. Previously, cultured rat sympathetic ganglia and rat dorsal root ganglia have been shown to produce cytokines (IL-1β and IL-6),^13,15^ mouse embryonal neurons have been induced with cytokines where IL-6 and chemokines IP10/CXCL10, MCP-1/CCL2, and KC/CXCL1 concentrations were assessed,^16^ and rat pyramidal neurons have shown IL-6 mRNA production.^14^ To our knowledge, however, this is the first study to utilize enriched human neuronal cultures, specifically with a cortical phenotype, in this context.

This study provides a translational, complementary *in vitro* system to parallel our ongoing clinical research in human subjects.^8^ Moreover, because of logistical reasons, it is difficult to measure the inflammatory response the first hours after trauma in patients with severe TBI.^8^ Based on the animal literature, the initial hours after trauma usually present the highest levels of key cytokines,^47–49^ and given that anti-inflammatory treatments in TBI are highly sought after,^50^ the current *in vitro* platform holds promise for future translational work. Further, one of the main benefits of a hESC system is that it may be used to study further applied clinical scenarios, such as cytokine inhibitors,^51^ on functional consequences and cytokine expression patterns. In aggregate, while nonhuman neuronal cultures have been shown to generate cytokines, we present an *in vitro* model of enriched human cortical neuronal cultures that may be used to investigate inflammatory mediators, serving as a valuable pre-clinical model for putative TBI therapies.

While both IL-6 and TNF exposure induce a wide range of inflammatory cytokines, chemokines, and growth factors, IL-1β did not induce any downstream cytokines in our neuronal cultures other than IP10. An increase in IP10 has been shown previously by Tsakiri and colleagues,^16^ using mouse embryonic neuronal cultures exposed to IL-1. They reported an increased production of several additional other cytokines, however, which could be attributed to the higher concentrations of IL-1 used (mg/mL instead of ng/mL and pg/mL in our study), thus not representative of clinically relevant levels.^8,16^ Using embryonic murine cortical neurons *in vitro,* Ringheim and colleagues^52^ noted that IL-1 and TNF stimulation led to IL-6 and IL-6 mRNA production in the cells. Of the two, TNF stimulation led to greater increase, but similar to the study by Tsakiri and colleagues,^16^ they also used higher, nonphysiological concentrations of IL-1 and TNF.

Our finding that IL-1β induced a limited cytokine response may reflect a relative paucity of IL-1 receptors (IL-1R) on the neuronal population. We analyzed RNA sequencing data from equivalently enriched neuronal and astrocyte cultures derived from human pluripotent cells^53^ to investigate IL-1R 1 (IL1R1) and IL-1 receptor accessory protein (IL1RAP) expression. The IL1RAP plays an essential role for IL1R1 signaling in the brain,^54^ making them essential to assess together. We found that IL1R1 and IL1RAP were similarly expressed in neurons, but exhibited lower expression than in astrocyte cultures (Supplementary Fig. 2; see online supplementary material at ftp.liebertpub.com).

Further, it has been shown that IL-1β is not directly toxic in neuronal cell culture in the absence of glia,^51,55^ suggesting that neurons may not be the primary site of action of IL-1β in this context, consistent with higher receptor mRNA levels in enriched astrocyte cultures. Previously, neurons have been shown to produce higher levels of IL-1β with other glial cells, such as astrocytes and microglia,^56^ where IL-1β has been shown to demonstrate a potent dose-dependent neurotoxic effect.^51,57^ Alternatively, using individual IL-1β treated astrocytic culture supernatant on a human fetal neuron culture also induced neuronal death.^55^ In a further human fetal neuronal/glial co-culture model, IL-1β and TNF did not show neurotoxic effects in isolation but caused neuronal death in combination.^58^ In a similar model system, IL-1β and IFNγ also demonstrated a synergistic neurotoxic effect, possibly via induction of TNF.^19^ In contrast, IL-6 has been shown more commonly to have a neurotrophic action; however, like many cytokines, the exact actions are dependent on culture model, duration, and concentration of exposure.^59,60^ In aggregate, IL-1β exposure did not lead to the same extent of cytokine production as IL-6 and TNF, which could be because of the enriched neuronal monoculture setup.

Cytokines that increased over time after exposure to different IL-6 and TNF concentrations differed greatly, only really overlapping in IL-7 levels. This suggests that there are distinct signature cytokine release profiles in response to specific exogenous cytokine triggers. For example, IL-6, TNF, and IL-1β, while they are usually grouped together as “pro-inflammatory cytokines,” exert vastly different effects in our paradigm.^61,62^ IL-1β and TNF signaling work primarily through nuclear factor-κβ (NFκβ) translocation to the nucleus where an array of inflammatory genes is activated in response.^63,64^ While IL-6 has a comparable JAK-STAT and MAPK nuclear translocation system, it can operate through either a transmembrane receptor or a soluble receptor, which greatly increases its capacity.^65^ Similarly, TNF also has a soluble receptor that can interact directly with cells,^66^ but which is not evident to the same extent for IL-1β. Thus, the internal signaling and amplification through soluble receptors might, in part, explain the differences in secondary cytokine induction (and indeed level of induction), after exposure to a defined primary cytokine stimulus.

Ensuing from our data analysis, three patterns of cytokine induction are apparent where the first pattern is concentration dependent, the second is time dependent but not concentration dependent, and the third is both time dependent and concentration dependent (Fig. 4 and Table 2). One would expect that if a cytokine is induced, it will show dependency on the concentration of the added cytokine if within the physiological range of stimulation, and differences in concentration of the induced cytokine will be apparent when the results for different added cytokines and observation time-points are compared.

Previous studies induced a cytokine response by adding IL-1β and TNF to embryonic CNS cell mixed cultures^12,57^ and noticed a dose- and time-dependent production of IL-6, IL-1β, and TNF produced mainly by microglia. In comparison with our previous *in vivo* work,^8,24,26,43^ while the sampling resolution was different, many of the cytokines after TBI, (e.g., RANTES, IL-7, IL-8, GRO, and MIP1β) seem to follow similar temporal trends *in vitro* as in patients (Table 3).^8^ Hence, this *in vitro* model supports the time-dependent endogenous production of cytokines.^8^ Moreover, all cytokine concentrations measured were lower than in the *in vivo* situation (Table 3), but for many, the ratio between *in vivo* and *in vitro* was much higher suggesting that production of these cytokines is predominantly driven by glial cells compared with others.^56^ Cytokines with a lower ratio between *in vivo* and *in vitro*, such as IL-8, could also suggest that neuronal production could contribute in part. Microdialysis estimation of *in vivo* concentrations also suffers from low relative recovery (proportion extracted of the true extracellular concentration) of cytokines in comparison with the *in vitro* situation where the “true” concentration can be directly analyzed.^8,23^

Future studies, using co-cultured cells, can demonstrate the different potency in cytokine production between glial and neuronal cells. In summary, our study harnesses a human neuronal model to gain clinically relevant insight into the temporal consequences of exposure to key cytokines implicated in TBI.

### Limitations

While IL-6 has been shown to induce the largest number of cytokines in this model, this may stem from its use at several-fold higher concentration than TNF and IL-1β. Moreover, when confirming the administered levels of IL-6 in the cultures (Fig. 3), the maximum concentration added (1,000 ng/mL) exceeded the concentration range of the assay (∼100 ng/mL), and therefore the apparent result generated by the assay returns this upper limit value at all time points. This increased concentration range was chosen, however, based on human studies described previously, as to best model the clinical conditions.^8^

Any *in vitro* model will not be representative entirely of the *in vivo* situation, particularly in relation to interactions between different cell types. In addition, the neuronal phenotype might differ from what is seen in humans *in vivo* in a single cell type enrichment culture. This is important particularly in the context of the inflammatory response to trauma because microglia may be an important driver of the balance between neurodegeneration and neuroprotection.^67,68^ Nevertheless, what is lost in terms of the accuracy from the model is gained in the interpretation of the results in a highly defined and human system.

The choice of cytokines to measure is somewhat arbitrary; however, they were chosen based on a wide screen of potential mediators using multiplex technology. Importantly, they allow for comparison with our clinical studies where the neuroinflammatory response after TBI is assessed using down-stream cytokine responses in human subjects with TBI.^8,24,26^

The purity of the cultures used has been described previously in detail.^21^ This is a key determinant of the interpretations drawn from these data. Although we are confident of the high purity of the neuronal culture used (approximately 84%, Fig. 1) and that the cytokines thus have a neuronal origin, a minority of alternative cell types, mainly neuronal precursors (approximately 13%) but also some astrocytes (approximately 3%), could contribute to total cytokine production. The lack of any substantial cytokine production on addition of IL-1β, however, suggests that astrocytes were likely not present to any great extent in the cultures.^69^ Moreover, microglia are effectively excluded from our cultures because of different developmental origins and thus divergent culture protocols necessary to specify these cells. It would be possible to refine cultures further using fluorescence-activated cell sorting (FACS) techniques and appropriate antibodies. This would add physical stress, however, including shearing forces, osmotic stress, and laser damage,^70^ which would alter the cells and presumably induce or modify a cytokine response. For future experiments, we will aim to establish the exact origin of cytokines in terms of cell-type as well as receptor expression of the affected cells.

The TGFβ was the only cytokine that could not be measured at any time point in the culture medium, while several other cytokines appeared at low concentrations approaching the limit of sensitivity of the Luminex assay. For example, at the 1-h time point, FGF2, Flt3lig, GCSF, IL-2, IL-5, IL-15, and IL-17 appear to have very consistent concentrations across all wells assayed. This is likely to reflect the insensitivity of the assay at the lower limits of the standard concentration curve, such that the fluorescence intensity is interpreted as the lowest quantifiable standard. In this case, the results are not interpretable. Because each time point was run on a separate plate, the lower quantifiable limit may vary from time point to time point.

We did not analyze cellular fate in the neuronal cultures after the cytokine exposure. While this was not within the primary scope of the study, it would have provided information about the state of the cells or the rate of cellular death. We used relatively low concentrations of cytokines, however, compared with similar studies that show cellular toxicity.^16^ For instance, similar concentrations of IL-1β do not affect cellular viability above vehicle controls.^51^ For IL-6 and TNF, similar concentrations have even been shown to be neuroprotective for retinal ganglion cells and enteric neurons, respectively.^71,72^ Thus, while it is not possible to exclude the possibility that some neurons might have succumbed because of cytokine exposure, the available literature suggests that this is probably not the case, because of the relatively low concentrations used in the present study.

## Conclusions

This study has demonstrated a reproducible platform for examining a range of inflammatory mediators for their putative effects on enriched human neuronal cultures, providing a useful comparator for data after human TBI. We found that a range of cytokines is induced by the addition of IL-6 and TNF in a dose- and time-dependent manner. Additional research is necessary to fully explore the pathophysiological response after cytokine induction *in vivo* and *in vitro*. Importantly, the patterns of cytokine response we uncovered here are consistent with our *in vivo* experiments in human subjects with TBI, thus validating our human stem cell-derived neuronal platform as a clinically useful reductionist model.

## Supplementary Material

Supplemental data
