# Supplementary material for: Elucidating Pro-Inflammatory Cytokine Responses after Traumatic Brain Injury in a Human Stem Cell Model
Source: J Neurotrauma. 2018 Jan 15;35(2):341–52. doi: 10.1089/neu.2017.5155 (PMC5784793; doi:10.1089/neu.2017.5155)
Supplement: Supplemental data [file Supp_Data.zip › Supp_Data.pdf]

Supplementary Data

List of abbreviations

CCL = CC chemokine ligands, EGF = Epidermal growth factor, FGF2 = Fibroblast Growth Factor 2, Flt3lig = Fms-related tyrosine kinase 3 ligand, G-CSF = Granulocyte colony-stimulating factor, GM-CSF = Granulocyte-macrophage colony-stimulating factor, GRO = chemokine (C-X-C motif) ligand 1 (CXCL1), IFN = Interferon, IL = Interleukin, IL-1ra = Interleukin-1 receptor antagonist, IP-10/IP10 = Interferon gamma-induced protein 10 (also known as C-X-C motif chemokine 10 (CXCL10)), MCP-1 = Monocyte chemotactic protein 1 (also known as CCL2), MCP-3 = Monocyte chemotactic protein-3 (also known as CCL7), MDC, Macrophage-Derived Chemokine (also known as CCL22), MIP1**α** = Macrophage inflammatory protein 1 alpha (also known as CCL3), MIP1**β** = Macrophage inflammatory protein 1 beta (also known as CCL4), PDGF = Platelet-derived growth factor, RANTES = regulated on activation, normal T cell expressed and secreted (also known as CCL5), sCD40L = soluble CD40 ligand, sIL-2Ra = Soluble Interleukin-2 receptor antagonist, TNF = Tumor necrosis factor, VEGF = Vascular Endothelial Growth Factor

SUPPLEMENTARY FIG. S1. Illustrates IL-1*β*, IL-6, and TNF induction in histograms with mean ± standard error of mean for all the cytokines measured to the left on each page. The colors signify escalating concentrations, same as seen in Figure 3. The novel blue bar is vehicle solution/baseline levels for each cytokine. To the right in each picture, the two-way mixed ANOVA analyses are illustrated as well as appropriate *post hoc* tests with *p* values as described in the statistical analysis. EGF, epidermal growth factor; ANOVA, analysis of variance.

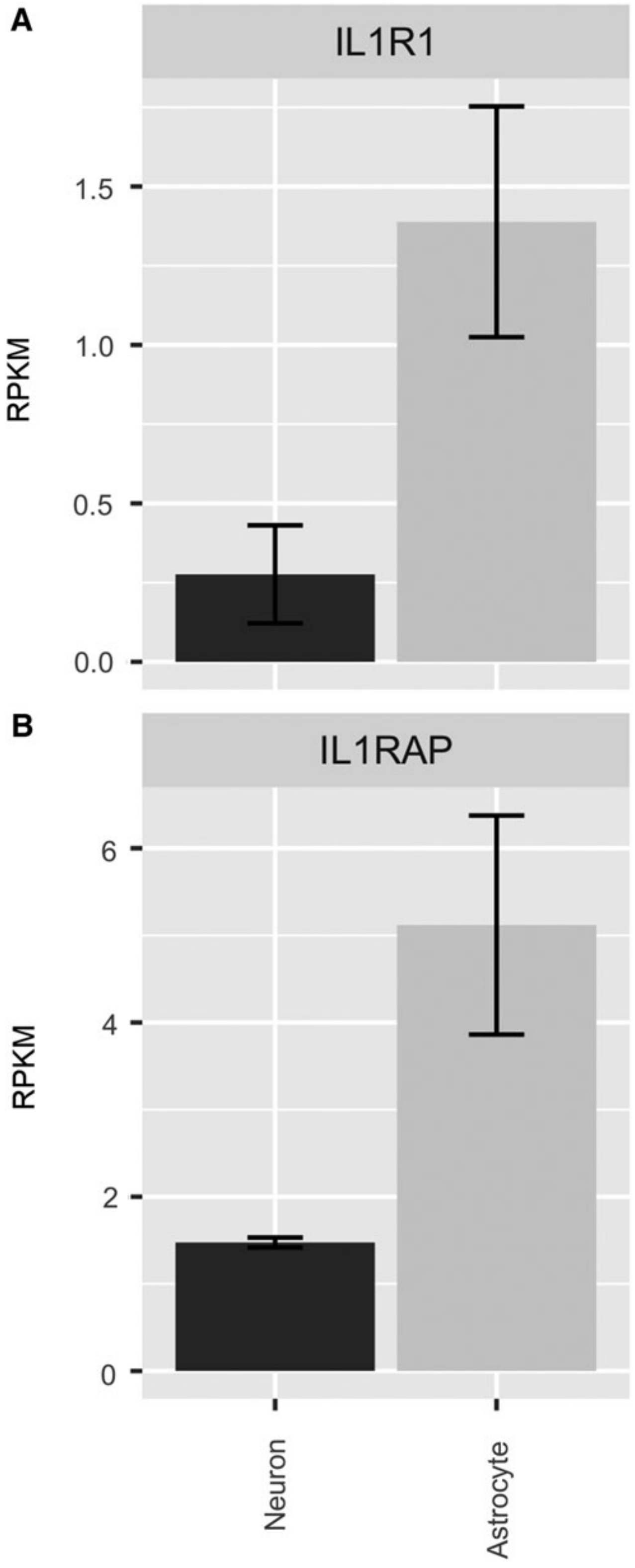

SUPPLEMENTARY FIG. S2. Bar plots illustrating a transcriptional analysis of interleukin 1 receptor proteins' mRNA levels between nonstimulated motor neuronal and astrocyte cultures. Neurons were analyzed after 17 days of terminal differentiation, technical $n=3$ (from two biological control lines). Astrocytes were analyzed after 28 days after terminal differentiation, technical $n=6$ (from two biological control lines, in triplicate). (**A**) Represents RPKM change of IL1R while (**B**) shows IL1RAP. Error bars represent mean ± standard error of the mean. RPKM, reads per kilobase of transcript per nillion mapped reads; IL1R1, interleukin 1 receptor 1; IL1RAP, interleukin 1 receptor accessory protein.

Supplementary Table S1. Patient Demographics

Patient	*Age*	*Sex*	*Injury mechanism*	*Post-injury GCS*	*Type of injury*
Patients used to determine temporal profile of cytokines					
1	28	Male	Fall down stairs	E1 V1 M2 = 4	Diffuse
2	28	Male	Road traffic accident	E2 V1 M5 = 8	Diffuse
3	26	Male	Road traffic accident	E2 V1 M4 = 7	Diffuse
4	35	Male	Assault	E1 V1 M4 = 6	Focal mass lesion
5	52	Female	Road traffic accident	E1 V1 M5 = 7	Diffuse
6	18	Female	Road traffic accident	E1 V1 M1 = 3	Diffuse
7	44	Female	Road traffic accident	E1 V1 M1 = 3	Diffuse
8	25	Male	Road traffic accident	E1 V1 M1 = 3	Diffuse
9	27	Female	Road traffic accident	E1 V1 M1 = 3	Diffuse
10	25	Female	Road traffic accident	E1 V1 M2 = 4	Diffuse
11	58	Female	Road traffic accident	E1 V1 M1 = 3	Diffuse
12	61	Female	Road traffic accident	E1 V1 M1 = 3	Diffuse
Patients used to assess cytokine levels					
1	44	Female	Road traffic accident	E1 V1 M1 = 3	Diffuse
2	25	Male	Road traffic accident	E1 V1 M1 = 3	Diffuse
3	27	Female	Road traffic accident	E1 V1 M1 = 3	Diffuse
4	25	Female	Road traffic accident	E1 V1 M2 = 4	Diffuse
5	58	Female	Road traffic accident	E1 V1 M1 = 3	Focal mass lesion
6	61	Female	Road traffic accident	E1 V1 M1 = 3	Focal mass lesion
7	49	Male	Road traffic accident	E1 V2 M5 = 8	Focal mass lesion
8	60	Male	Road traffic accident	E1 V1 M5 = 7	Diffuse
9	30	Male	Fall	E1 V2 M2 = 5	Focal mass lesion
10	39	Female	Road traffic accident	E1 V1 M1 = 3	Diffuse

GCS, Glasgow Coma Scale; E, eye component; V = verbal component; M, motor component.
Diffuse injury, Marshall CT classification grade II–IV; focal mass lesion, Marshall CT classification grade VI.

The columns with bold borders represent each of the experimental conditions (IL-1β, IL-6, and TNF). Within these headings, three types of contrast are tested in a two-way mixed analysis of variance (ANOVA): The effect of concentration (Conc), the effect of time, and the interaction between time and concentration (Time*Concentration). The concentration dependent induction of a given cytokine is highlighted with an ''X''. As time is a repeated measure within the mixed ANOVA, the contrast can be described by a range of polynomial models: linear (L), quadratic (Q), and cubic (C). The model that fits the data most accurately is presented in the relevant column. If more than one generates the same p value within the model, each of the different models is listed—e.g., L/Q means that both linear and quadratic models fit the data with an equally significant p value.

Supplementary Table S2. Summary of Experimental Conditions Tested between Time and Varying Concentration of Added Cytokine to the In Vitro Model

	Experimental conditions and interaction tested								
	IL-1β Added			*IL-6 Added*			*TNF-α Added*		
Cytokine measured	Conc	*Time*	*Time*Conc*	*Conc*	*Time*	*Time*Conc*	Conc	*Time*	*Time*Conc*
EGF				X	L/Q	L		Q	
Eotaxin		L/Q/C			L/Q/C		X	L	L
FGF2		C		X				C	
Flt3lig		Q			Q/C			Q/C	
Fractalkine		Q		X	Q	Q		Q	
GCSF		L/Q/C			L/Q/C			L/Q/C	
GMCSF		L/Q/C		X	L/Q/C	L	X	L/Q/C	L
GRO		Q/C			Q/C		X	L/Q	L/Q
IFNa2		C		X	L/C			C	
IFNg		L/Q/C		X	L/Q/C	L/Q/C		Q/C	
IL-1a		L		X	C	L	X	L	L
IL-1b					Q			Q	
IL-1ra		C			C			C	
IL-2		L/Q/C			L/Q/C			L/Q/C	
IL-3					Q				
IL-4				X	L	L			
IL-5		Q			Q			Q	
IL-6		C							
IL-7		C		X	L/Q/C	L/Q	X	L	L
IL-8		C			C		X	L	L
IL-9									
IL-10		C			L/C			C	
IL-12p40		Q/C		X	L/Q/C	L		L/Q/C	
IL-12p70		C		X	C			C	
IL-13		L/Q/C						L/Q/C	
IL-15		Q			Q			Q	
IL-17		L/Q/C			L/Q/C			L/Q/C	
IP10	X	L	L		L		X	L	L
MCP-1		C			C			C	
MCP-3		Q/C			Q/C		X	L	L
MDC		Q/C	C		Q/L			Q/C	
MIP1α		Q			Q			Q	
MIP1β		C	Q	X	C	Q	X	L/C	C
PDGFAA		C			L/Q/C			C	
PDGFABBB		C						C	
RANTES					C		X	L/Q	L/Q
sCD40L		L		X	C		X	Q	L
sIL-2Ra		L/Q/C		X	Q/C			Q/C	
TNF		Q			Q/C	Q			
TNF-b		Q/C		X	L/Q/C	L		C	
VEGF		C			C			C	

EGF, epidermal growth factor; FGF2, fibroblast growth factor 2; Flt3lig, Fms-related tyrosine kinase 3 ligand; G-CSF, granulocyte colony-stimulating factor; GM-CSF, granulocyte-macrophage colony-stimulating factor; GRO, chemokine (C-X-C motif) ligand 1 (CXCL1); IFN, interferon; IL, interleukin; IL-1ra, interleukin-1 receptor antagonist; IP-10/IP10, interferon gamma-induced protein 10 (also known as C-X-C motif chemokine 10 (CXCL10)); MCP-1, monocyte chemotactic protein 1 (also known as CCL2); MCP-3, monocyte chemotactic protein-3 (also known as CCL7); MDC, macrophage-derived chemokine (also known as CCL22); MIP1α, macrophage inflammatory protein 1 alpha (also known as CCL3); MIP1β, macrophage inflammatory protein 1 beta (also known as CCL4); PDGF, platelet-derived growth factor; RANTES, regulated on activation, normal T cell expressed and secreted (also known as CCL5); sCD40L, soluble CD40 ligand; sIL-2Ra, soluble Interleukin-2 receptor antagonist; TNF, tumor necrosis factor; VEGF, vascular endothelial growth factor.
