# Supplementary material for: Elucidating Pro-Inflammatory Cytokine Responses after Traumatic Brain Injury in a Human Stem Cell Model
Source: J Neurotrauma. 2018 Jan 15;35(2):341–52. doi: 10.1089/neu.2017.5155 (PMC5784793; doi:10.1089/neu.2017.5155)
Supplement: Supplemental data [file Supp_Data.zip › Supp_Fig1.pdf]

EGF

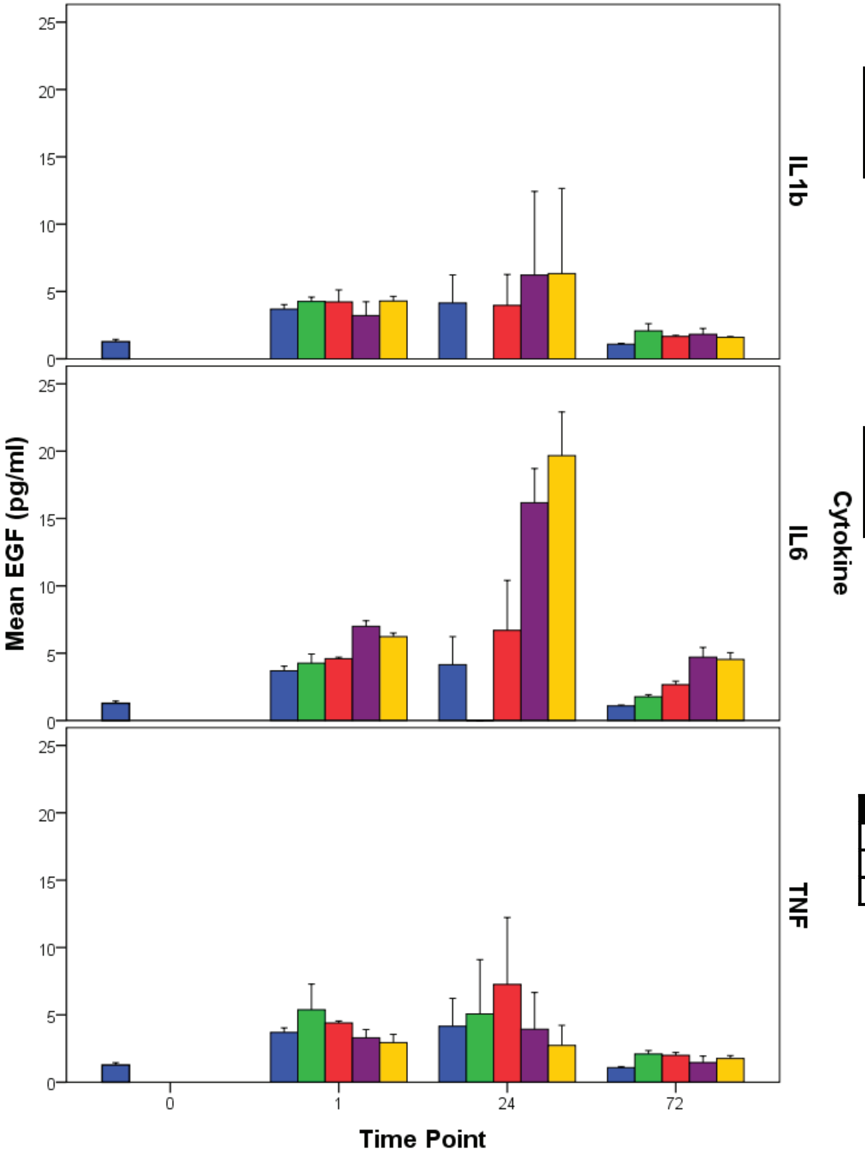

Two Way Mixed Anova

aGreenhouse-Geisser

Post Hoc Tests

bwithin well contrast with most stringent p-value (Linear vs Quadratic vs Cubic

Effect	ANOVA significance
**Time[a]**	0.142
**Time*Concentration[a]**	0.793
**Concentration**	0.896

[a]Greenhouse-Geisser

Bonferroni: Differences Between Concentrations

Concentration	1	2	3	4
0	1	1	0.004	0.002
1		0.548	0.004	0.002
2			0.34	0.011
3				1

Effect	ANOVA significance
**Time[a]**	<0.0001
**Time*Concentration[a]**	0.003
**Concentration**	<0.0001

[a]Greenhouse-Geisser

Polynomial Model: Differences in Time and Interaction of Time and Concentration

Effect	Best Model of Contrast[b]	Signficance[a]
**Time**	Linear/Quadratic	<0.0001
**Time*Concentration**	Linear	<0.0001

[b]Within-well contrast with most stringent p-value (Linear vs Quadratic vs Cubic)

Effect	ANOVA significance
**Time[a]**	0.042
**Time*Concentration[a]**	0.910
**Concentration**	0.760

Polynomial Model: Differences in Time and Interaction of Time and Concentratior

Effect	Best Model of Contrast[b]	Signficance[a]
**Time**	Quadratic	0.008

Effect of Added Cytokine: Multivariate ANOVA

Time Point	1 Hour	24 Hours	72 Hours
**Significance[a]**	<0.0001	<0.0001	<0.0001

Post Hoc Tests

EGF 1 h	IL1β	IL6	TNF
None	1	0.348	1
IL1β		0.086	1
IL6			0.088

EGF 24 h	IL1β	IL6	TNF
None	1	1	1
IL1β		0.211	1
IL6			0.332

EGF 72 h	IL1β	IL6	TNF
None	1	0.003	1
IL1β		0.001	1
IL6			0.001

Eotaxin

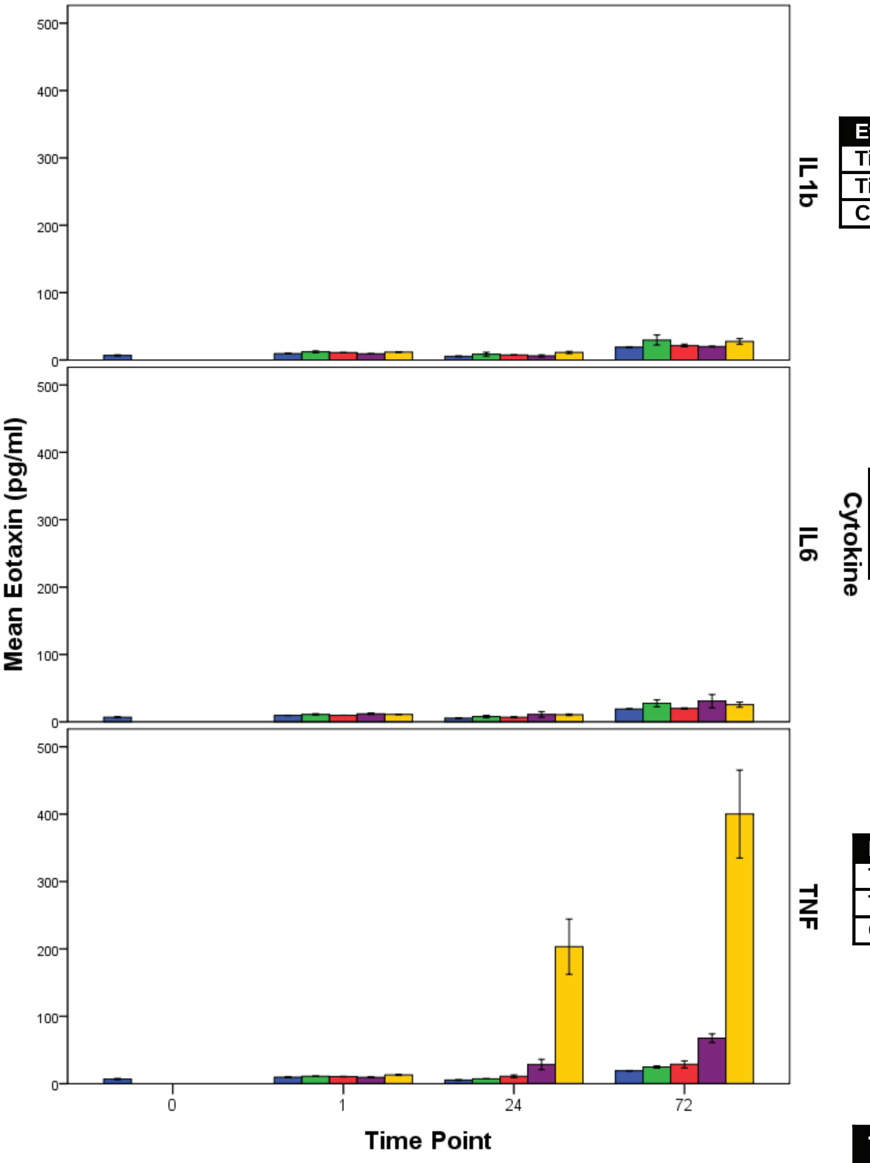

Two Way Mixed Anova
[a]Greenhouse-Geisser

Post Hoc Tests
[b]within well contrast with most stringent p-value (Linear vs Quadratic vs Cubic

Effect	ANOVA significance
Time[a]	<0.0001
Time*Concentration[a]	0.340
Concentration	0.211

Polynomial Model: Differences in Time and Interaction of Time and Concentration

Effect	Best Model of Contrast[b]	Signficance[a]
Time	Linear/Quadratic/Cubic	<0.0001

Effect	ANOVA significance
Time[a]	<0.0001
Time*Concentration[a]	0.508
Concentration	0.474

Polynomial Model: Differences in Time and Interaction of Time and Concentration

Effect	Best Model of Contrast[b]	Signficance[a]
Time	Linear/Quadratic/Cubic	<0.0001

Effect	ANOVA significance
Time[a]	<0.0001
Time*Concentration[a]	<0.0001
Concentration	<0.0001

Bonferroni: Differences Between Concentrations

Concentration	1	2	3	4
0	1	1	1	<0.0001
1		1	1	<0.0001
2			1	<0.0001
3				<0.0001

Polynomial Model: Differences in Time and Interaction of Time and Concentration

Effect	Best Model of Contrast[b]	Signficance[a]
Time	Linear	<0.0001
Time*Concentration	Linear	<0.0001

Effect of AddedCytokine

Time Point	1 Hour	24 Hours	72 Hours
Significance[a]	<0.0001	0.004	0.001

Post Hoc Tests

Eotaxin 1 h	IL1β	IL6	TNF
None	0.773	0.949	0.924
IL1β		1	1
IL6			1

Eotaxin 24 h	IL1β	IL6	TNF
None	1	1	0.548
IL1β		1	0.081
IL6			0.087

Eotaxin 72 h	IL1β	IL6	TNF
None	1	1	0.489
IL1β		1	0.065
IL6			0.07

FGF2

Two Way Mixed Anova

[a]Greenhouse-Geisser

Post Hoc Tests

[b]within well contrast with most stringent p-value (Linear vs Quadratic vs Cubic

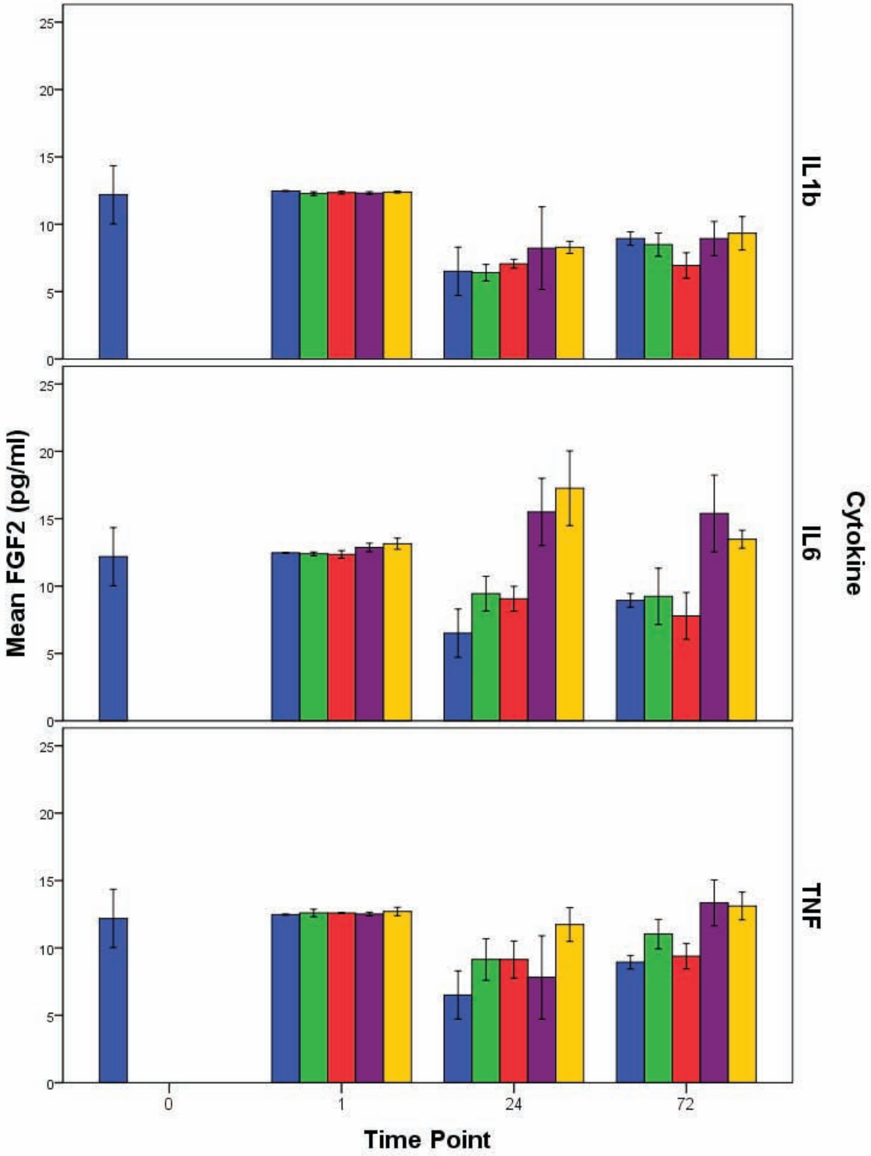

Effect	ANOVA significance
Time[a]	0.001
Time*Concentration[a]	0.977
Concentration	0.836

Polynomial Model: Differences in Time and Interaction of Time and Concentration

Effect	Best Model of Contrast[b]	Signficance[a]
Time	Cubic	<0.0001

Effect	ANOVA significance
Time[a]	0.451
Time*Concentration[a]	0.213
Concentration	0.002

Bonferroni: Differences Between Concentrations

Concentration	1	2	3	4
0	1	1	0.015	0.014
1		1	0.061	0.058
2			0.026	0.025
3				1

Effect	ANOVA significance
Time[a]	0.022
Time*Concentration[a]	0.724
Concentration	0.239

Polynomial Model: Differences in Time and Interaction of Time and Concentration

Effect	Best Model of Contrast[b]	Signficance[a]
Time	Cubic	0.001

Effect of Added Cytokine: Multivariate ANOVA

Time Point	1 Hour	24 Hours	72 Hours
Significance	<0.0001	<0.0001	<0.0001

Post Hoc Tests

FGF2 1 hour	IL1β	IL6	TNF
None	1	1	1
IL1β		0.167	0.555
IL6			1

FGF2 24 hours	IL1β	IL6	TNF
None	1	0.064	1
IL1β		0.006	1
IL6			0.177

FGF2 72 hours	IL1β	IL6	TNF
None	1	1	0.971
IL1β		0.11	0.068
IL6			1

Flt3lig

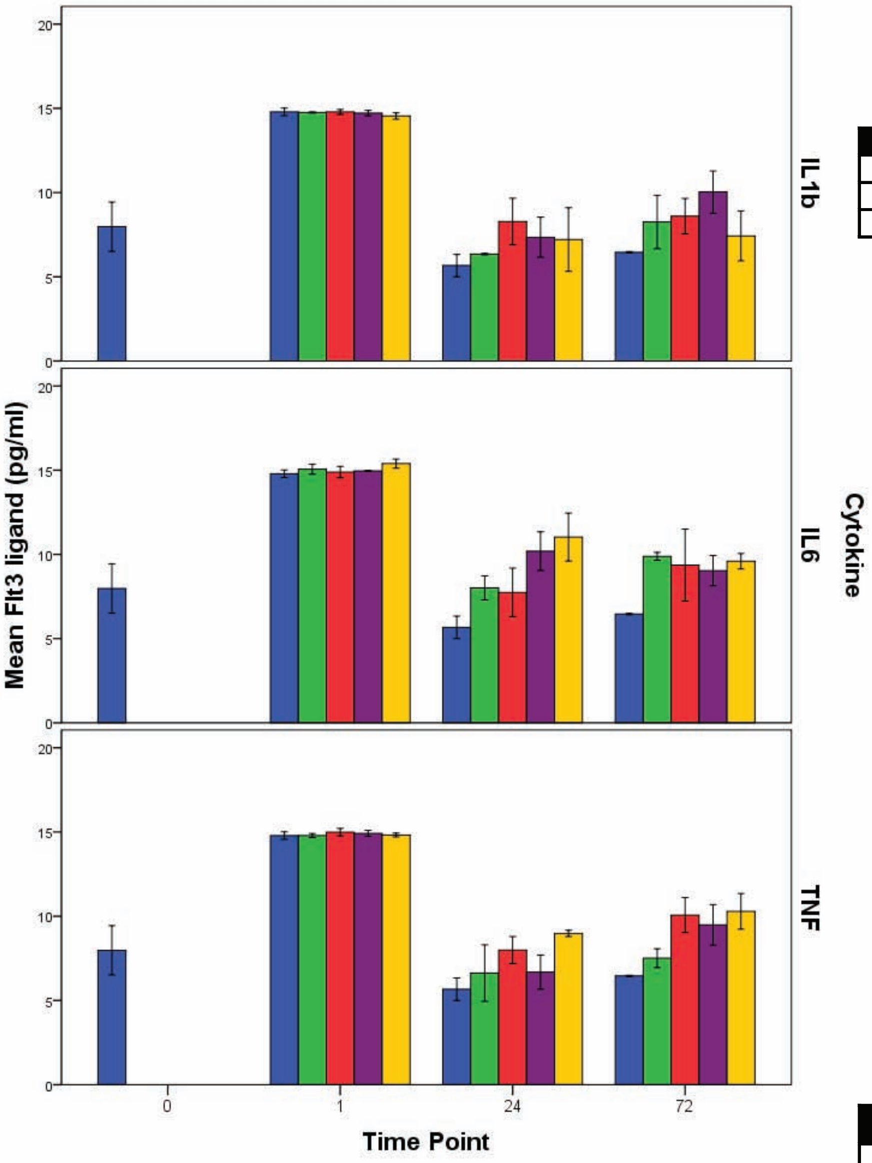

Two Way Mixed Anova

[a]Greenhouse-Geisser

Post Hoc Tests

[b]within well contrast with most stringent p-value (Linear vs Quadratic vs Cubic

Effect	ANOVA significance
Time[a]	<0.0001
Time*Concentration[a]	0.793
Concentration	0.717

Polynomial Model: Differences in Time and Interaction of Time and Concentration

Effect	Best Model of Contrast[b]	Signficance[a]
Time	Quadratic/Cubic	<0.0001

Effect	ANOVA significance
Time[a]	<0.0001
Time*Concentration[a]	0.441
Concentration	0.113

Polynomial Model: Differences in Time and Interaction of Time and Concentration

Effect	Best Model of Contrast[b]	Signficance[a]
Time	Quadratic/Cubic	<0.0001

Effect	ANOVA significance
Time[a]	<0.0001
Time*Concentration[a]	0.595
Concentration	0.156

Post Hoc Tests

Polynomial Model: Differences in Time and Interaction of Time and Concentration

Effect	Best Model of Contrast[b]	Signficance[a]
Time	Quadratic/Cubic	<0.0001

Effect of Added Cytokine: Multivariate ANOVA

Time Point	1 Hour	24 Hours	72 Hours
Significance	<0.0001	<0.0001	<0.0001

Post Hoc Tests

Flt3lig 1h	IL1β	IL6	TNF
None	1	1	1
IL1β		0.054	1
IL6			0.966

Flt3lig 24h	IL1β	IL6	TNF
None	1	0.063	0.96
IL1β		0.156	1
IL6			0.319

Flt3lig 72h	IL1β	IL6	TNF
None	0.57	0.12	0.153
IL1β		1	0.57
IL6			1

Fractalkine

Two Way Mixed Anova

[a]Greenhouse-Geisser

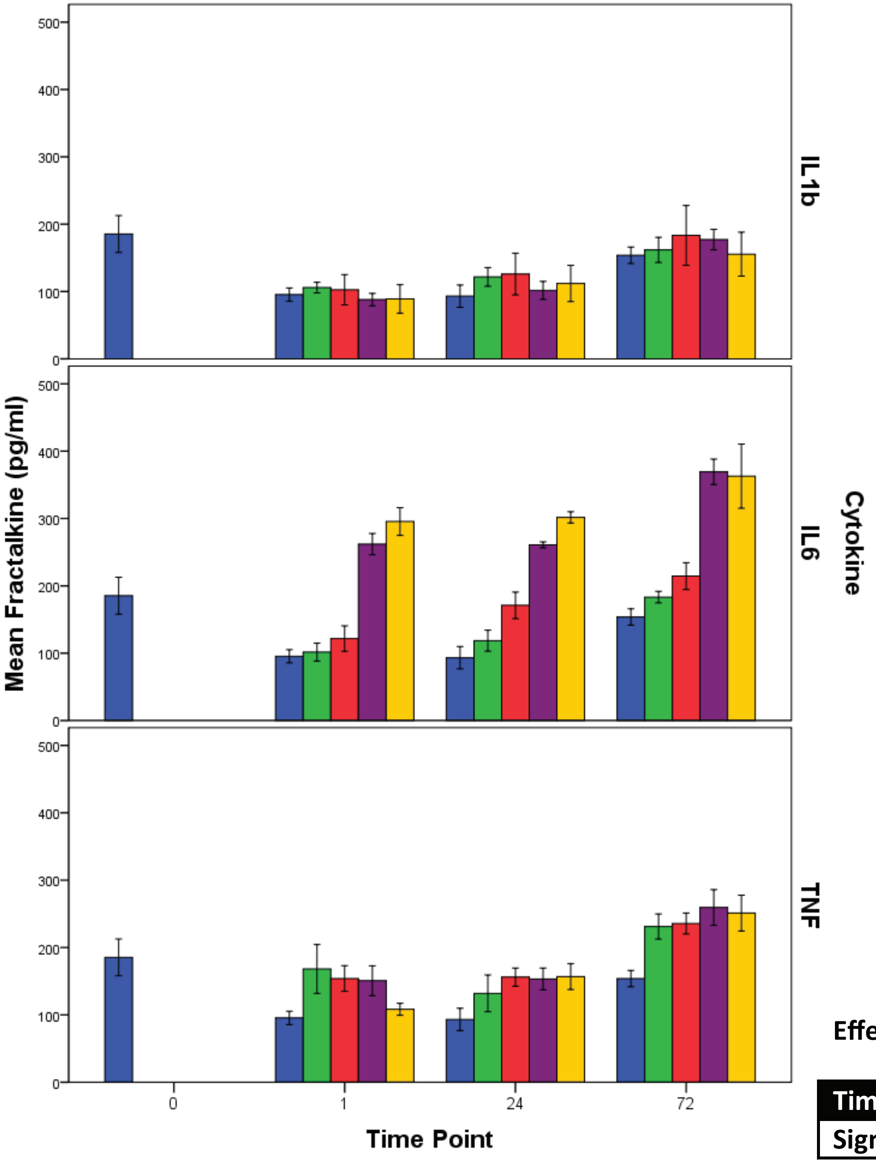

Effect	ANOVA significance
Time[a]	<0.0001
Time*Concentration[a]	0.945
Concentration	0.948

Effect	ANOVA significance
Time[a]	<0.0001
Time*Concentration[a]	0.002
Concentration	<0.0001

Effect	ANOVA significance
Time[a]	<0.0001
Time*Concentration[a]	0.338
Concentration	0.105

Post Hoc Tests

[b]within well contrast with most stringent p-value (Linear vs Quadratic vs Cubic

Polynomial Model: Differences in Time and Interaction of Time and Concentration

Effect	Best Model of Contrast[b]	Signficance[a]
Time	Quadratic	<0.0001

Bonferroni: Differences Between Concentrations

Concentration	1	2	3	4
0	1	0.368	<0.0001	<0.0001
1		1	<0.0001	<0.0001
2			0.002	0.001
3				1

Polynomial Model: Differences in Time and Interaction of Time and Concentration

Effect	Best Model of Contrast[b]	Signficance[a]
Time	Quadratic	<0.0001
Time*Concentration	Quadratic	0.005

Polynomial Model: Differences in Time and Interaction of Time and Concentration

Effect	Best Model of Contrast[b]	Signficance[a]
Time	Quadratic	<0.0001

Effect of Added Cytokine: Multivariate ANOVA

Time Point	1 Hour	24 Hours	72 Hours
Significance	<0.0001	<0.0001	<0.0001

Post Hoc Tests

Frac 1h	IL1β	IL6	TNF
None	1	0.075	1
IL1β		0.001	0.294
IL6			0.268

Frac 24h	IL1β	IL6	TNF
None	1	0.006	0.591
IL1β		<0.0001	0.675
IL6			0.028

Frac 72h	IL1β	IL6	TNF
None	1	0.021	0.205
IL1β		0.001	0.04
IL6			0.909

GCSF

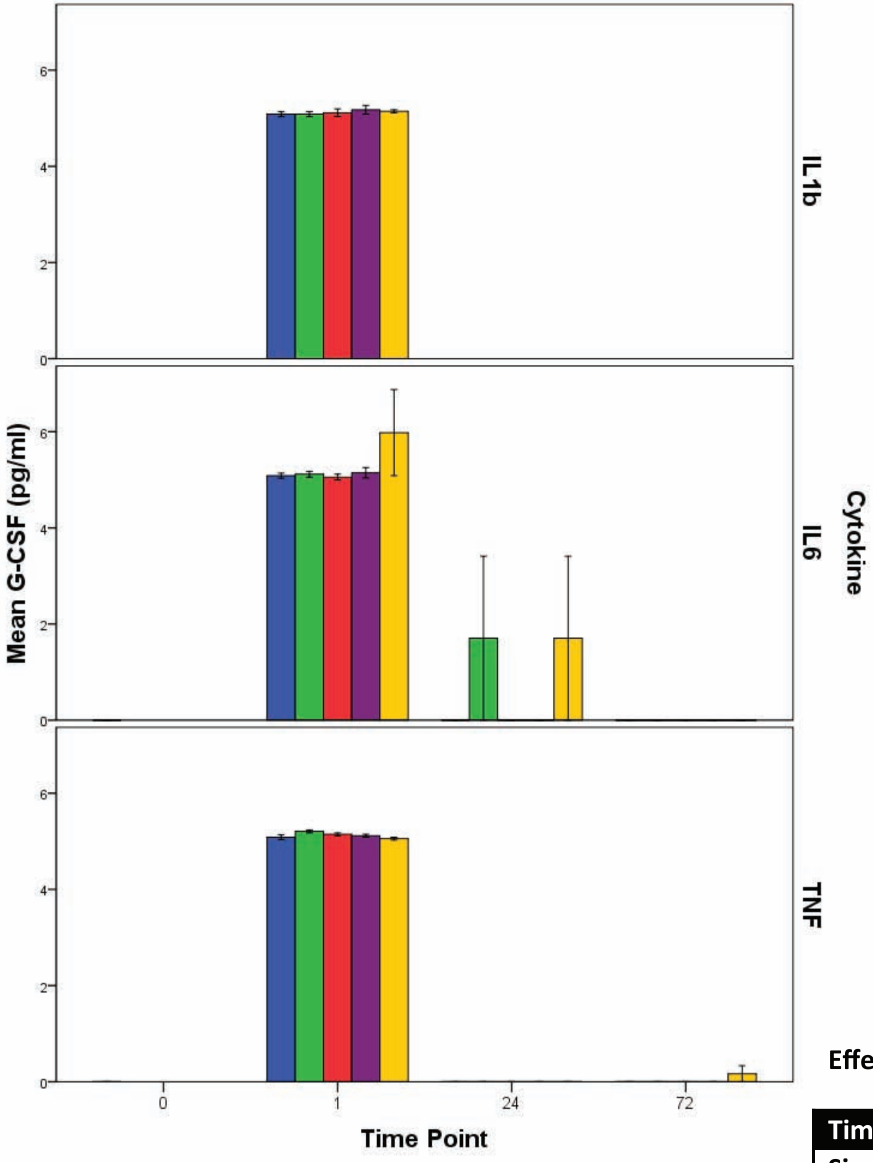

Two Way Mixed Anova

[a]Greenhouse-Geisser

Effect	ANOVA significance
Time[a]	<0.0001
Time*Concentration[a]	0.825
Concentration	0.825

Effect	ANOVA significance
Time[a]	<0.0001
Time*Concentration[a]	0.574
Concentration	0.574

Effect	ANOVA significance
Time[a]	<0.0001
Time*Concentration[a]	0.264
Concentration	0.607

Post Hoc Tests

[b]within well contrast with most stringent p-value (Linear vs Quadratic vs Cubic

Polynomial Model: Differences in Time and Interaction of Time and Concentration

Effect	Best Model of Contrast[b]	Signficance[a]
Time	Linear/Quadratic/Cubic	<0.0001

Polynomial Model: Differences in Time and Interaction of Time and Concentration

Effect	Best Model of Contrast[b]	Signficance[a]
Time	Linear/Quadratic/Cubic	<0.0001

Polynomial Model: Differences in Time and Interaction of Time and Concentration

Effect	Best Model of Contrast[b]	Signficance[a]
Time	Linear/Quadratic/Cubic	<0.0001

Effect of Added Cytokine: Multivariate ANOVA

Time Point	1 Hour	24 Hours	72 Hours
Significance	<0.0001	0.161	0.536

Post Hoc Tests

GCSF 1h	IL1β	IL6	TNF
None	1	1	1
IL1β		1	1
IL6			1

GMCSF

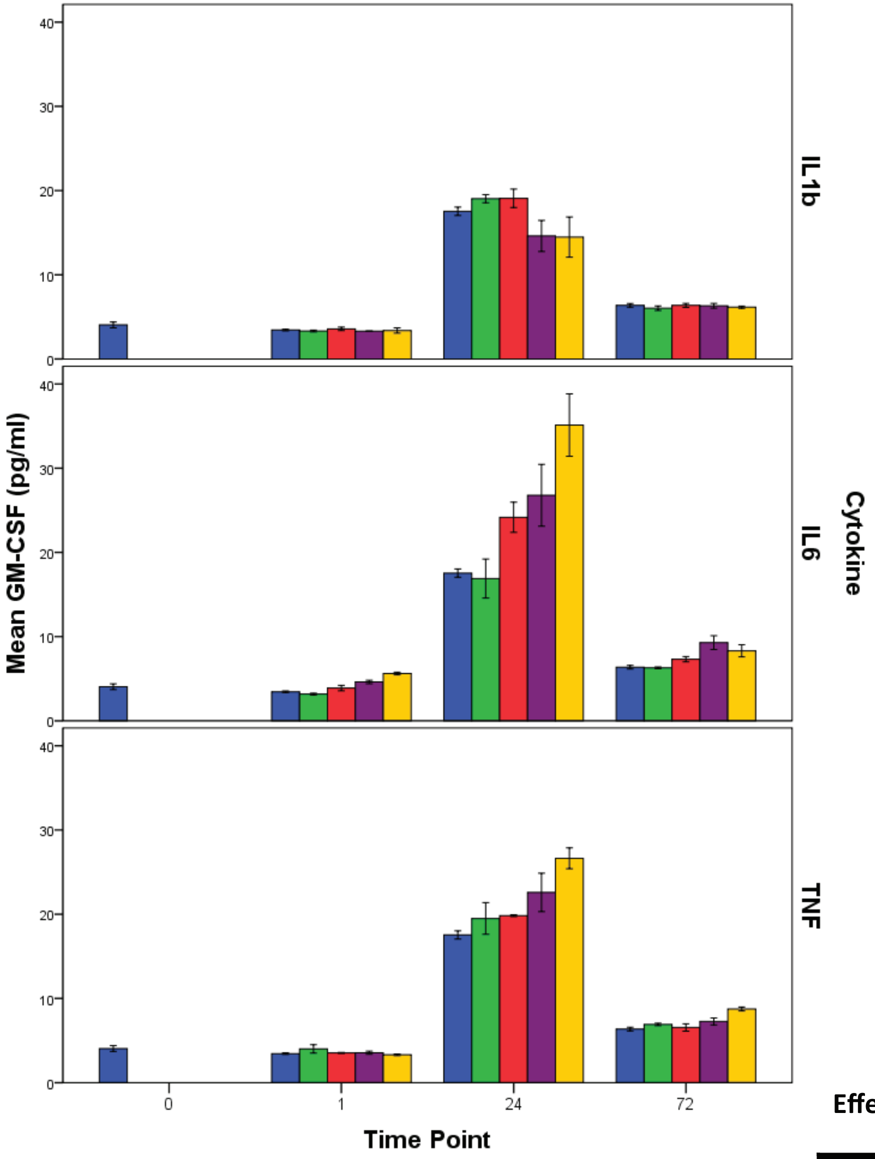

Two Way Mixed Anova

[a]Greenhouse-Geisser

Post Hoc Tests

[b]within well contrast with most stringent p-value (Linear vs Quadratic vs Cubic

IL1b

Effect	ANOVA significance
Time[a]	<0.0001
Time*Concentration[a]	0.125
Concentration	0.153

Polynomial Model: Differences in Time and Interaction of Time and Concentration

Effect	Best Model of Contrast[b]	Signficance[a]
Time	Linear/Quadratic/Cubic	<0.0001

IL6

Effect	ANOVA significance
Time[a]	<0.0001
Time*Concentration[a]	0.009
Concentration	<0.0001

Bonferroni: Differences Between Concentrations

Concentration	1	2	3	4
0	1	0.318	0.02	0.001
1		0.188	0.012	<0.0001
2			1	0.017
3				0.265

Polynomial Model: Differences in Time and Interaction of Time and Concentration

Effect	Best Model of Contrast[b]	Signficance[a]
Time	Linear/Quadratic/Cubic	<0.0001
Time*Concentration	Linear	<0.001

TNF

Effect	ANOVA significance
Time[a]	<0.0001
Time*Concentration[a]	0.006
Concentration	0.015

Post Hoc Tests

Bonferroni: Differences Between Concentrations

Concentration	1	2	3	4
0	1	1	0.47	0.017
1		1	1	0.114
2			1	0.082
3				0.761

Polynomial Model: Differences in Time and Interaction of Time and Concentration

Effect	Best Model of Contrast[b]	Signficance[a]
Time	Linear/Quadratic/Cubic	<0.0001
Time*Concentration	Linear	<0.001

Effect of Added Cytokine: Multivariate ANOVA

Time Point	1 Hour	24 Hours	72 Hours
Significance	<0.0001	<0.0001	<0.0001

Post Hoc Tests

GMCSF 1h	IL1β	IL6	TNF
None	1	0.251	1
IL1β		0.007	1
IL6			0.056

GMCSF 24h	IL1β	IL6	TNF
None	1	0.142	1
IL1β		0.001	0.121
IL6			0.655

GMCSF 72h	IL1β	IL6	TNF
None	1	0.2	0.786
IL1β		0.003	1
IL6			0.056

GRO

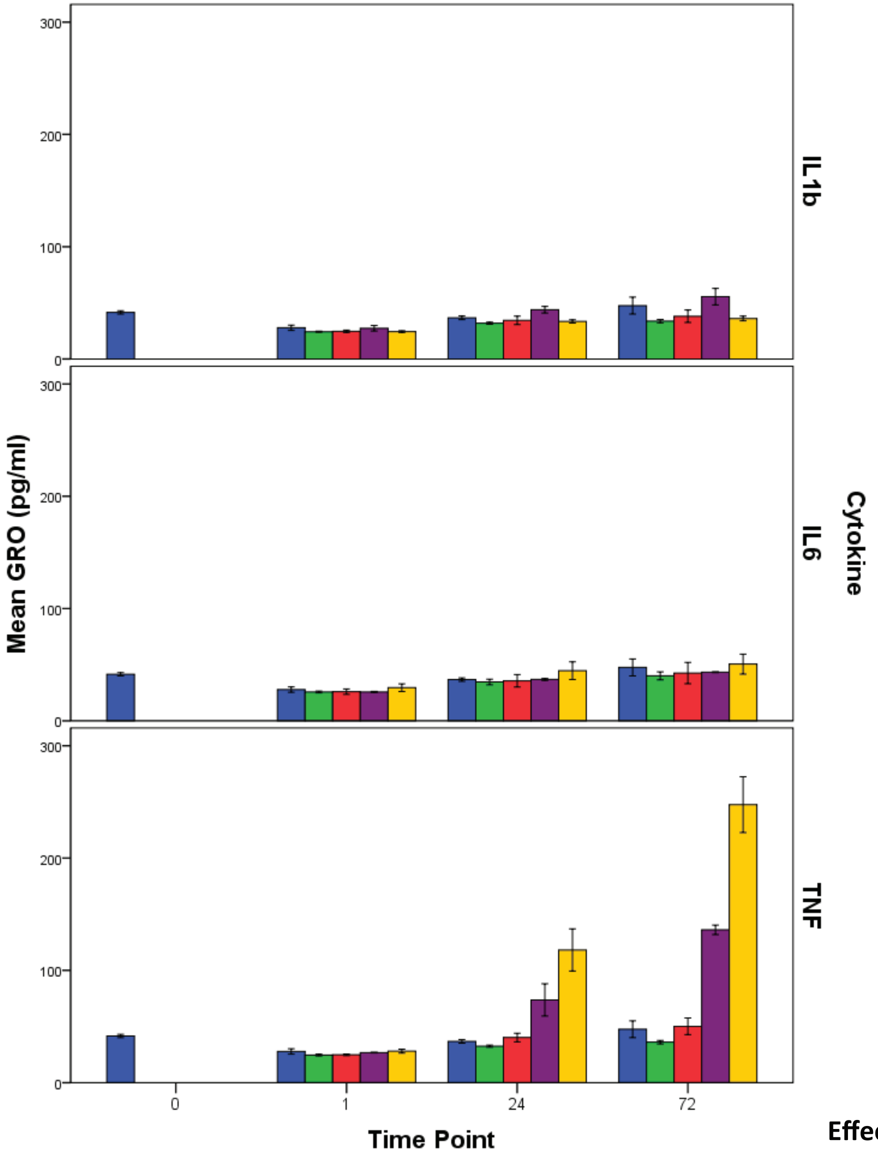

Two Way Mixed Anova
[a]Greenhouse-Geisser

Post Hoc Tests
[b]within well contrast with most stringent p-value (Linear vs Quadratic vs Cubic

IL1b

Effect	ANOVA significance
Time[a]	<0.0001
Time*Concentration[a]	0.081
Concentration	0.079

Polynomial Model: Differences in Time and Interaction of Time and Concentration

Effect	Best Model of Contrast[b]	Signficance[a]
Time	Quadratic/Cubic	<0.0001

IL6

Effect	ANOVA significance
Time[a]	<0.0001
Time*Concentration[a]	0.585
Concentration	0.781

Polynomial Model: Differences in Time and Interaction of Time and Concentration

Effect	Best Model of Contrast[b]	Signficance[a]
Time	Quadratic/Cubic	<0.0001

TNF

Effect	ANOVA significance
Time[a]	<0.0001
Time*Concentration[a]	<0.0001
Concentration	<0.0001

Bonferroni: Differences Between Concentrations

Concentration	1	2	3	4
0	1	1	0.012	1
1		1	0.004	<0.0001
2			0.014	<0.0001
3				0.002

Polynomial Model: Differences in Time and Interaction of Time and Concentration

Effect	Best Model of Contrast[b]	Signficance[a]
Time	Linear/Quadratic	<0.0001
Time*Concentration	Linear/Quadratic	<0.0001

Effect of Added Cytokine: Multivariate ANOVA

Time Point	1 Hour	24 Hours	72 Hours
Significance	<0.0001	<0.0001	<0.0001

Post Hoc Tests

GRO 1h	IL1β	IL6	TNF
None	0.989	1	1
IL1β		1	1
IL6			0.056

GRO 24h	IL1β	IL6	TNF
None	1	1	0.329
IL1β		1	0.016
IL6			0.029

GRO 72h	IL1β	IL6	TNF
None	1	1	0.255
IL1β		1	0.005
IL6			0.008

IFNa2

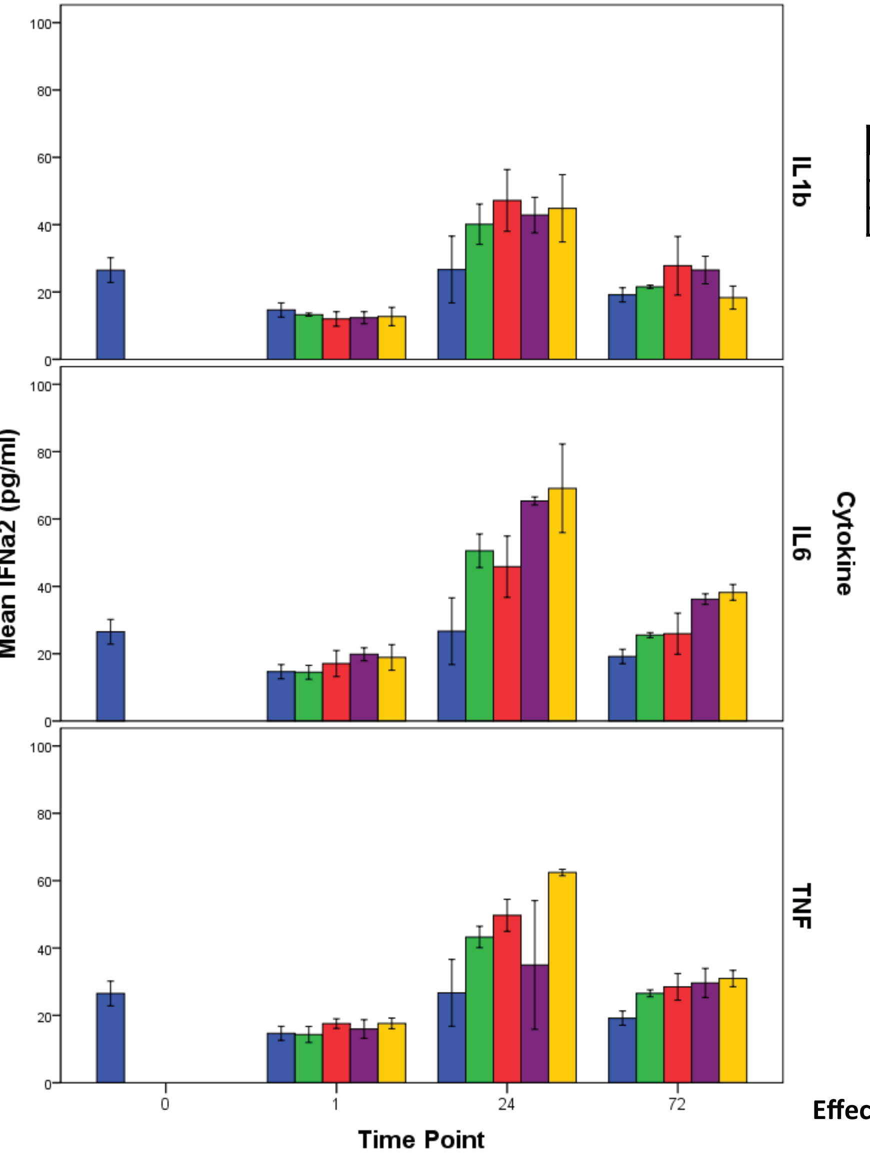

Two Way Mixed Anova

[a]Greenhouse-Geisser

Post Hoc Tests

[b]within well contrast with most stringent p-value (Linear vs Quadratic vs Cubic

IL1b

Effect	ANOVA significance
Time[a]	<0.0001
Time*Concentration[a]	0.330
Concentration	0.801

Polynomial Model: Differences in Time and Interaction of Time and Concentration

Effect	Best Model of Contrast[b]	Signficance[a]
Time	Cubic	<0.0001

IL6

Effect	ANOVA significance
Time[a]	<0.0001
Time*Concentration[a]	0.051
Concentration	0.029

Bonferroni: Differences Between Concentrations

Concentration	1	2	3	4
0	1	1	0.081	0.052
1		1	1	0.826
2			1	0.710
3				1

Polynomial Model: Differences in Time and Interaction of Time and Concentration

Effect	Best Model of Contrast[b]	Signficance[a]
Time	Linear/Cubic	<0.0001

TNF

Effect	ANOVA significance
Time[a]	<0.0001
Time*Concentration[a]	0.402
Concentration	0.108

Polynomial Model: Differences in Time and Interaction of Time and Concentration

Effect	Best Model of Contrast[b]	Signficance[a]
Time	Cubic	<0.0001

Effect of Added Cytokine: Multivariate ANOVA

Time Point	1 Hour	24 Hours	72 Hours
Significance	<0.0001	<0.0001	<0.0001

Post Hoc Tests

IFNa2 1h	IL1β	IL6	TNF
None	1	1	1
IL1β		0.02	0.137
IL6			1

IFNa2 24h	IL1β	IL6	TNF
None	0.602	0.025	0.278
IL1β		0.215	1
IL6			0.733

IFNa2 72h	IL1β	IL6	TNF
None	1	0.069	0.252
IL1β		0.062	0.455
IL6			1

IFNg

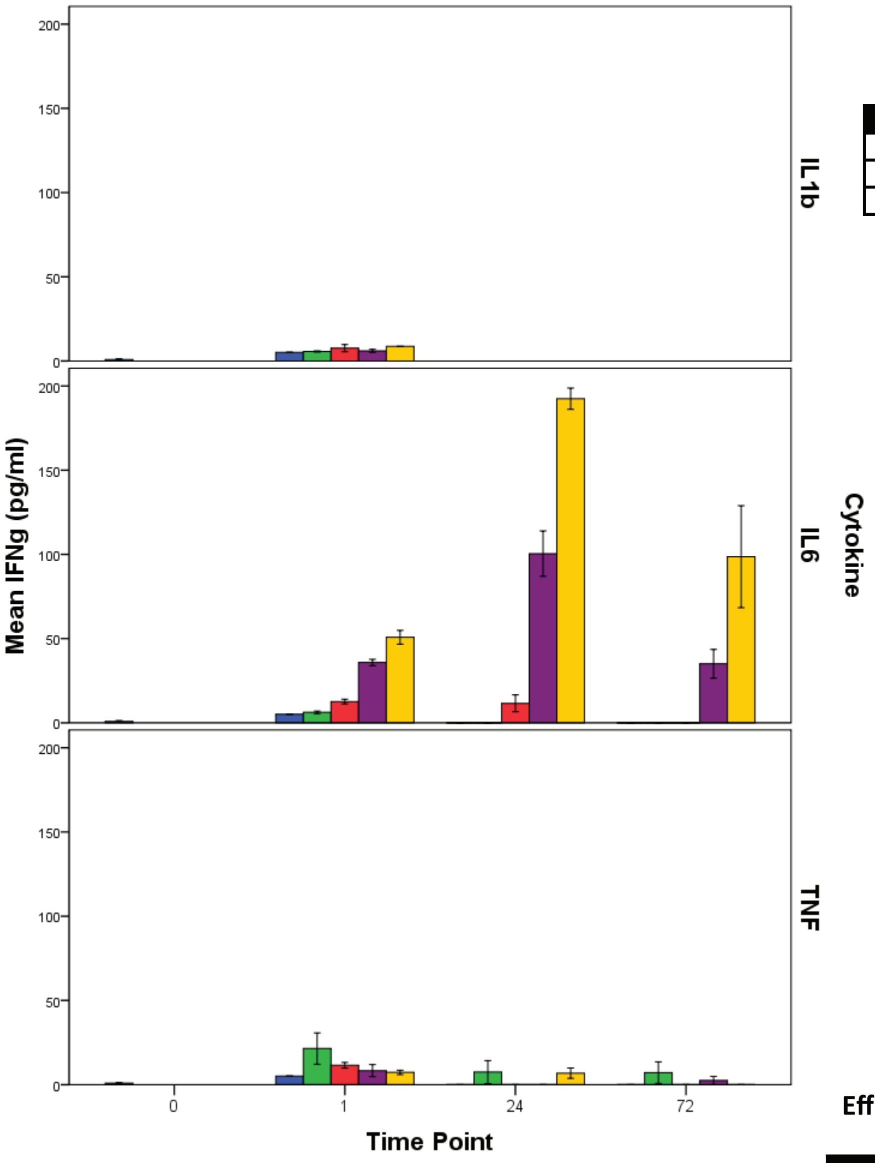

Two Way Mixed Anova

[a]Greenhouse-Geisser

Post Hoc Tests

[b]within well contrast with most stringent p-value (Linear vs Quadratic vs Cubic

IL1b

Effect	ANOVA significance
Time[a]	<0.0001
Time*Concentration[a]	0.220
Concentration	0.277

Polynomial Model: Differences in Time and Interaction of Time and Concentration

Effect	Best Model of Contrast[b]	Signficance[a]
Time	Linear/Quadratic/Cubic	<0.0001

IL6

Effect	ANOVA significance
Time[a]	<0.0001
Time*Concentration[a]	<0.0001
Concentration	<0.0001

Bonferroni: Differences Between Concentrations

Concentration	1	2	3	4
0	1	1	<0.0001	<0.0001
1		1	<0.0001	<0.0001
2			0.001	<0.0001
3				<0.0001

Polynomial Model: Differences in Time and Interaction of Time and Concentration

Effect	Best Model of Contrast[b]	Signficance[a]
Time	Linear/Quadratic/Cubic	<0.0001
Time*Concentration	Linear/Quadratic/Cubic	<0.0001

TNF

Effect	ANOVA significance
Time[a]	<0.0001
Time*Concentration[a]	0.066
Concentration	0.367

Polynomial Model: Differences in Time and Interaction of Time and Concentration

Effect	Best Model of Contrast[b]	Signficance[a]
Time	Quadratic/Cubic	<0.0001

Effect of Added Cytokine: Multivariate ANOVA

Time Point	1 Hour	24 Hours	72 Hours
Significance	<0.0001	<0.0001	0.004

Post Hoc Tests

IFNg 1h	IL1β	IL6	TNF
None	1	0.057	1
IL1β		0.002	1
IL6			0.038

IFNg 24h	IL1β	IL6	TNF
None	1	0.09	1
IL1β		0.002	1
IL6			0.003

IFNg 72h	IL1β	IL6	TNF
None	1	0.388	1
IL1β		0.029	1
IL6			0.05

IL10

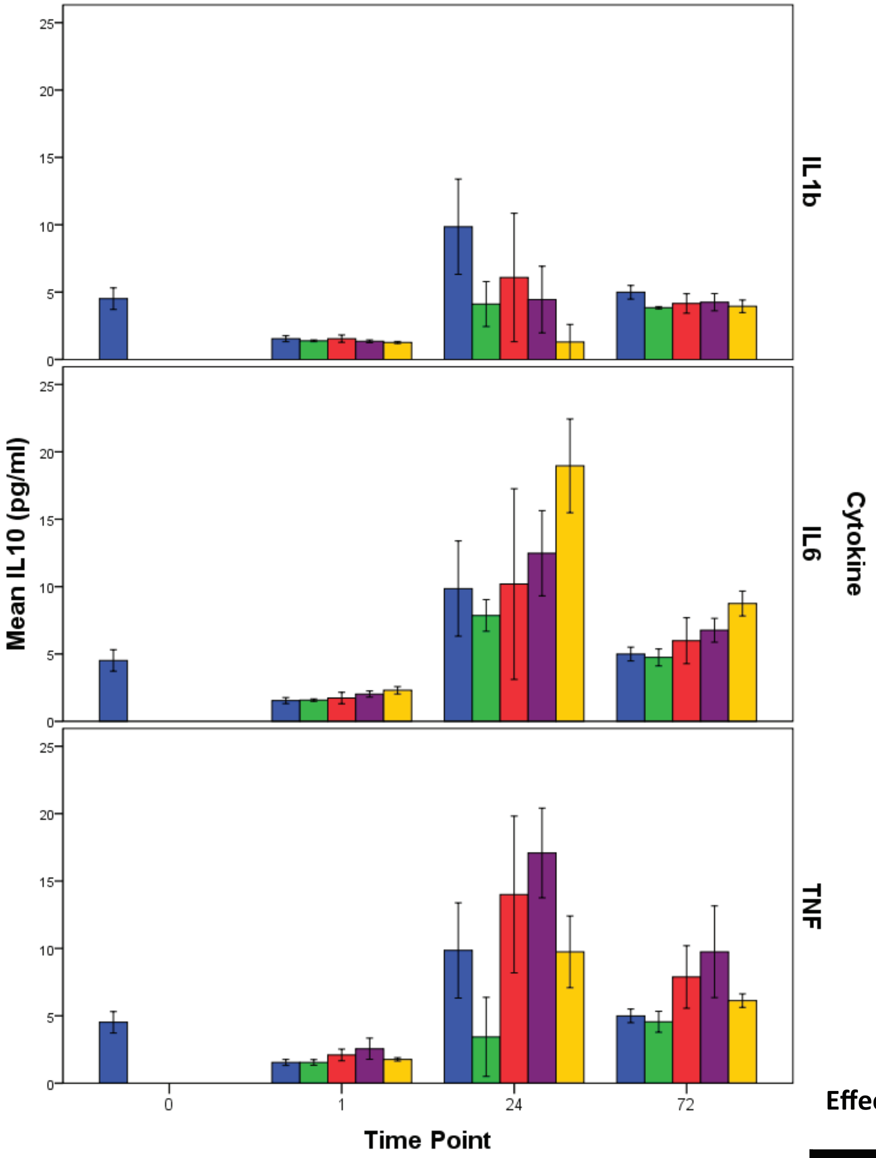

Two Way Mixed Anova

aGreenhouse-Geisser

Post Hoc Tests

bwithin well contrast with most stringent p-value (Linear vs Quadratic vs Cubic

Effect	ANOVA significance
Time[a]	0.026
Time*Concentration[a]	0.443
Concentration	0.479

Polynomial Model: Differences in Time and Interaction of Time and Concentration

Effect	Best Model of Contrast[b]	Signficance[a]
Time	Cubic	0.011

Effect	ANOVA significance
Time[a]	<0.0001
Time*Concentration[a]	0.435
Concentration	0.362

Polynomial Model: Differences in Time and Interaction of Time and Concentration

Effect	Best Model of Contrast[b]	Signficance[a]
Time	Linear/Cubic	<0.0001

Effect	ANOVA significance
Time[a]	<0.0001
Time*Concentration[a]	0.242
Concentration	0.235

Polynomial Model: Differences in Time and Interaction of Time and Concentration

Effect	Best Model of Contrast[b]	Signficance[a]
Time	Cubic	<0.0001

Effect of Added Cytokine: Multivariate ANOVA

Time Point	1 Hour	24 Hours	72 Hours
Significance	<0.0001	<0.0001	<0.0001

Post Hoc Tests

IL10 1h	IL1β	IL6	TNF
None	1	1	1
IL1β		0.166	0.07
IL6			1

IL10 24h	IL1β	IL6	TNF
None	1	1	1
IL1β		0.029	0.095
IL6			1

IL10 72h	IL1β	IL6	TNF
None	1	1	1
IL1β		0.110	0.031
IL6			1

IL12p40

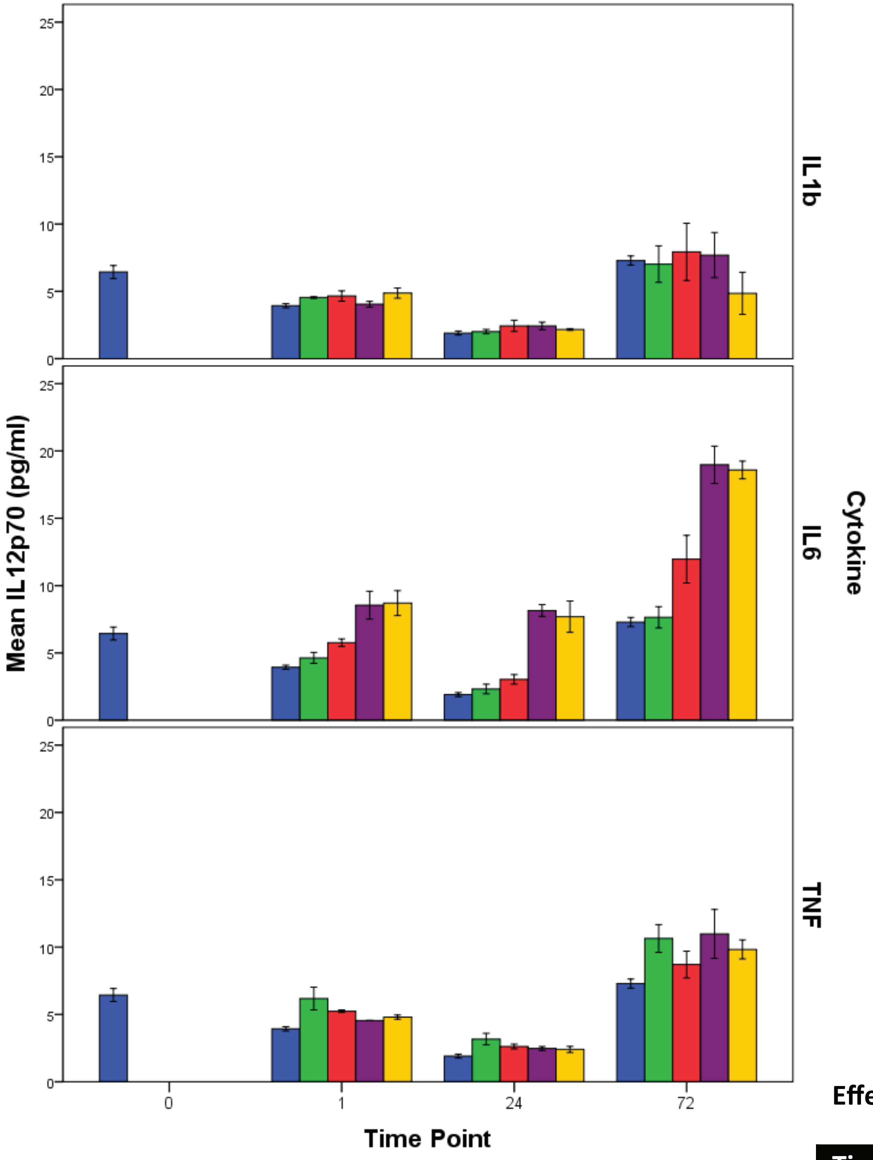

Two Way Mixed Anova

[a]Greenhouse-Geisser

Post Hoc Tests

[b]within well contrast with most stringent p-value (Linear vs Quadratic vs Cubic

IL1b

Effect	ANOVA significance
Time[a]	<0.0001
Time*Concentration[a]	0.540
Concentration	0.861

Polynomial Model: Differences in Time and Interaction of Time and Concentration

Effect	Best Model of Contrast[b]	Signficance[a]
Time	Quadratic/Cubic	<0.0001

IL6

Effect	ANOVA significance
Time[a]	<0.0001
Time*Concentration[a]	<0.0001
Concentration	<0.0001

Bonferroni: Differences Between Concentrations

Concentration	1	2	3	4
0	1	0.019	<0.0001	<0.0001
1		0.07	<0.0001	<0.0001
2			<0.0001	<0.0001
3				1

Polynomial Model: Differences in Time and Interaction of Time and Concentration

Effect	Best Model of Contrast[b]	Signficance[a]
Time	Linear/Quadratic/Cubic	<0.0001
Time*Concentration	Linear	<0.0001

TNF

Effect	ANOVA significance
Time[a]	<0.0001
Time*Concentration[a]	0.192
Concentration	0.132

Polynomial Model: Differences in Time and Interaction of Time and Concentration

Effect	Best Model of Contrast[b]	Signficance[a]
Time	Linear/Quadratic/Cubic	<0.0001

Effect of Added Cytokine: Multivariate ANOVA

Time Point	1 Hour	24 Hours	72 Hours
Significance	<0.0001	<0.0001	<0.0001

Post Hoc Tests

IL12p40 1h	IL1β	IL6	TNF
None	1	0.009	0.021
IL1β		0.001	1
IL6			0.021

IL12p40 24h	IL1β	IL6	TNF
None	1	0.022	1
IL1β		0.001	1
IL6			0.003

IL12p40 72h	IL1β	IL6	TNF
None	1	0.025	1
IL1β		<0.0001	0.214
IL6			0.035

IL12p70

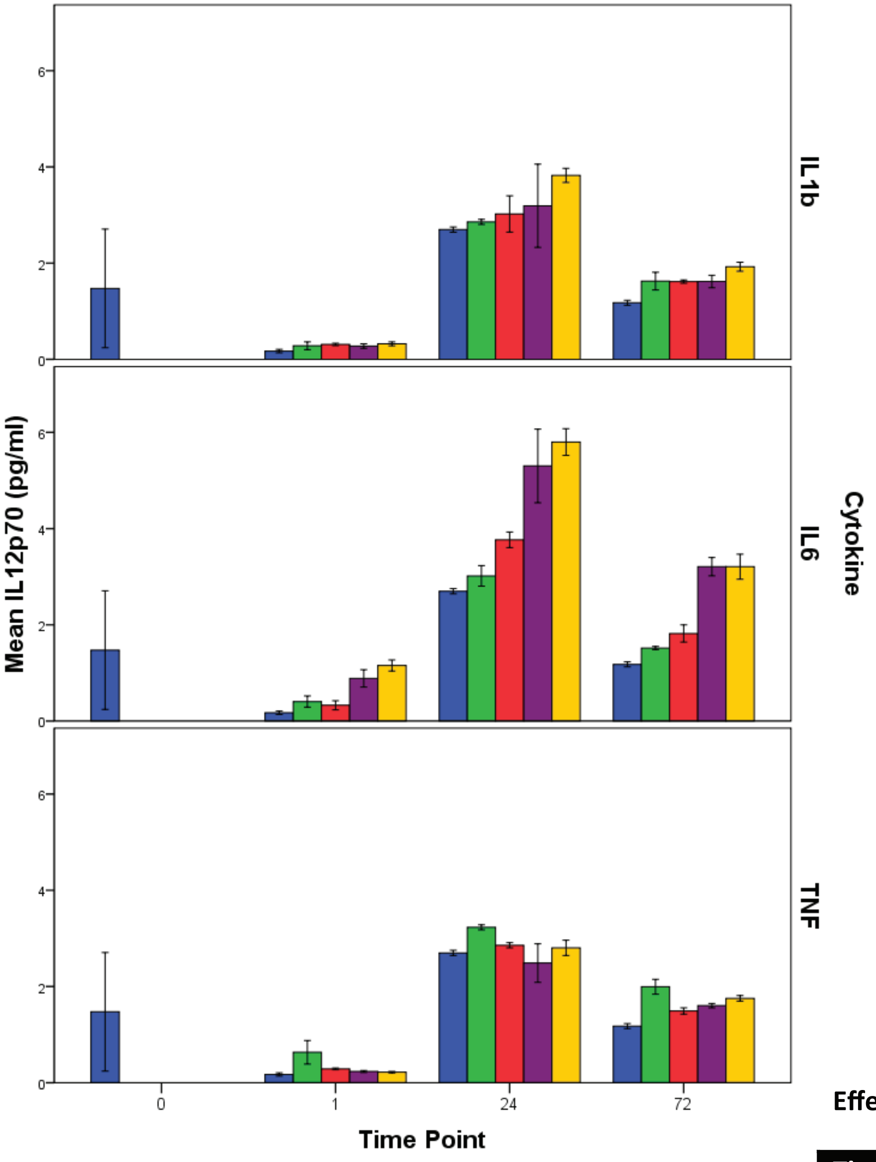

Two Way Mixed Anova

[a]Greenhouse-Geisser

Post Hoc Tests

[b]within well contrast with most stringent p-value (Linear vs Quadratic vs Cubic

IL1b

Effect	ANOVA significance
Time[a]	0.001
Time*Concentration[a]	0.989
Concentration	0.868

Polynomial Model: Differences in Time and Interaction of Time and Concentration

Effect	Best Model of Contrast[b]	Signficance[a]
Time	Cubic	<0.0001

IL6

Effect	ANOVA significance
Time[a]	<0.0001
Time*Concentration[a]	0.079
Concentration	0.031

Bonferroni: Differences Between Concentrations

Concentration	1	2	3	4
0	1	1	0.179	0.09
1		1	0.401	0.201
2			0.954	0.486
3				1

Polynomial Model: Differences in Time and Interaction of Time and Concentration

Effect	Best Model of Contrast[b]	Signficance[a]
Time	Cubic	<0.0001

TNF

Effect	ANOVA significance
Time[a]	0.004
Time*Concentration[a]	0.991
Concentration	0.885

Polynomial Model: Differences in Time and Interaction of Time and Concentration

Effect	Best Model of Contrast[b]	Signficance[a]
Time	Cubic	<0.0001

Effect of Added Cytokine: Multivariate ANOVA

Time Point	1 Hour	24 Hours	72 Hours
Significance	<0.0001	<0.0001	<0.0001

Post Hoc Tests

IL12p70 1h	IL1β	IL6	TNF
None	1	0.033	1
IL1β		0.007	1
IL6			0.02

IL12p70 24h	IL1β	IL6	TNF
None	1	0.027	1
IL1β		0.011	1
IL6			0.001

IL12p70 72h	IL1β	IL6	TNF
None	0.762	0.003	0.708
IL1β		0.007	1
IL6			0.009

IL13

Two Way Mixed Anova
[a]Greenhouse-Geisser

Post Hoc Tests
[b]within well contrast with most stringent p-value (Linear vs Quadratic vs Cubic

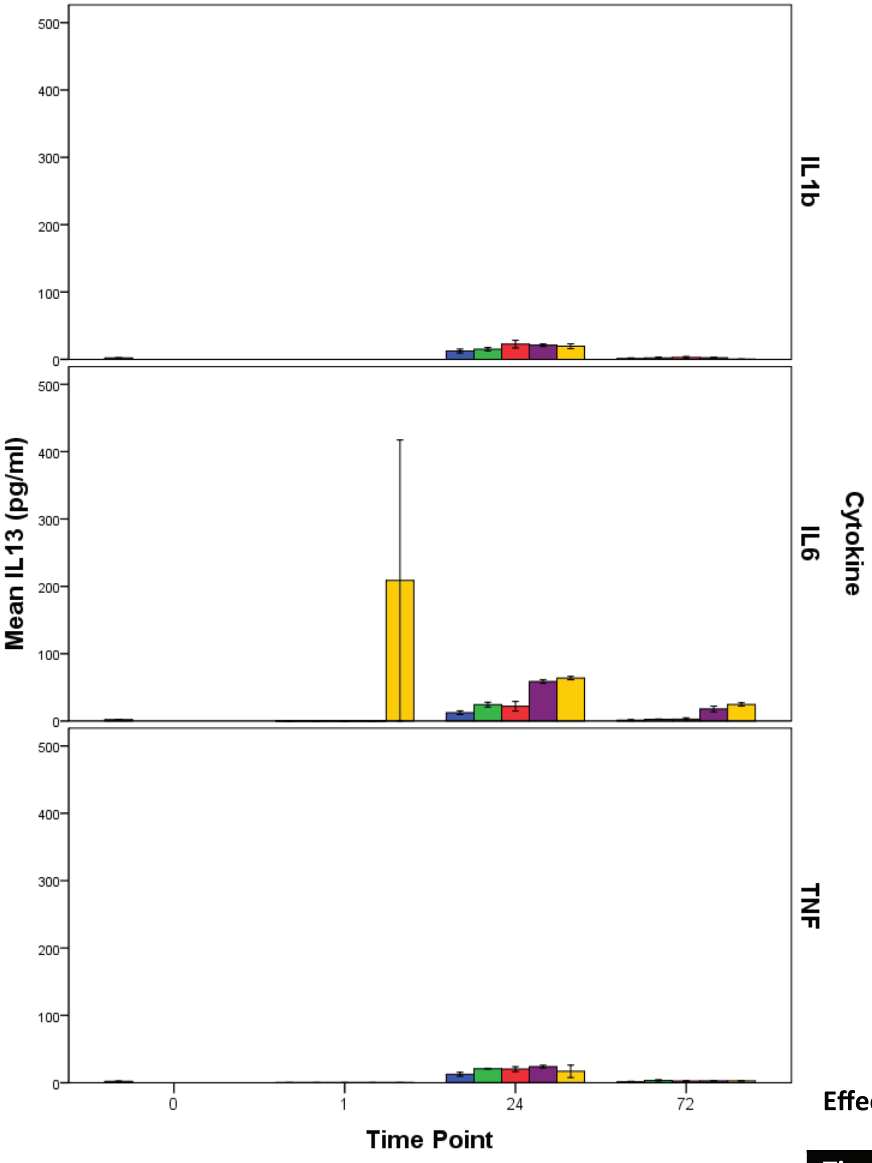

IL1b

Effect	ANOVA significance
Time[a]	<0.0001
Time*Concentration[a]	0.290
Concentration	0.392

Polynomial Model: Differences in Time and Interaction of Time and Concentration

Effect	Best Model of Contrast[b]	Signficance[a]
Time	Linear/Quadratic/Cubic	<0.0001

IL6

Effect	ANOVA significance
Time[a]	0.373
Time*Concentration[a]	0.506
Concentration	0.244

TNF

Effect	ANOVA significance
Time[a]	<0.0001
Time*Concentration[a]	0.597
Concentration	0.342

Polynomial Model: Differences in Time and Interaction of Time and Concentration

Effect	Best Model of Contrast[b]	Signficance[a]
Time	Linear/Quadratic/Cubic	<0.0001

Effect of Added Cytokine: Multivariate ANOVA

Time Point	1 Hour	24 Hours	72 Hours
Significance	0.536	<0.0001	<0.0001

Post Hoc Tests

IL13 24h	IL1β	IL6	TNF
None	1	0.007	1
IL1β		0.001	1
IL6			0.002

IL13 72h	IL1β	IL6	TNF
None	1	0.062	1
IL1β		0.002	1
IL6			0.005

IL15

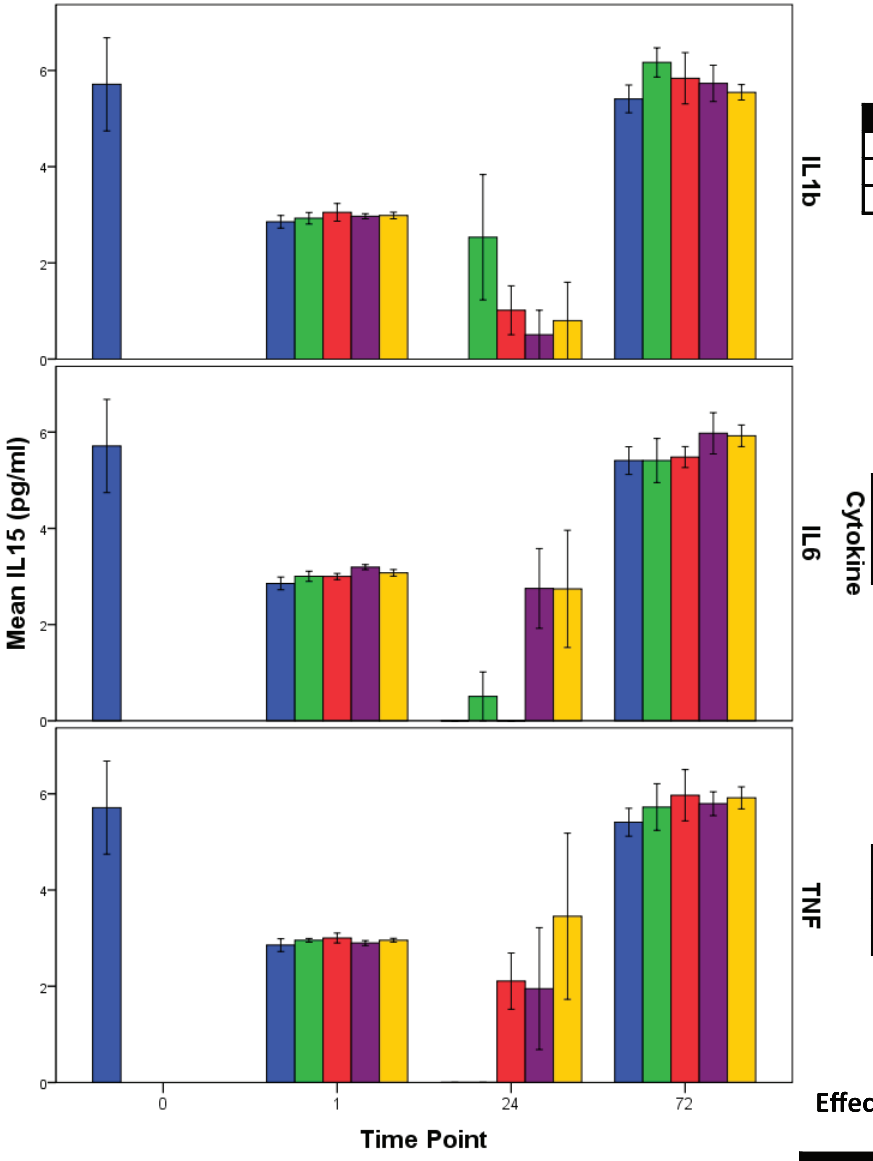

Two Way Mixed Anova

a Greenhouse-Geisser

IL1b

Effect	ANOVA significance
Time[a]	<0.0001
Time*Concentration[a]	0.777
Concentration	0.642

IL6

Effect	ANOVA significance
Time[a]	<0.0001
Time*Concentration[a]	0.349
Concentration	0.214

TNF

Effect	ANOVA significance
Time[a]	<0.0001
Time*Concentration[a]	0.483
Concentration	0.268

Post Hoc Tests

b within well contrast with most stringent p-value (Linear vs Quadratic vs Cubic

Polynomial Model: Differences in Time and Interaction of Time and Concentration

Effect	Best Model of Contrast[b]	Signficance[a]
Time	Quadratic	<0.0001

Polynomial Model: Differences in Time and Interaction of Time and Concentration

Effect	Best Model of Contrast[b]	Signficance[a]
Time	Quadratic/Cubic	<0.0001

Polynomial Model: Differences in Time and Interaction of Time and Concentration

Effect	Best Model of Contrast[b]	Signficance[a]
Time	Quadratic	<0.0001

Effect of Added Cytokine: Multivariate ANOVA

Time Point	1 Hour	24 Hours	72 Hours
Significance	<0.0001	<0.0001	<0.0001

Post Hoc Tests

IL15 1h	IL1β	IL6	TNF
None	1	0.210	1
IL1β		1	1
IL6			0.430

IL15 24h	IL1β	IL6	TNF
None	1	1	0.620
IL1β		1	1
IL6			1

IL15 72h	IL1β	IL6	TNF
None	1	1	1
IL1β		1	1
IL6			1

IL17

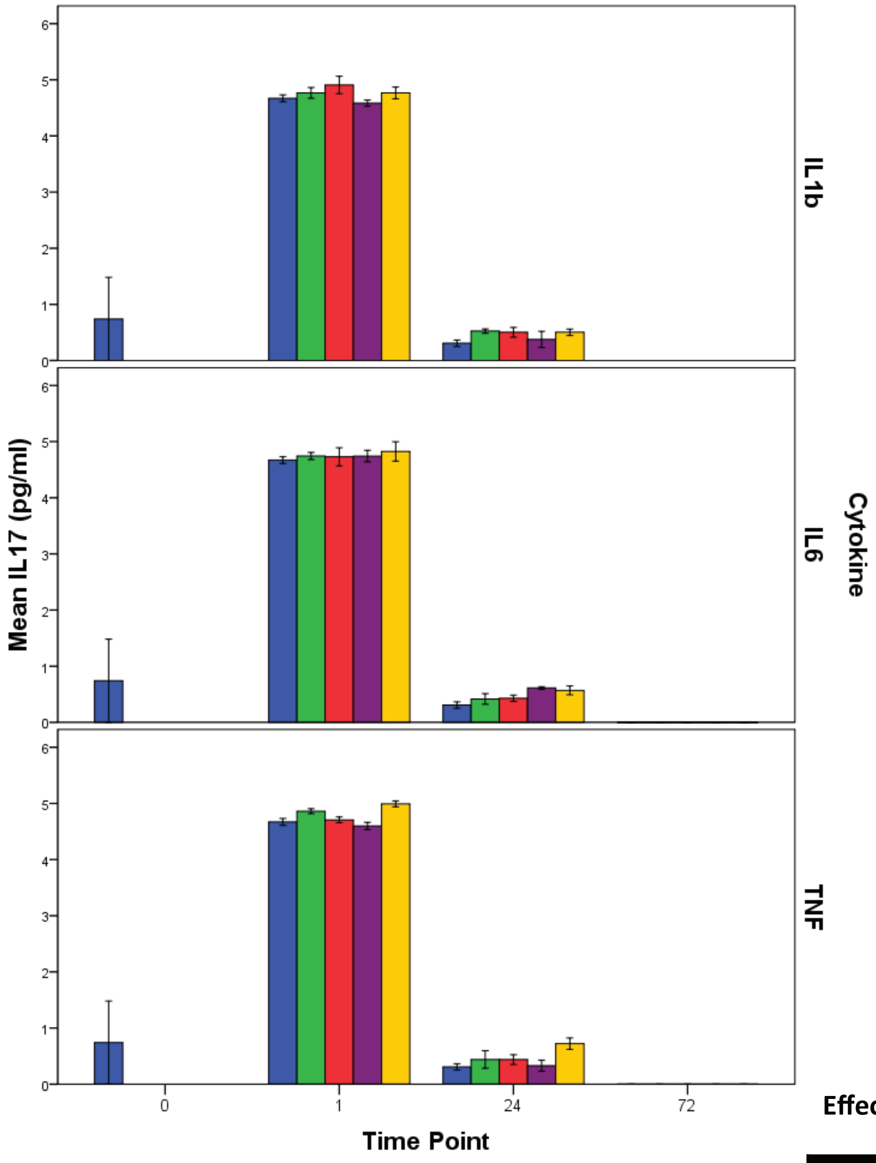

Two Way Mixed Anova

[a]Greenhouse-Geisser

Post Hoc Tests

[b]within well contrast with most stringent p-value (Linear vs Quadratic vs Cubic

Effect	ANOVA significance
Time[a]	<0.0001
Time*Concentration[a]	0.998
Concentration	0.990

Polynomial Model: Differences in Time and Interaction of Time and Concentration

Effect	Best Model of Contrast[b]	Signficance[a]
Time	Linear/Quadratic/Cubic	<0.0001

Effect	ANOVA significance
Time[a]	<0.0001
Time*Concentration[a]	0.999
Concentration	0.991

Polynomial Model: Differences in Time and Interaction of Time and Concentration

Effect	Best Model of Contrast[b]	Signficance[a]
Time	Linear/Quadratic/Cubic	<0.0001

Effect	ANOVA significance
Time[a]	<0.0001
Time*Concentration[a]	0.992
Concentration	0.949

Polynomial Model: Differences in Time and Interaction of Time and Concentration

Effect	Best Model of Contrast[b]	Signficance[a]
Time	Linear/Quadratic/Cubic	<0.0001

Effect of Added Cytokine: Multivariate ANOVA

Time Point	1 Hour	24 Hours
Significance[a]	<0.0001	<0.0001

Post Hoc Tests

IL17 1h	IL1β	IL6	TNF
None	1	1	1
IL1β		1	1
IL6			1

IL17 24h	IL1β	IL6	TNF
None	0.816	0.488	0.742
IL1β		1	1
IL6			1

IL1a

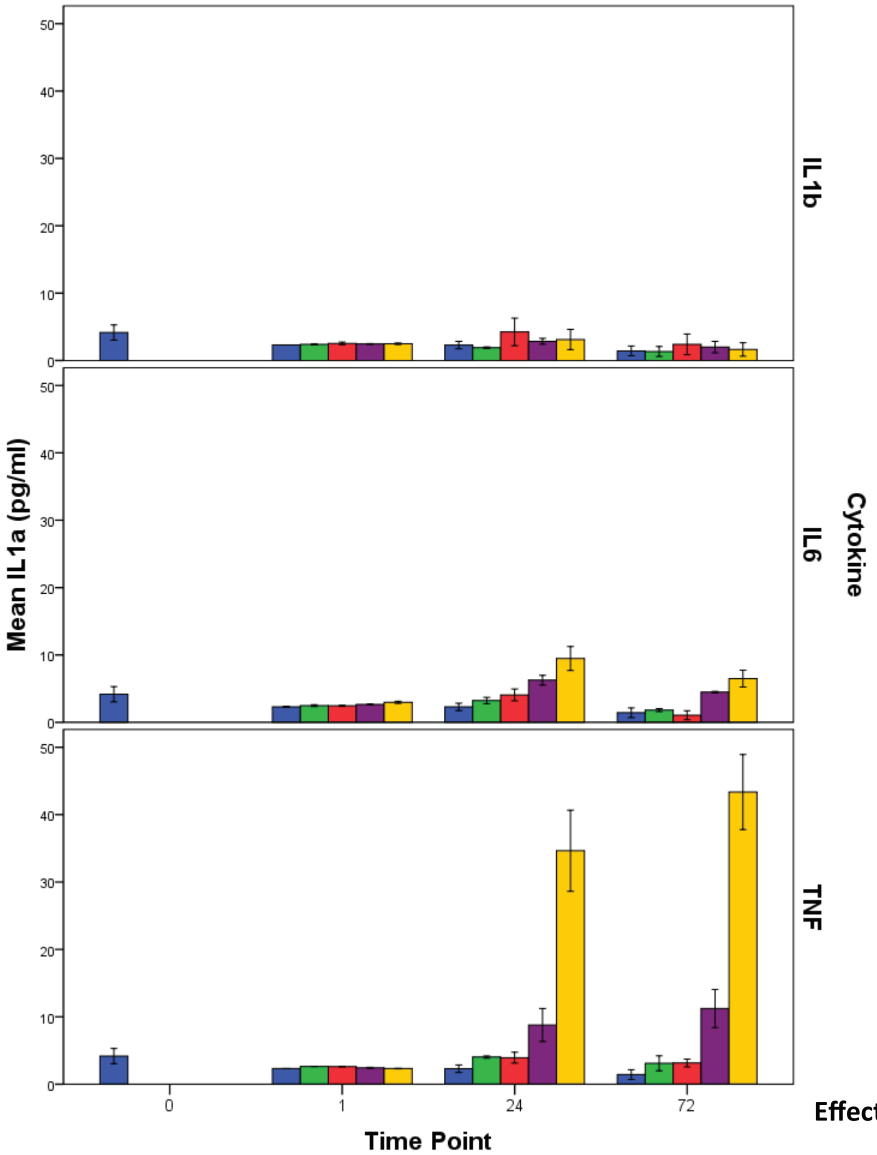

Two Way Mixed Anova

[a]Greenhouse-Geisser

Post Hoc Tests

[b]within well contrast with most stringent p-value (Linear vs Quadratic vs Cubic

IL1b

Effect	ANOVA significance
Time[a]	0.001
Time*Concentration[a]	0.974
Concentration	0.900

Polynomial Model: Differences in Time and Interaction of Time and Concentration

Effect	Best Model of Contrast[b]	Signficance[a]
Time	Linear	0.005

IL6

Effect	ANOVA significance
Time[a]	0.001
Time*Concentration[a]	0.008
Concentration	0.005

Bonferroni: Differences Between Concentrations

Concentration	1	2	3	4
0	1	1	0.261	0.01
1		1	0.651	0.024
2			0.662	0.024
3				0.771

Polynomial Model: Differences in Time and Interaction of Time and Concentration

Effect	Best Model of Contrast[b]	Signficance[a]
Time	Cubic	<0.0001
Time*Concentration	Linear	0.002

TNF

Effect	ANOVA significance
Time[a]	<0.0001
Time*Concentration[a]	<0.0001
Concentration	<0.0001

Bonferroni: Differences Between Concentrations

Concentration	1	2	3	4
0	1	1	0.166	<0.0001
1		1	0.507	<0.0001
2			0.495	<0.0001
3				<0.0001

Polynomial Model: Differences in Time and Interaction of Time and Concentration

Effect	Best Model of Contrast[b]	Signficance[a]
Time	LInear	<0.0001
Time*Concentration	Linear	<0.0001

Effect of Added Cytokine: Multivariate ANOVA

Time Point	1 Hour	24 Hours	72 Hours
Significance	<0.0001	<0.0001	<0.0001

Post Hoc Tests

IL1a 1h	IL1β	IL6	TNF
None	1	0.089	0.98
IL1β		.243	1
IL6			0.484

IL1a 24h	IL1β	IL6	TNF
None	1	1	0.249
IL1β		1	0.035
IL6			0.249

IL1a 72h	IL1β	IL6	TNF
None	1	1	0.262
IL1β		1	0.017
IL6			0.047

IL1b

Two Way Mixed Anova
[a]Greenhouse-Geisser

Post Hoc Tests
[b]within well contrast with most stringent p-value (Linear vs Quadratic vs Cubic

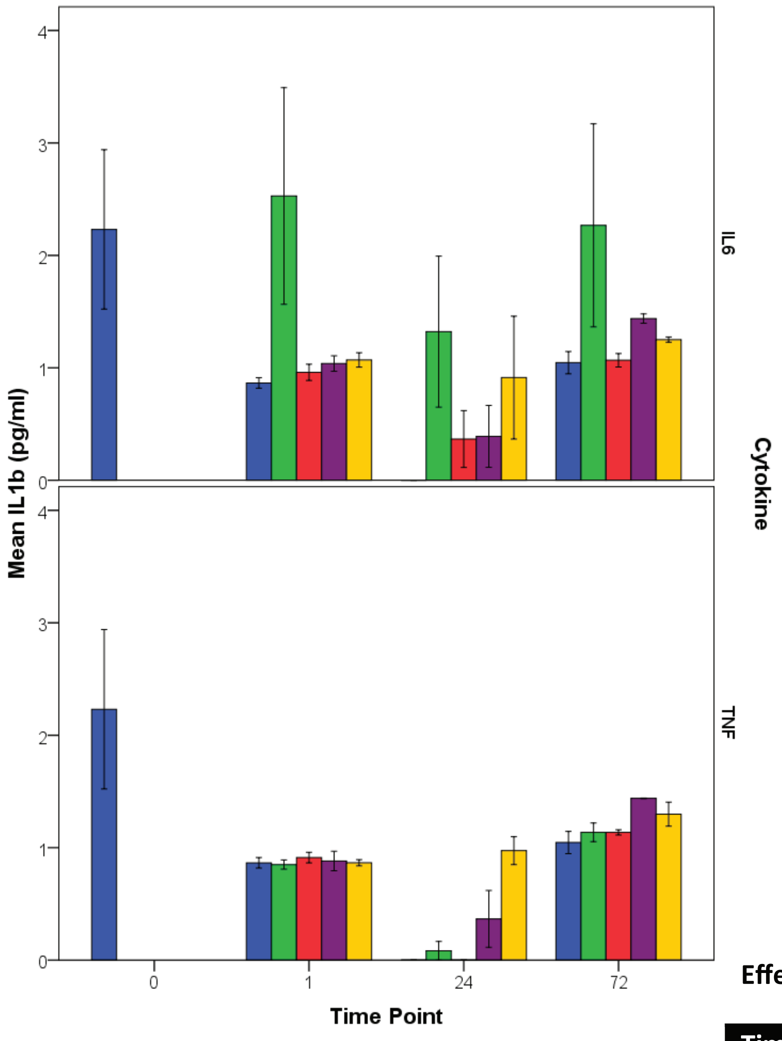

Effect	ANOVA significance
Time[a]	0.001
Time*Concentration[a]	0.763
Concentration	0.279

[a]Greenhouse-Geisser

Polynomial Model: Differences in Time and Interaction of Time and Concentration

Effect	Best Model of Contrast[b]	Signficance[a]
Time	Quadratic	0.001

[b]Within-well contrast with most stringent p-value (Linear vs Quadratic vs Cubic)

Effect	ANOVA significance
Time[a]	<0.0001
Time*Concentration[a]	0.872
Concentration	0.715

[a]Greenhouse-Geisser

Polynomial Model: Differences in Time and Interaction of Time and Concentration

Effect	Best Model of Contrast[b]	Signficance[a]
Time	Quadratic	<0.0001

[b]Within-well contrast with most stringent p-value (Linear vs Quadratic vs Cubic)

Effect of Added Cytokine: Multivariate ANOVA

Time Point	1 Hour	24 Hours	72 Hours
Significance	<0.0001	0.002	<0.0001

Post Hoc Tests

IL1b 1h	IL6	TNF
None	0.693	1
IL6		0.209

IL1b 24h	IL6	TNF
None	0.233	1
IL6		0.419

IL1b 72h	IL6	TNF
None	0.671	1
IL6		0.861

IL1ra

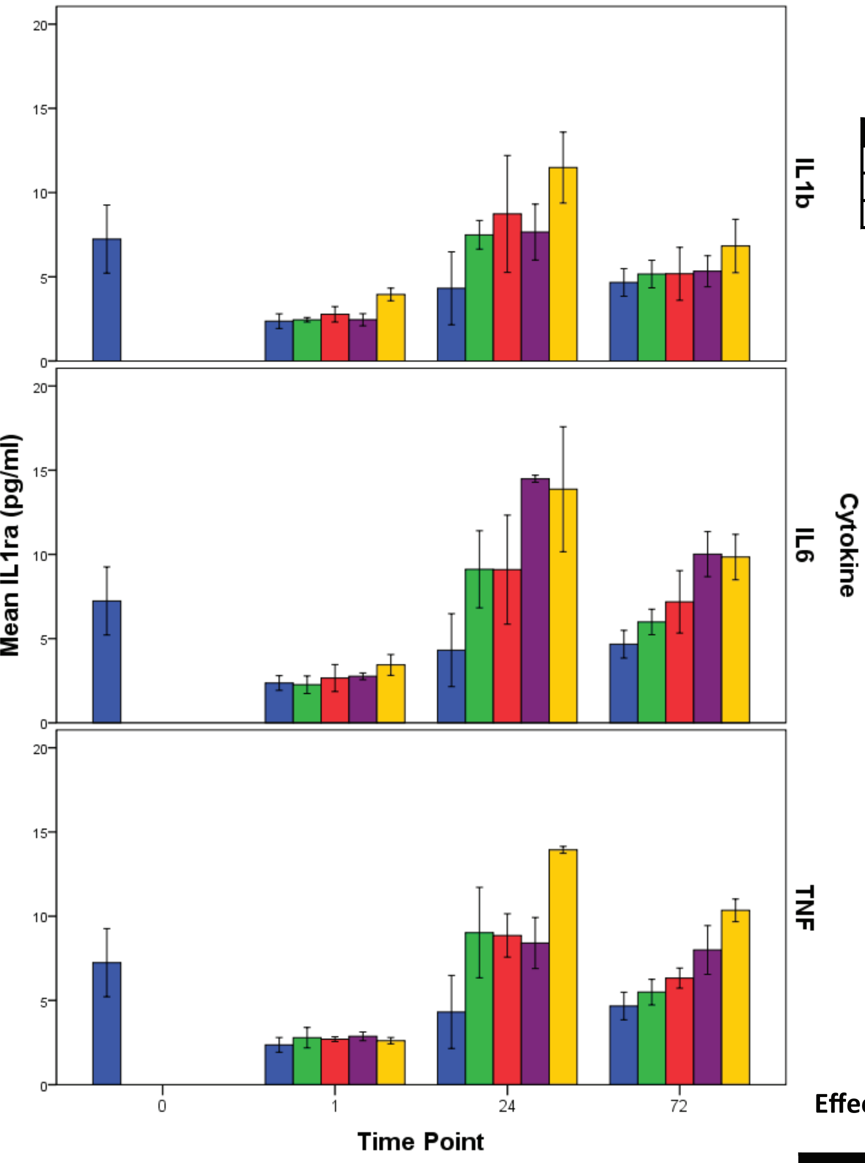

Two Way Mixed Anova

[a]Greenhouse-Geisser

Post Hoc Tests

[b]within well contrast with most stringent p-value (Linear vs Quadratic vs Cubic

IL1b

Effect	ANOVA significance
Time[a]	<0.0001
Time*Concentration[a]	0.659
Concentration	0.578

Polynomial Model: Differences in Time and Interaction of Time and Concentration

Effect	Best Model of Contrast[b]	Signficance[a]
Time	Cubic	<0.0001

IL6

Effect	ANOVA significance
Time[a]	<0.0001
Time*Concentration[a]	0.289
Concentration	0.102

Polynomial Model: Differences in Time and Interaction of Time and Concentration

Effect	Best Model of Contrast[b]	Signficance[a]
Time	Cubic	<0.0001

TNF

Effect	ANOVA significance
Time[a]	<0.0001
Time*Concentration[a]	0.094
Concentration	0.167

Polynomial Model: Differences in Time and Interaction of Time and Concentration

Effect	Best Model of Contrast[b]	Signficance[a]
Time	Cubic	<0.0001

Effect of Added Cytokine: Multivariate ANOVA

Time Point	1 Hour	24 Hours	72 Hours
Significance	<0.0001	<0.0001	<0.0001

Post Hoc Tests

IL1ra 1h	IL1β	IL6	TNF
None	1	1	1
IL1β		1	1
IL6			1

IL1ra 24h	IL1β	IL6	TNF
None	0.534	0.046	0.198
IL1β		0.573	1
IL6			1

IL1ra 72h	IL1β	IL6	TNF
None	1	0.14	0.396
IL1β		0.056	0.319
IL6			1

IL2

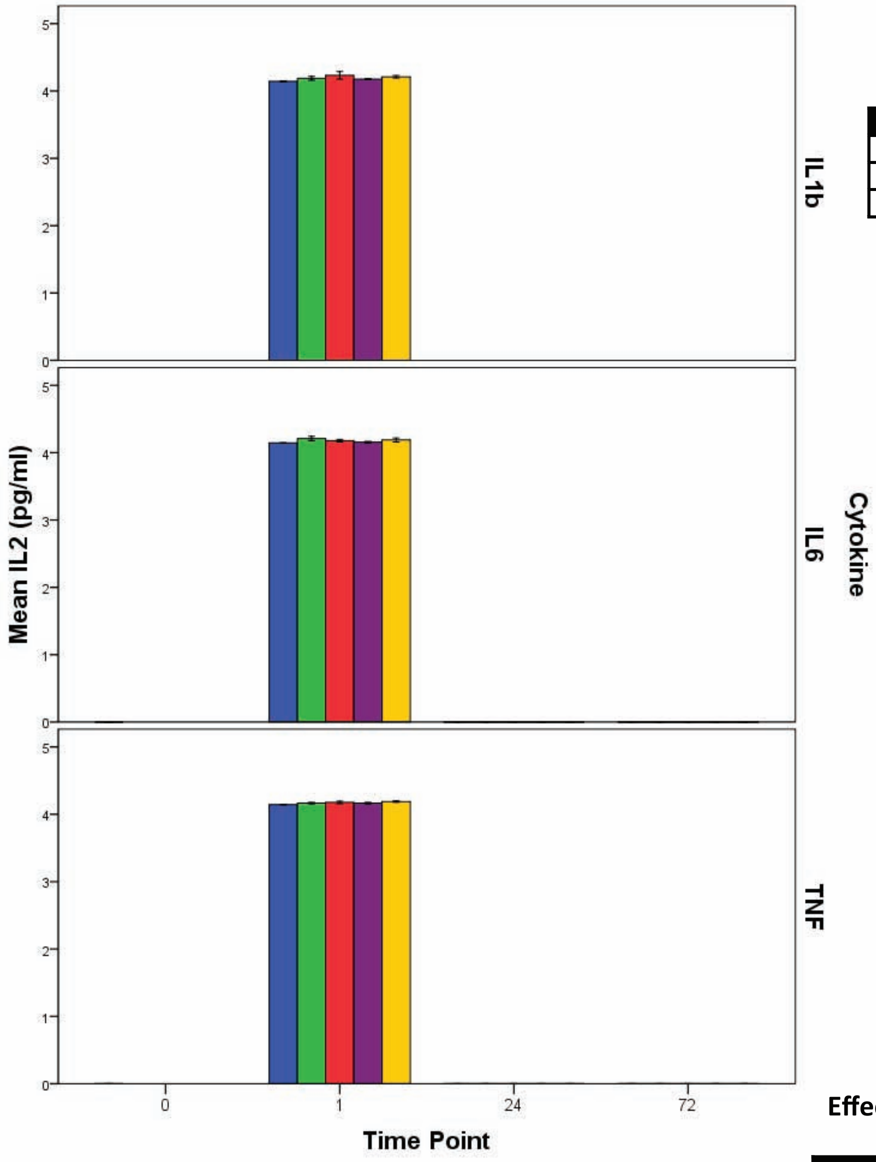

Two Way Mixed Anova

[a]Greenhouse-Geisser

Post Hoc Tests

[b]within well contrast with most stringent p-value (Linear vs Quadratic vs Cubic

Effect	ANOVA significance
Time[a]	<0.0001
Time*Concentration[a]	0.330
Concentration	0.330

Post Hoc Tests

Polynomial Model: Differences in Time and Interaction of Time and Concentration

Effect	Best Model of Contrast[b]	Signficance[a]
Time	Linear/Quadratic/Cubic	<0.0001

Effect	ANOVA significance
Time[a]	<0.0001
Time*Concentration[a]	0.295
Concentration	0.295

Polynomial Model: Differences in Time and Interaction of Time and Concentration

Effect	Best Model of Contrast[b]	Signficance[a]
Time	Linear/Quadratic/Cubic	<0.0001

Effect	ANOVA significance
Time[a]	<0.0001
Time*Concentration[a]	0.199
Concentration	0.199

Polynomial Model: Differences in Time and Interaction of Time and Concentration

Effect	Best Model of Contrast[b]	Signficance[a]
Time	Linear/Quadratic/Cubic	<0.0001

Effect of Added Cytokine: Multivariate ANOVA

Time Point	1 Hour
Significance	<0.0001

Post Hoc Tests

IL2 1h	IL1β	IL6	TNF
None	0.186	0.860	1
IL1β		1	0.593
IL6			1

IL3

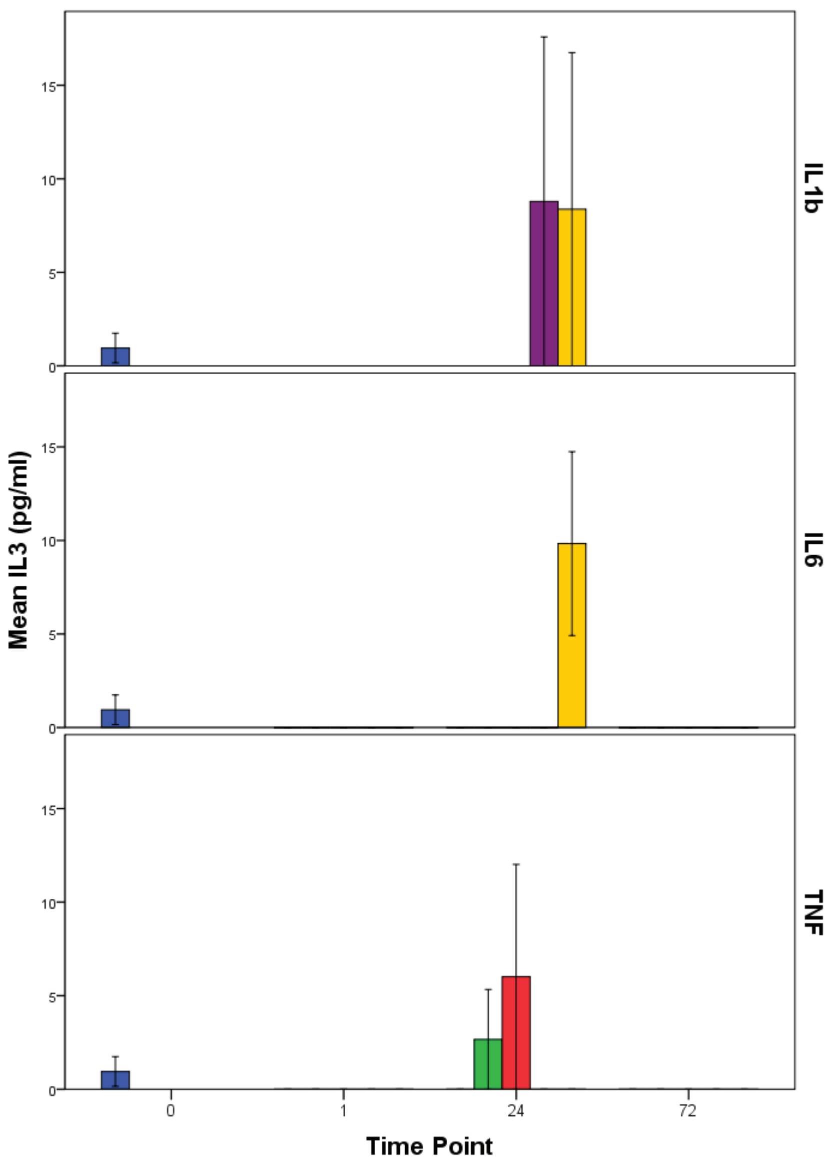

Two Way Mixed Anova

aGreenhouse-Geisser

Effect	ANOVA significance
Time[a]	0.223
Time*Concentration[a]	0.606
Concentration	0.544

Effect	ANOVA significance
Time[a]	0.085
Time*Concentration[a]	0.029
Concentration	0.065

Effect	ANOVA significance
Time[a]	0.249
Time*Concentration[a]	0.589
Concentration	0.566

Post Hoc Tests

bwithin well contrast with most stringent p-value (Linear vs Quadratic vs Cubic

Polynomial Model: Differences in Time and Interaction of Time and Concentration

Effect	Best Model of Contrast[b]	Signficance[a]
Time*Concentration	Quadratic	0.033

Effect of Added Cytokine: Multivariate ANOVA

Time Point	72 Hours
Significance	0.172

Post Hoc Tests

IL4

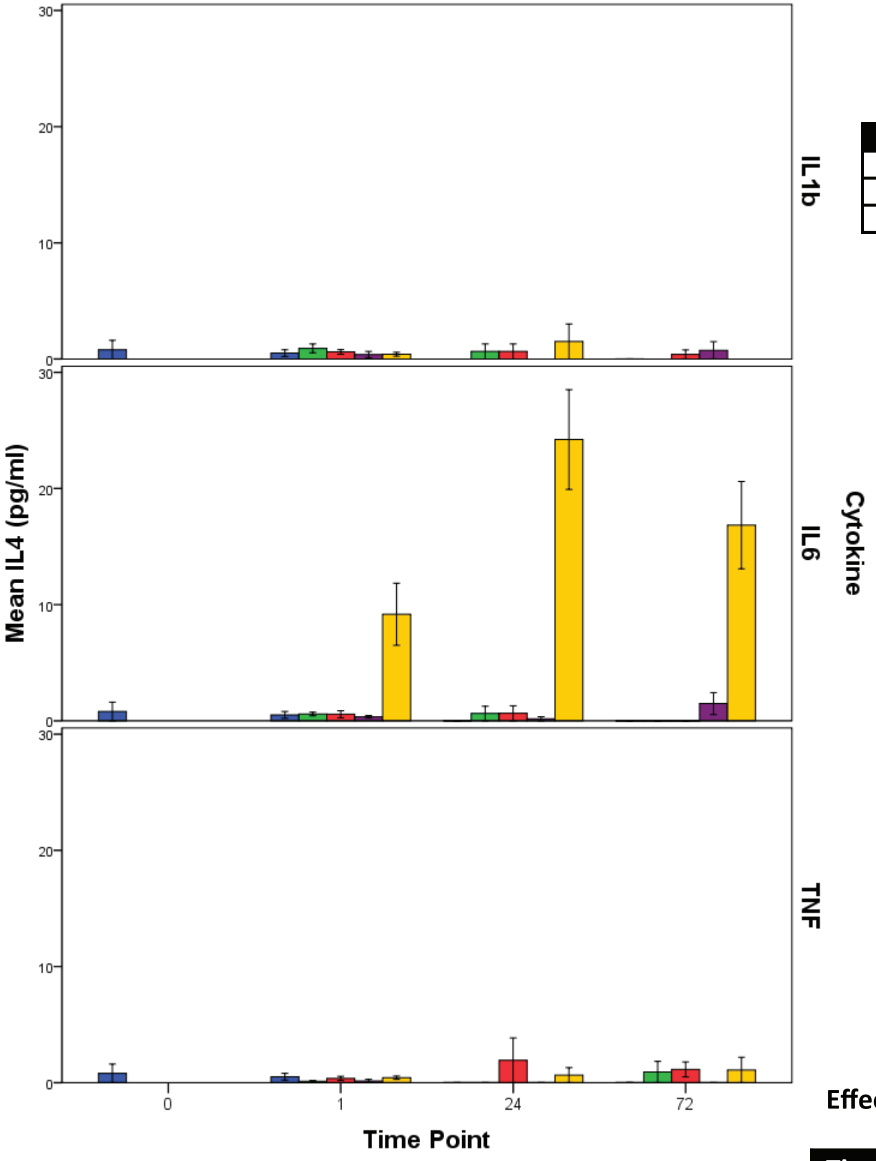

Two Way Mixed Anova

[a]Greenhouse-Geisser

IL1b

Effect	ANOVA significance
Time[a]	0.462
Time*Concentration[a]	0.874
Concentration	0.953

IL6

Effect	ANOVA significance
Time[a]	0.023
Time*Concentration[a]	0.002
Concentration	<0.0001

TNF

Effect	ANOVA significance
Time[a]	0.660
Time*Concentration[a]	0.906
Concentration	0.341

Post Hoc Tests

[b]within well contrast with most stringent p-value (Linear vs Quadratic vs Cubic

Bonferroni: Differences Between Concentrations

Concentration	1	2	3	4
0	1	1	1	<0.0001
1		1	1	<0.0001
2			1	<0.0001
3				<0.0001

Polynomial Model: Differences in Time and Interaction of Time and Concentration

Effect	Best Model of Contrast[b]	Signficance[a]
Time	Linear	<0.0001
Time*Concentration	Linear	<0.0001

Effect of Added Cytokine: Multivariate ANOVA

Time Point	1 Hour	24 Hours	72 Hours
Significance	0.013	0.029	0.025

Post Hoc Tests

IL4 1h	IL1β	IL6	TNF
None	1	1	1
IL1β		0.283	1
IL6			0.139

IL4 24h	IL1β	IL6	TNF
None	1	0.779	1
IL1β		0.214	1
IL6			0.204

IL4 72h	IL1β	IL6	TNF
None	1	0.758	1
IL1β		0.156	1
IL6			0.285

IL5

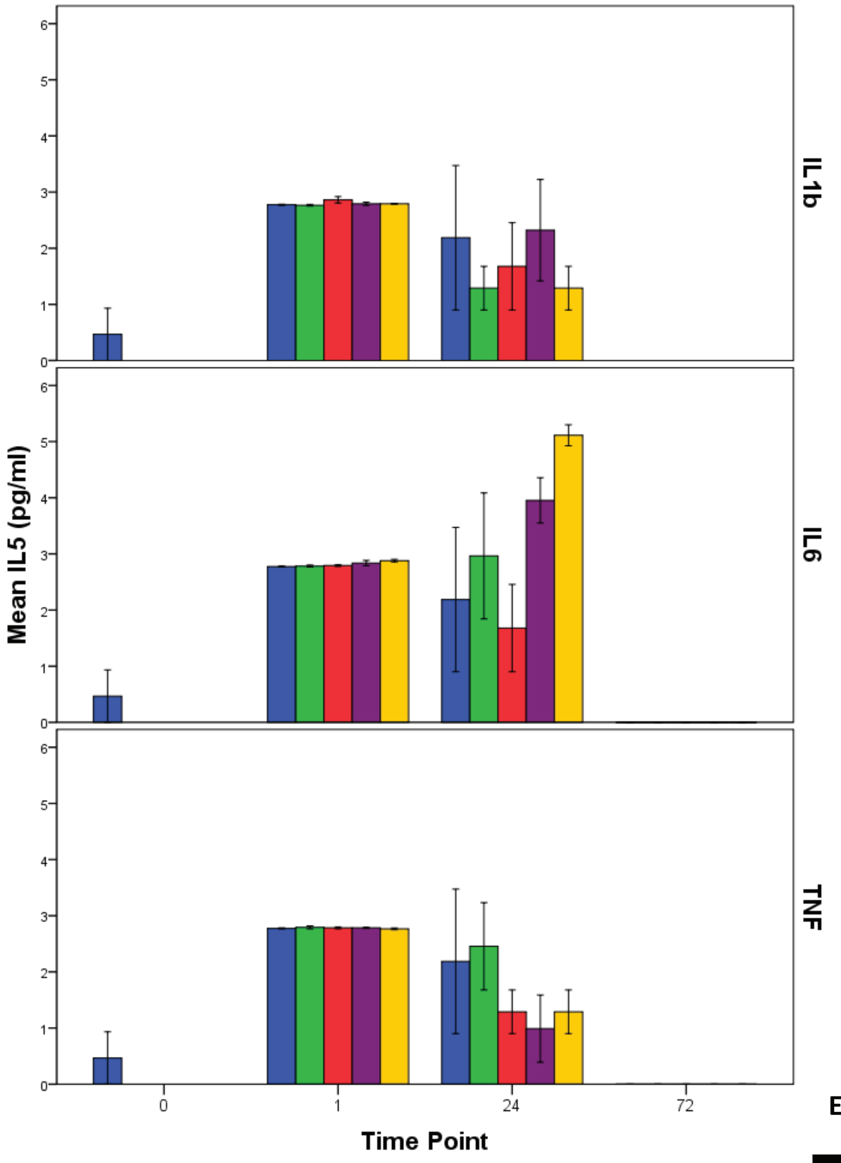

Two Way Mixed Anova
[a]Greenhouse-Geisser

Post Hoc Tests
[b]within well contrast with most stringent p-value (Linear vs Quadratic vs Cubic

IL1b:

Effect	ANOVA significance
Time[a]	<0.0001
Time*Concentration[a]	0.940
Concentration	0.819

Polynomial Model: Differences in Time and Interaction of Time and Concentration

Effect	Best Model of Contrast[b]	Signficance[a]
Time	Quadratic	<0.0001

IL6:

Effect	ANOVA significance
Time[a]	<0.0001
Time*Concentration[a]	0.133
Concentration	0.172

Polynomial Model: Differences in Time and Interaction of Time and Concentration

Effect	Best Model of Contrast[b]	Signficance[a]
Time	Quadratic	<0.0001

TNF:

Effect	ANOVA significance
Time[a]	<0.0001
Time*Concentration[a]	0.807
Concentration	0.614

Polynomial Model: Differences in Time and Interaction of Time and Concentration

Effect	Best Model of Contrast[b]	Signficance[a]
Time	Quadratic	<0.0001

Effect of Added Cytokine: Multivariate ANOVA

Time Point	1 Hour	24 Hours
Significance	<0.0001	<0.0001

Post Hoc Tests

IL5 1h	IL1β	IL6	TNF
None	1	0.881	1
IL1β		1	1
IL6			0.302

IL5 24h	IL1β	IL6	TNF
None	1	1	1
IL1β		0.018	1
IL6			0.009

IL6

Two Way Mixed Anova
[a]Greenhouse-Geisser

Post Hoc Tests
[b]within well contrast with most stringent p-value (Linear vs Quadratic vs Cubic

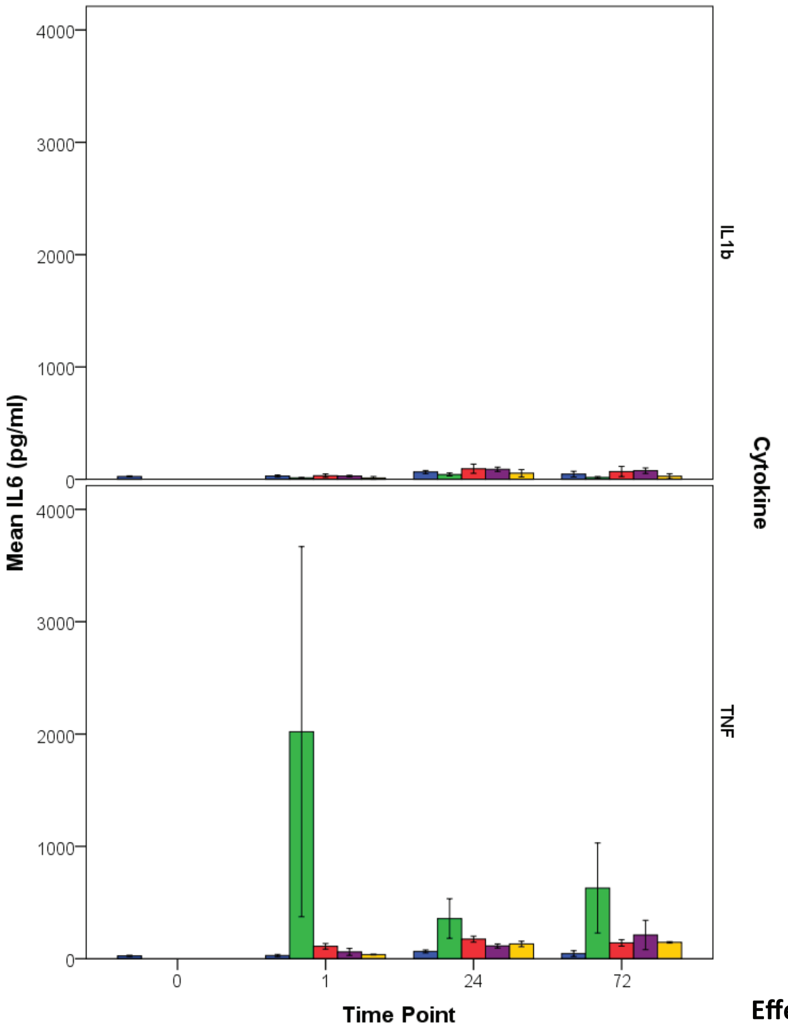

Effect	ANOVA significance
Time[a]	0.005
Time*Concentration[a]	0.602
Concentration	0.553

[a]Greenhouse-Geisser

Polynomial Model: Differences in Time and Interaction of Time and Concentration

Effect	Best Model of Contrast[b]	Signficance[a]
Time	Cubic	<0.0001

[b]Within-well contrast with most stringent p-value (Linear vs Quadratic vs Cubic)

Effect	ANOVA significance
Time[a]	0.263
Time*Concentration[a]	0.301
Concentration	0.284

[a]Greenhouse-Geisser

Effect of Added Cytokine: Multivariate ANOVA

Time Point	1 Hour	24 Hours	72 Hours
Significance	0.331	<0.0001	0.008

Post Hoc Tests

IL6 24h	IL1β	TNF
None	1	0.309
IL1β		0.05

IL6 72h	IL1β	TNF
None	1	0.509
IL1β		0.107

IL7

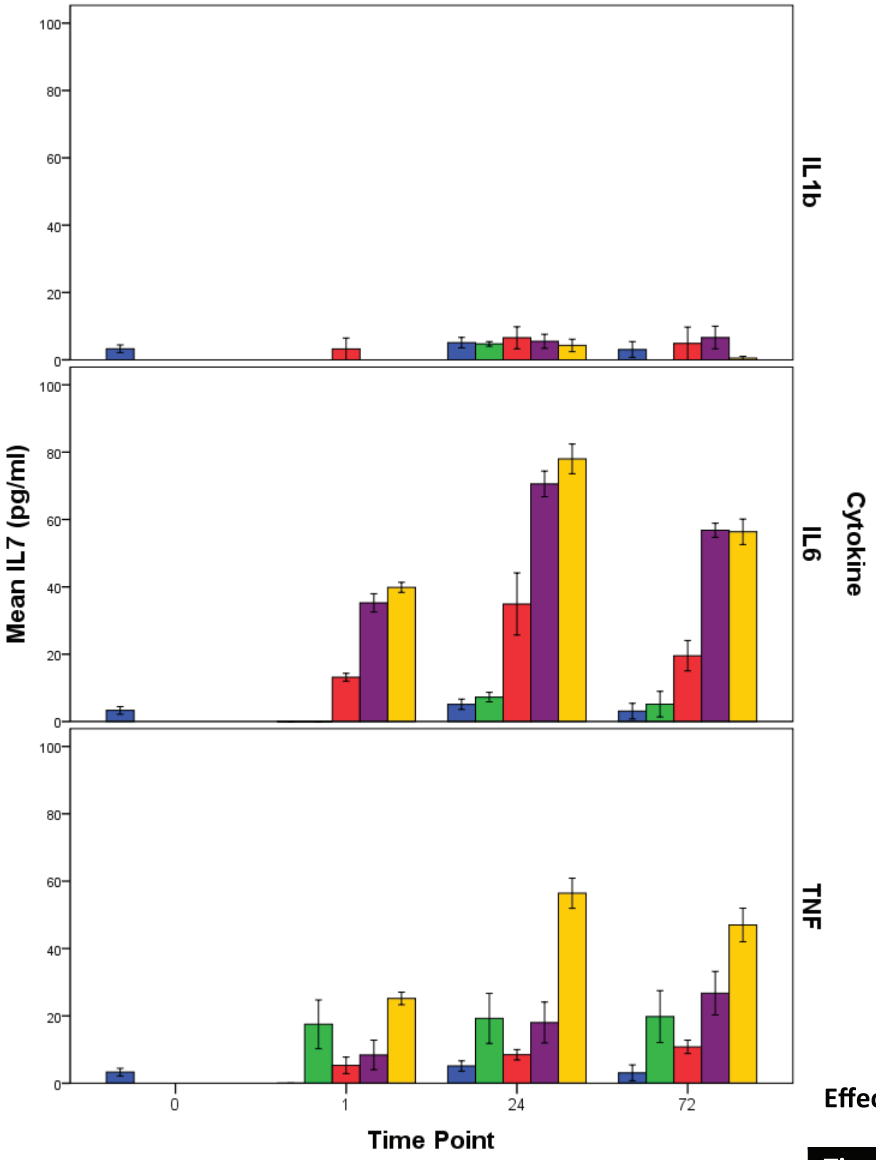

Two Way Mixed Anova

[a]Greenhouse-Geisser

Post Hoc Tests

[b]within well contrast with most stringent p-value (Linear vs Quadratic vs Cubic

IL1b

Effect	ANOVA significance
Time[a]	0.007
Time*Concentration[a]	0.560
Concentration	0.699

Polynomial Model: Differences in Time and Interaction of Time and Concentration

Effect	Best Model of Contrast[b]	Signficance[a]
Time	Cubic	<0.0001

IL6

Effect	ANOVA significance
Time[a]	<0.0001
Time*Concentration[a]	<0.0001
Concentration	<0.0001

Bonferroni: Differences Between Concentrations

Concentration	1	2	3	4
0	1	0.005	<0.0001	<0.0001
1		0.008	<0.0001	<0.0001
2			<0.0001	<0.0001
3				1

Polynomial Model: Differences in Time and Interaction of Time and Concentration

Effect	Best Model of Contrast[b]	Signficance[a]
Time	Linear/Quadratic/Cubic	<0.0001
Time*Concentration	Linear/Quadratic	<0.0001

TNF

Effect	ANOVA significance
Time[a]	<0.0001
Time*Concentration[a]	<0.0001
Concentration	<0.0001

Bonferroni: Differences Between Concentrations

Concentration	1	2	3	4
0	0.158	1	0.223	<0.0001
1		0.839	1	0.015
2			1	0.001
3				0.011

Polynomial Model: Differences in Time and Interaction of Time and Concentration

Effect	Best Model of Contrast[b]	Signficance[a]
Time	Linear	<0.0001
Time*Concentration	Linear	<0.0001

Effect of Added Cytokine: Multivariate ANOVA

Time Point	1 Hour	24 Hours	72 Hours
Significance	<0.0001	<0.0001	<0.0001

Post Hoc Tests

IL7 1h	IL1β	IL6	TNF
None	1	0.03	0.381
IL1β		<0.0001	0.043
IL6			0.572

IL7 24h	IL1β	IL6	TNF
None	1	0.02	0.835
IL1β		<0.0001	0.140
IL6			0.082

IL7 72h	IL1β	IL6	TNF
None	1	0.037	0.239
IL1β		<0.0001	0.011
IL6			1

IL8

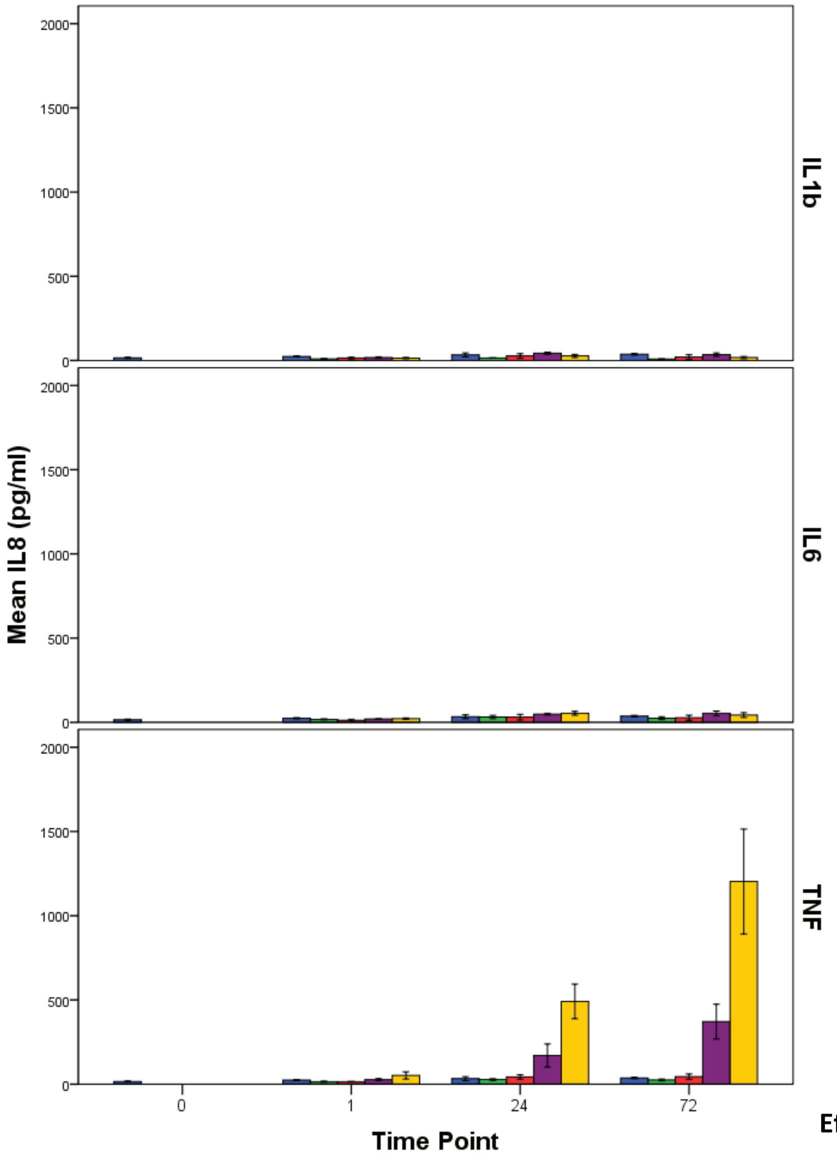

Two Way Mixed Anova

[a]Greenhouse-Geisser

IL1b

Effect	ANOVA significance
Time[a]	0.004
Time*Concentration[a]	0.223
Concentration	0.292

IL6

Effect	ANOVA significance
Time[a]	<0.0001
Time*Concentration[a]	0.458
Concentration	0.547

TNF

Effect	ANOVA significance
Time[a]	<0.0001
Time*Concentration[a]	0.001
Concentration	0.001

Post Hoc Tests

[b]within well contrast with most stringent p-value (Linear vs Quadratic vs Cubic

IL1b

Polynomial Model: Differences in Time and Interaction of Time and Concentration

Effect	Best Model of Contrast[b]	Signficance[a]
Time	Cubic	0.001

IL6

Polynomial Model: Differences in Time and Interaction of Time and Concentration

Effect	Best Model of Contrast[b]	Signficance[a]
Time	Cubic	<0.0001

TNF

Bonferroni: Differences Between Concentrations

Concentration	1	2	3	4
0	1	1	1	0.002
1		1	1	0.002
2			1	0.002
3				0.023

Polynomial Model: Differences in Time and Interaction of Time and Concentration

Effect	Best Model of Contrast[b]	Signficance[a]
Time	Linear	0.001
Time*Concentration	Linear	0.001

Effect of Added Cytokine: Multivariate ANOVA

Time Point	1 Hour	24 Hours	72 Hours
Significance	<0.0001	<0.0001	0.002

Post Hoc Tests

IL8 1h	IL1β	IL6	TNF
None	1	1	1
IL1β		1	0.165
IL6			0.668

IL8 24h	IL1β	IL6	TNF
None	1	1	0.391
IL1β		1	0.022
IL6			0.044

IL8 72h	IL1β	IL6	TNF
None	1	1	0.426
IL1β		1	0.024
IL6			0.035

IL9

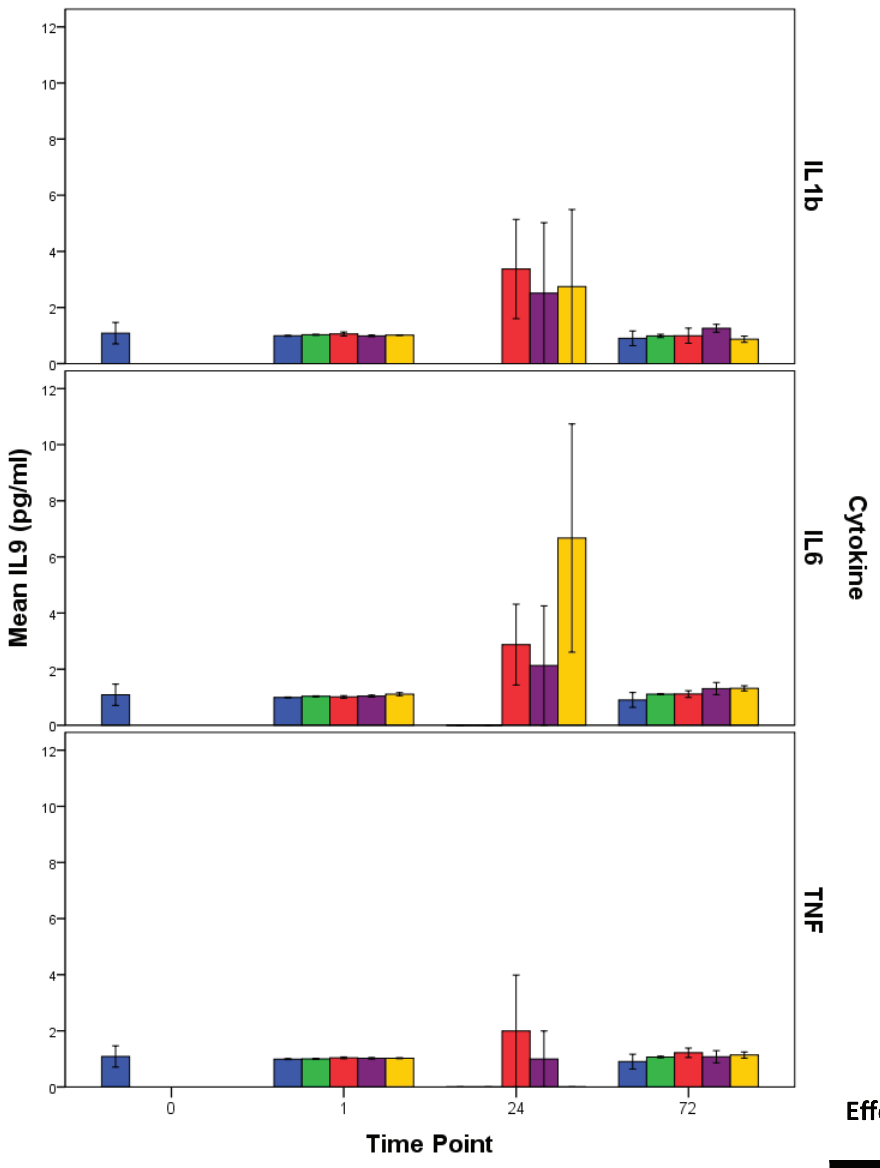

Two Way Mixed Anova

[a]Greenhouse-Geisser

Post Hoc Tests

[b]within well contrast with most stringent p-value (Linear vs Quadratic vs Cubic

Effect	ANOVA significance
Time[a]	0.437
Time*Concentration[a]	0.603
Concentration	0.583

Effect	ANOVA significance
Time[a]	0.236
Time*Concentration[a]	0.275
Concentration	0.192

Effect	ANOVA significance
Time[a]	0.374
Time*Concentration[a]	0.669
Concentration	0.536

Effect of Added Cytokine: Multivariate ANOVA

Time Point	1 Hour	24 Hours	72 Hours
Significance	<0.0001	0.01	<0.0001

Post Hoc Tests

IL9 1h	IL1β	IL6	TNF
None	1	0.873	1
IL1β		1	1
IL6			1

IL9 24h	IL1β	IL6	TNF
None	1	1	1
IL1β		1	1
IL6			0.661

IL9 72h	IL1β	IL6	TNF
None	1	0.464	1
IL1β		0.574	1
IL6			1

IP10

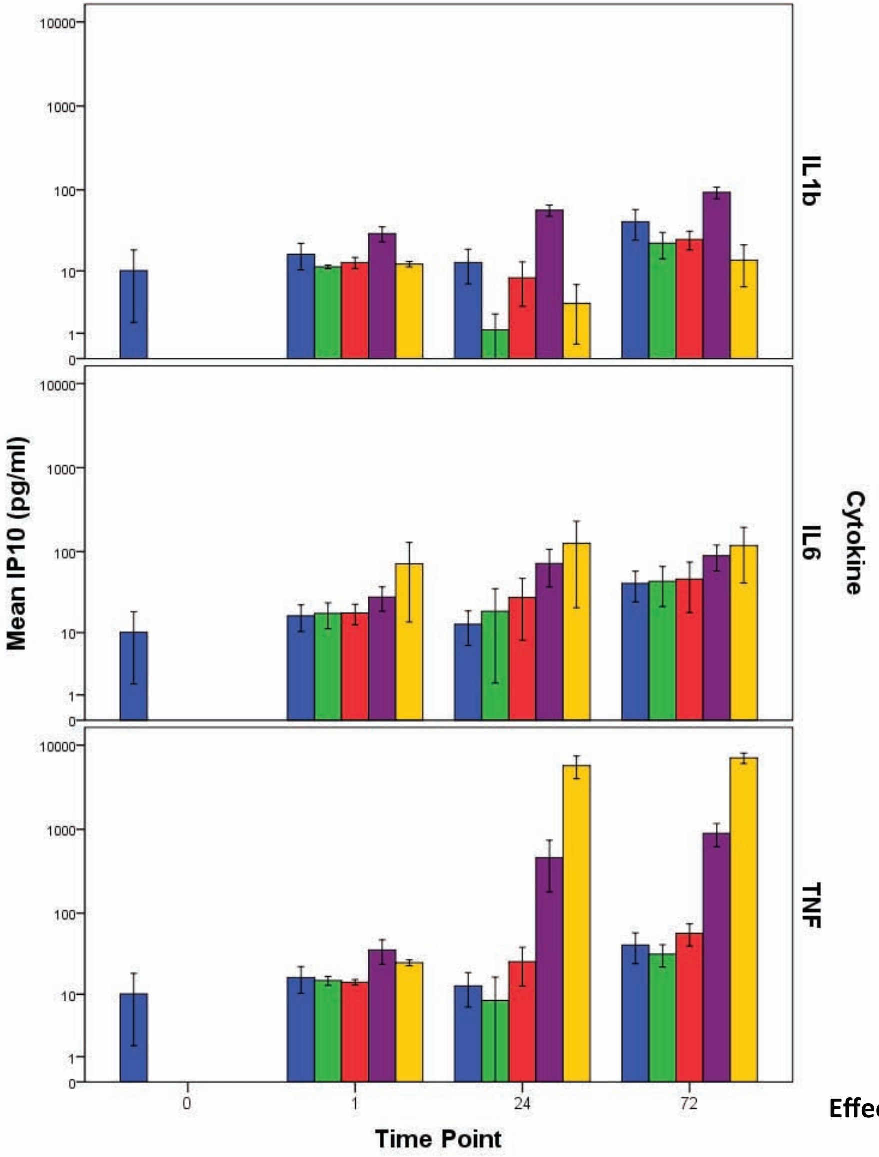

Note: Logarithmic scale to incorporate large variation in IP10 concentration

Two Way Mixed Anova

[illegible]

Post Hoc Tests

[b] within well contrast with most stringent p-value (Linear vs Quadratic vs Cubic

IL1b

Effect	ANOVA significance
Time[a]	<0.0001
Time*Concentration[a]	0.003
Concentration	0.004

Bonferroni: Differences Between Concentrations

Concentration	1	2	3	4
0	1	1	0.062	1
1		1	0.01	1
2			0.017	1
3				0.008

Polynomial Model: Differences in Time and Interaction of Time and Concentration

Effect	Best Model of Contrast[b]	Signficance[a]
Time	Linear	0.001
Time*Concentration	Linear	0.005

IL6

Effect	ANOVA significance
Time[a]	0.038
Time*Concentration[a]	0.607
Concentration	0.535

Polynomial Model: Differences in Time and Interaction of Time and Concentration

Effect	Best Model of Contrast[b]	Signficance[a]
Time	Linear	0.021

TNF

Effect	ANOVA significance
Time[a]	<0.0001
Time*Concentration[a]	<0.0001
Concentration	<0.0001

Bonferroni: Differences Between Concentrations

Concentration	1	2	3	4
0	1	1	1	<0.0001
1		1	1	<0.0001
2			1	<0.0001
3				<0.0001

Polynomial Model: Differences in Time and Interaction of Time and Concentration

Effect	Best Model of Contrast[b]	Signficance[a]
Time	Linear	<0.0001
Time*Concentration	Linear	<0.0001

Effect of Added Cytokine: Multivariate ANOVA

Time Point	1 Hour	24 Hours	72 Hours
Significance	<0.0001	0.036	0.011

Post Hoc Tests

IP10 1h	IL1β	IL6	TNF
None	1	1	1
IL1β		.946	1
IL6			1

IP10 24h	IL1β	IL6	TNF
None	1	1	0.847
IL1β		1	0.140
IL6			.163

IP10 72h	IL1β	IL6	TNF
None	1	1	0.564
IL1β		1	0.06
IL6			0.068

MCP-1

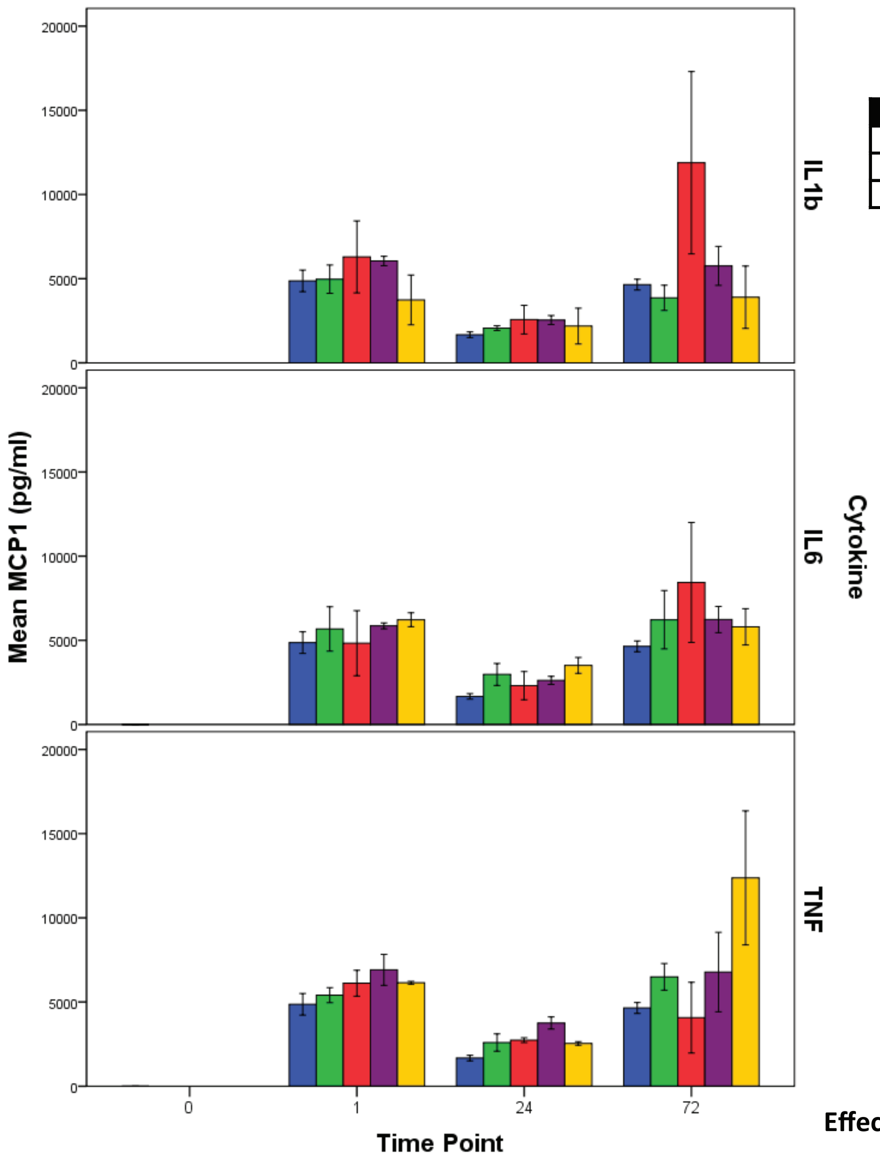

Two Way Mixed Anova

[a]Greenhouse-Geisser

Post Hoc Tests

[b]within well contrast with most stringent p-value (Linear vs Quadratic vs Cubic

Effect	ANOVA significance
Time[a]	<0.0001
Time*Concentration[a]	0.218
Concentration	0.318

Polynomial Model: Differences in Time and Interaction of Time and Concentration

Effect	Best Model of Contrast[b]	Signficance[a]
Time	Cubic	<0.0001

Effect	ANOVA significance
Time[a]	<0.0001
Time*Concentration[a]	0.599
Concentration	0.85

Polynomial Model: Differences in Time and Interaction of Time and Concentration

Effect	Best Model of Contrast[b]	Signficance[a]
Time	Linear/Cubic	<0.0001

Effect	ANOVA significance
Time[a]	<0.0001
Time*Concentration[a]	0.164
Concentration	0.128

Polynomial Model: Differences in Time and Interaction of Time and Concentration

Effect	Best Model of Contrast[b]	Signficance[a]
Time	Linear/Cubic	<0.0001

Effect of Added Cytokine: Multivariate ANOVA

Time Point	1 Hour	24 Hours	72 Hours
Significance	<0.0001	<0.0001	<0.0001

Post Hoc Tests

MCP1 1h	IL1β	IL6	TNF
None	1	1	1
IL1β		1	1
IL6			1

MCP1 24h	IL1β	IL6	TNF
None	1	0.308	0.257
IL1β		1	0.822
IL6			1

MCP1 72h	IL1β	IL6	TNF
None	1	1	1
IL1β		1	1
IL6			1

MCP-3

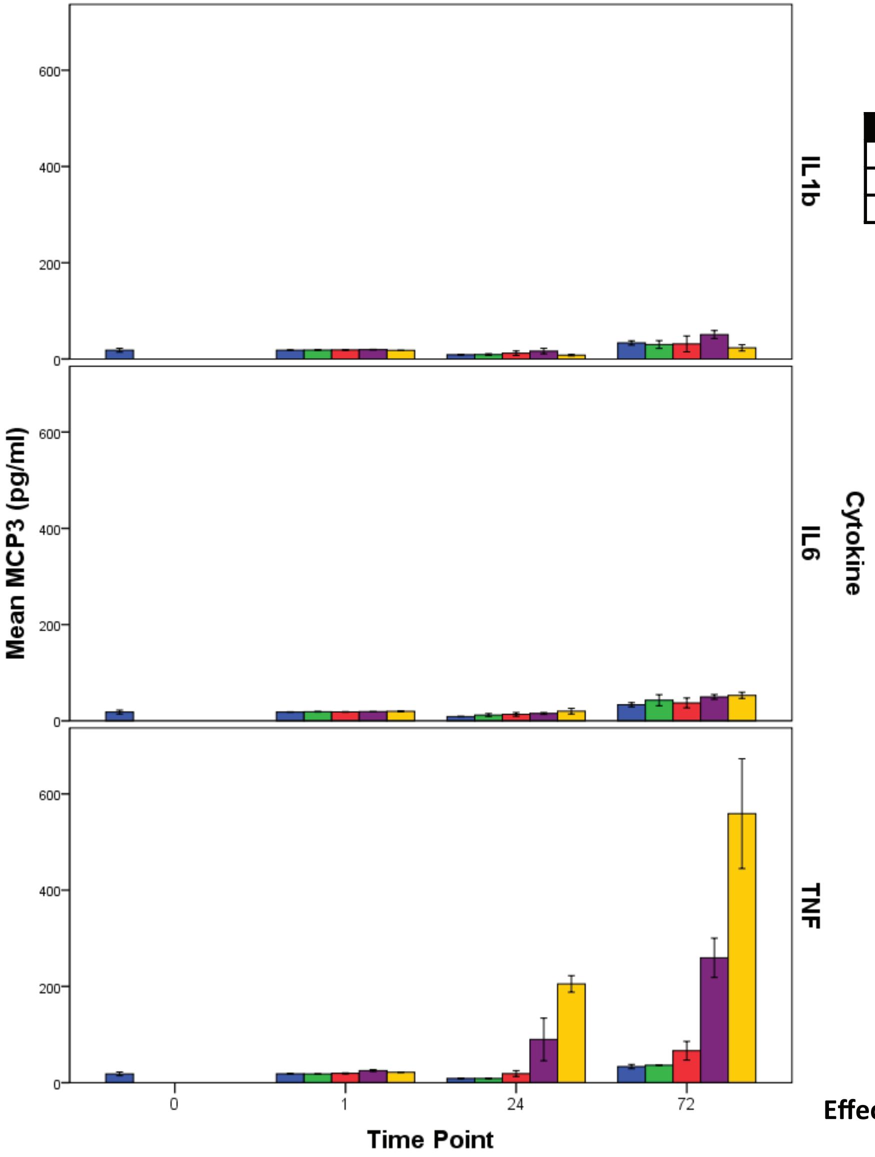

Two Way Mixed Anova

[a]Greenhouse-Geisser

Post Hoc Tests

[b]within well contrast with most stringent p-value (Linear vs Quadratic vs Cubic

IL1b

Effect	ANOVA significance
Time[a]	<0.0001
Time*Concentration[a]	0.512
Concentration	0.429

Polynomial Model: Differences in Time and Interaction of Time and Concentration

Effect	Best Model of Contrast[b]	Signficance[a]
Time	Quadratic/Cubic	<0.0001

IL6

Effect	ANOVA significance
Time[a]	<0.0001
Time*Concentration[a]	0.639
Concentration	0.348

Polynomial Model: Differences in Time and Interaction of Time and Concentration

Effect	Best Model of Contrast[b]	Signficance[a]
Time	Quadratic/Cubic	<0.0001

TNF

Effect	ANOVA significance
Time[a]	<0.0001
Time*Concentration[a]	<0.0001
Concentration	<0.0001

Bonferroni: Differences Between Concentrations

Concentration	1	2	3	4
0	1	1	0.051	<0.0001
1		1	0.053	<0.0001
2			0.118	<0.0001
3				0.009

Polynomial Model: Differences in Time and Interaction of Time and Concentration

Effect	Best Model of Contrast[b]	Signficance[a]
Time	Linear	<0.0001
Time*Concentration	Linear	<0.0001

Effect of Added Cytokine: Multivariate ANOVA

Time Point	1 Hour	24 Hours	72 Hours
Significance	<0.0001	<0.0001	<0.0001

Post Hoc Tests

MCP3 1h	IL1β	IL6	TNF
None	1	1	0.325
IL1β		1	0.073
IL6			0.169

MCP3 24h	IL1β	IL6	TNF
None	1	1	0.201
IL1β		1	0.011
IL6			0.018

MCP3 72h	IL1β	IL6	TNF
None	1	1	0.167
IL1β		1	0.006
IL6			0.01

MDC

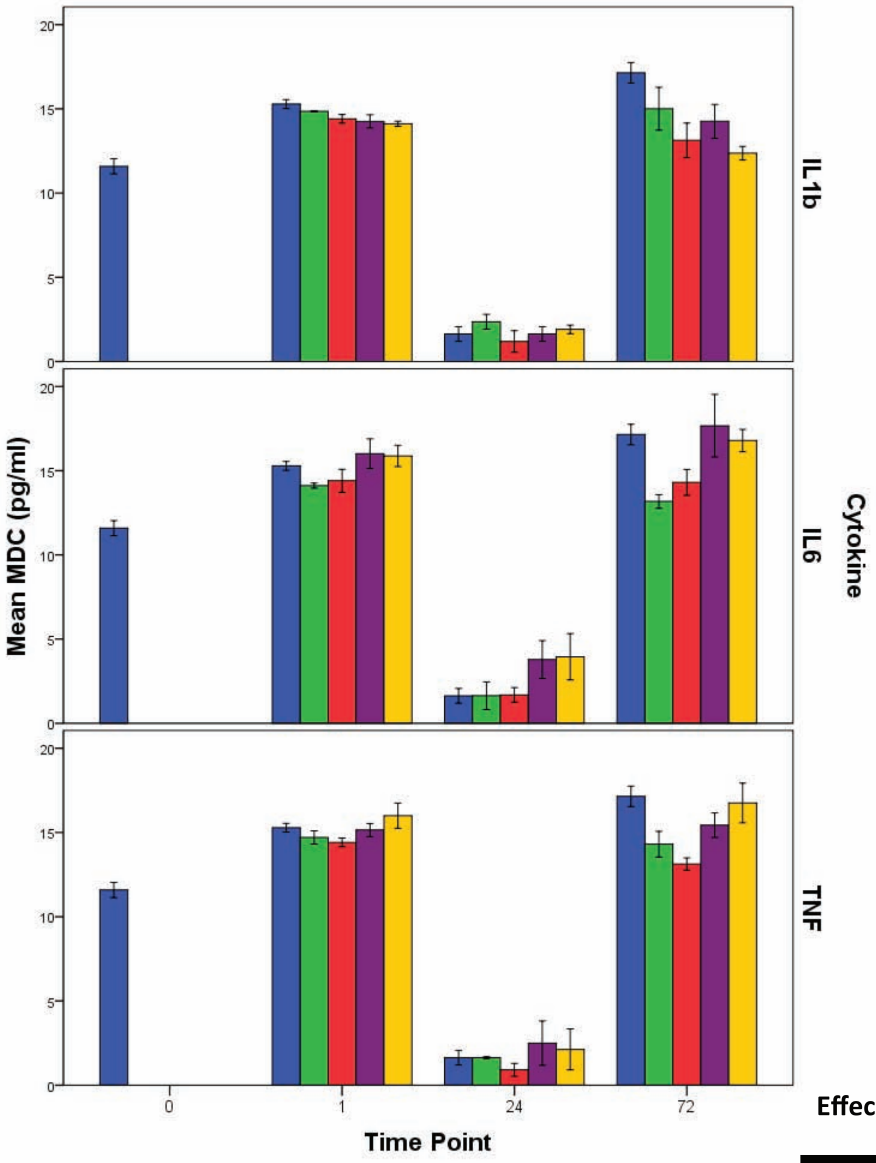

Two Way Mixed Anova

a Greenhouse-Geisser

Effect	ANOVA significance
Time[a]	<0.0001
Time*Concentration[a]	0.019
Concentration	0.102

Effect	ANOVA significance
Time[a]	<0.0001
Time*Concentration[a]	0.131
Concentration	0.054

Effect	ANOVA significance
Time[a]	<0.0001
Time*Concentration[a]	0.132
Concentration	0.110

Post Hoc Tests

b within well contrast with most stringent p-value (Linear vs Quadratic vs Cubic

Polynomial Model: Differences in Time and Interaction of Time and Concentration

Effect	Best Model of Contrast[b]	Signficance[a]
Time	Quadratic/Cubic	<0.0001
Time*Concentration	Cubic	0.006

Polynomial Model: Differences in Time and Interaction of Time and Concentration

Effect	Best Model of Contrast[b]	Signficance[a]
Time	Quadratic/Linear	<0.0001

Polynomial Model: Differences in Time and Interaction of Time and Concentration

Effect	Best Model of Contrast[b]	Signficance[a]
Time	Quadratic/Cubic	<0.0001

Effect of Added Cytokine: Multivariate ANOVA

Time Point	1 Hour	24 Hours	72 Hours
Significance	<0.0001	<0.0001	<0.0001

Post Hoc Tests

MDC 1h	IL1β	IL6	TNF
None	0.959	1	1
IL1β		0.492	0.585
IL6			1

MDC 24h	IL1β	IL6	TNF
None	1	1	1
IL1β		0.586	1
IL6			0.619

MDC 72h	IL1β	IL6	TNF
None	0.072	1	0.568
IL1β		0.219	0.896
IL6			1

MIP1a

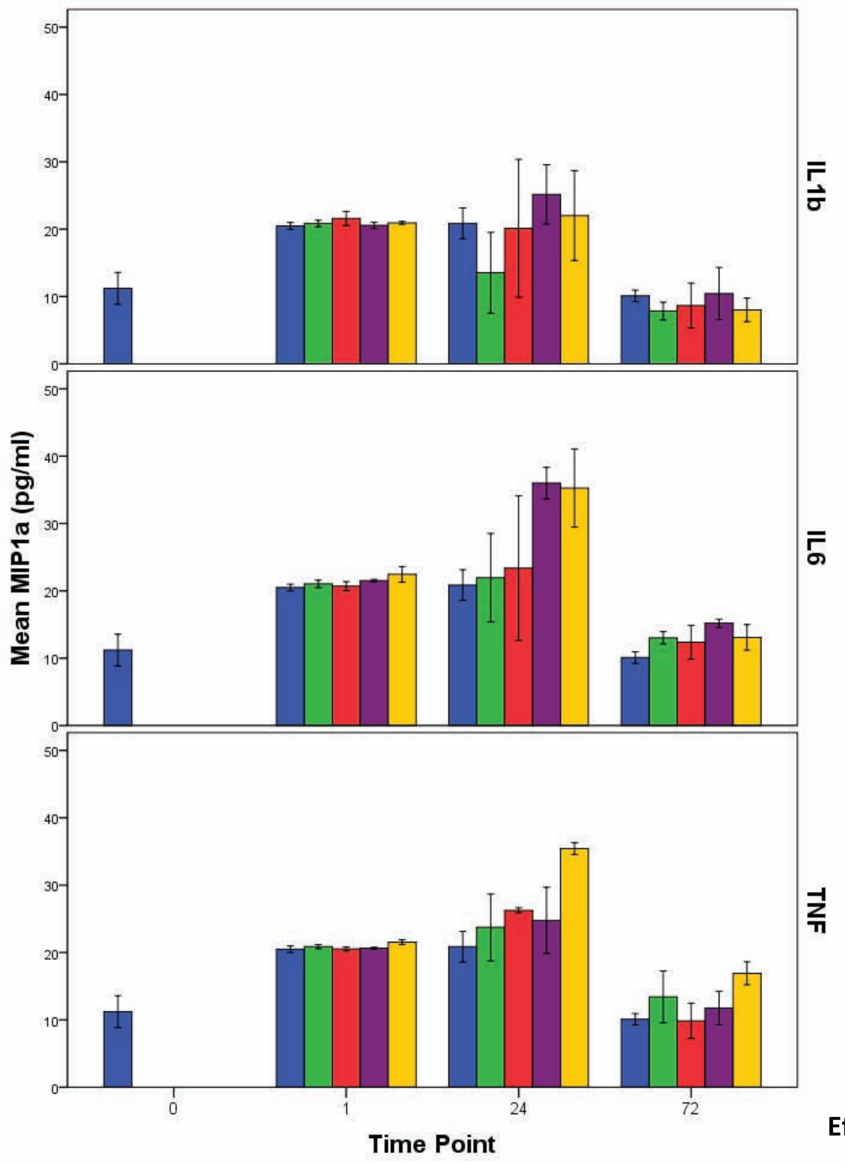

Two Way Mixed Anova

a Greenhouse-Geisser

Effect	ANOVA significance
Time[a]	<0.0001
Time*Concentration[a]	0.823
Concentration	0.897

Effect	ANOVA significance
Time[a]	<0.0001
Time*Concentration[a]	0.430
Concentration	0.236

Effect	ANOVA significance
Time[a]	<0.0001
Time*Concentration[a]	0.349
Concentration	0.053

Post Hoc Tests

b within well contrast with most stringent p-value (Linear vs Quadratic vs Cubic

Polynomial Model: Differences in Time and Interaction of Time and Concentration

Effect	Best Model of Contrast[b]	Signficance[a]
Time	Quadratic	<0.0001

Polynomial Model: Differences in Time and Interaction of Time and Concentration

Effect	Best Model of Contrast[b]	Signficance[a]
Time	Quadratic	<0.0001

Polynomial Model: Differences in Time and Interaction of Time and Concentration

Effect	Best Model of Contrast[b]	Signficance[a]
Time	Quadratic	<0.0001

Effect of Added Cytokine: Multivariate ANOVA

Time Point	1 Hour	24 Hours	72 Hours
Significance	<0.0001	<0.0001	<0.0001

Post Hoc Tests

MIP1a 1h	IL1β	IL6	TNF
None	1	0.913	1
IL1β		1	1
IL6			1

MIP1a 24h	IL1β	IL6	TNF
None	1	1	1
IL1β		0.247	0.541
IL6			1

MIP1a 72h	IL1β	IL6	TNF
None	1	1	1
IL1β		0.039	0.076
IL6			1

MIP1b

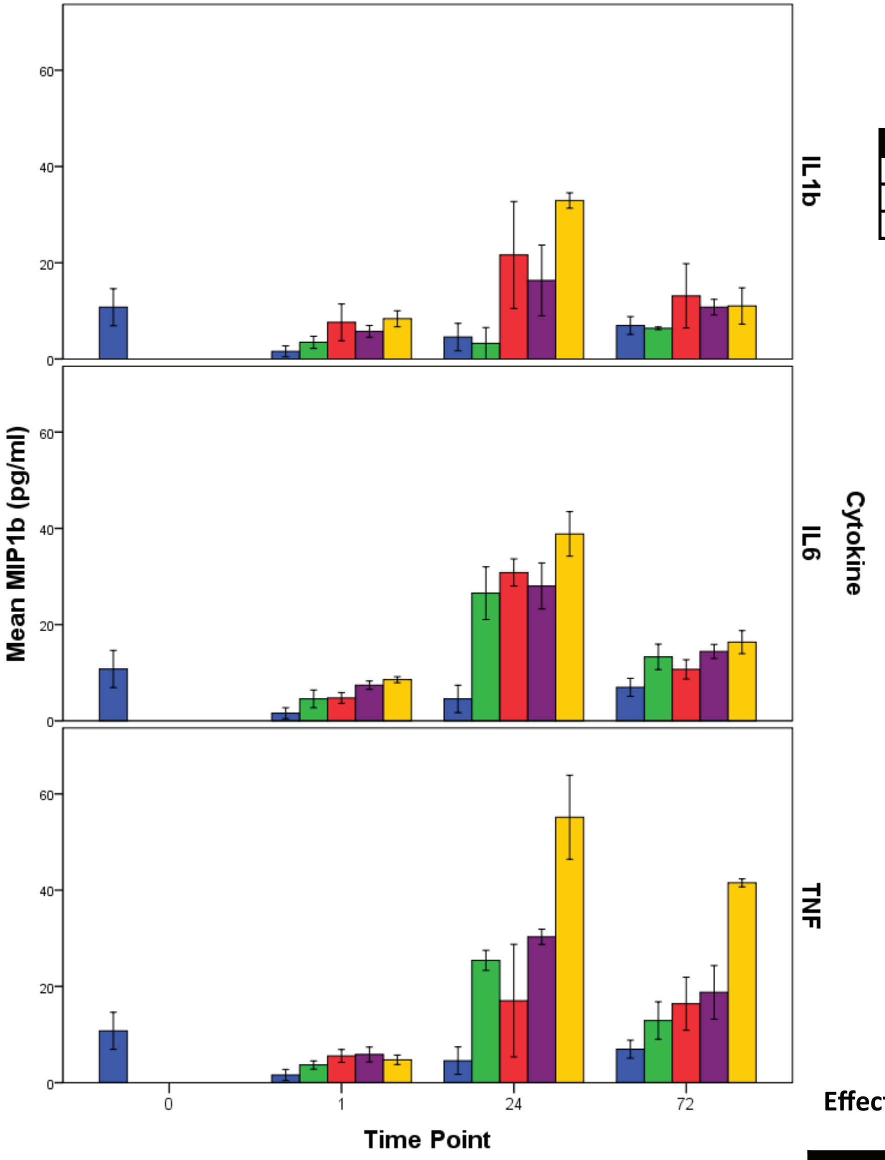

Two Way Mixed Anova

[a]Greenhouse-Geisser

Effect	ANOVA significance
Time[a]	0.004
Time*Concentration[a]	0.045
Concentration	0.146

Effect	ANOVA significance
Time[a]	<0.0001
Time*Concentration[a]	0.015
Concentration	0.002

Effect	ANOVA significance
Time[a]	<0.0001
Time*Concentration[a]	0.001
Concentration	0.004

Post Hoc Tests

[b]within well contrast with most stringent p-value (Linear vs Quadratic vs Cubic

Polynomial Model: Differences in Time and Interaction of Time and Concentration

Effect	Best Model of Contrast[b]	Signficance[a]
Time	Cubic	0.001
Time*Concentration	Quadratic	0.019

Bonferroni: Differences Between Concentrations

Concentration	1	2	3	4
0	0.051	0.036	0.018	0.002
1		1	1	0.515
2			1	0.738
3				1

Polynomial Model: Differences in Time and Interaction of Time and Concentration

Effect	Best Model of Contrast[b]	Signficance[a]
Time	Cubic	<0.0001
Time*Concentration	Quadratic	0.01

Bonferroni: Differences Between Concentrations

Concentration	1	2	3	4
0	1	1	0.279	0.003
1		1	1	0.044
2			1	0.033
3				0.171

Polynomial Model: Differences in Time and Interaction of Time and Concentratio

Effect	Best Model of Contrast[b]	Signficance[a]
Time	Linear/Cubic	<0.0001
Time*Concentration	Linear	0.001

Effect of Added Cytokine: Multivariate ANOVA

Time Point	1 Hour	24 Hours	72 Hours
Significance	<0.0001	<0.0001	<0.0001

Post Hoc Tests

MIP1b 1h	IL1β	IL6	TNF
None	0.093	0.092	0.468
IL1β		1	1
IL6			1

MIP1b 24h	IL1β	IL6	TNF
None	0.819	0.04	0.03
IL1β		0.227	0.159
IL6			1

MIP1b 72h	IL1β	IL6	TNF
None	1	1	0.053
IL1β		1	0.009
IL6			0.110

PDGF AA

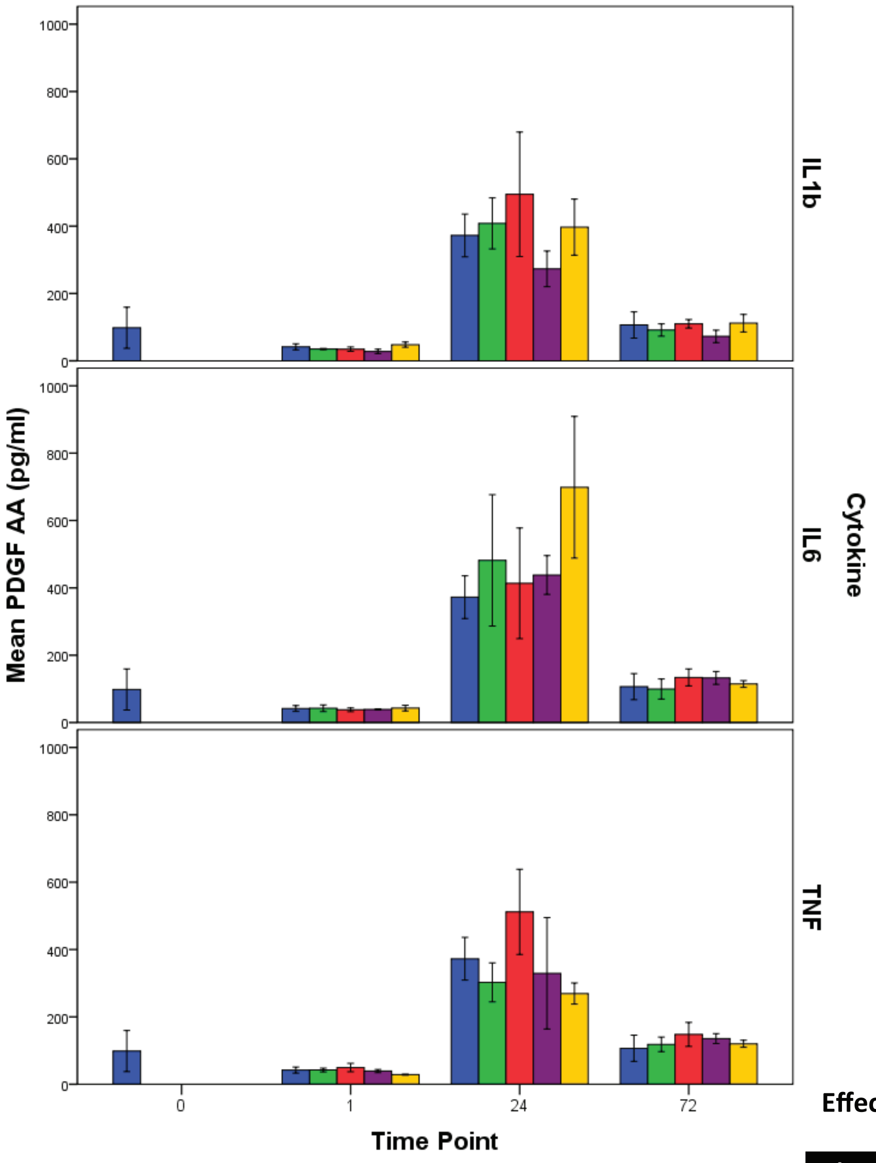

Two Way Mixed Anova
aGreenhouse-Geisser

Effect	ANOVA significance
Time[a]	<0.0001
Time*Concentration[a]	0.864
Concentration	0.762

Effect	ANOVA significance
Time[a]	<0.0001
Time*Concentration[a]	0.582
Concentration	0.842

Effect	ANOVA significance
Time[a]	<0.0001
Time*Concentration[a]	0.690
Concentration	0.683

Post Hoc Tests
bwithin well contrast with most stringent p-value (Linear vs Quadratic vs Cubic

Polynomial Model: Differences in Time and Interaction of Time and Concentration

Effect	Best Model of Contrast[b]	Signficance[a]
Time	Cubic	<0.0001

Polynomial Model: Differences in Time and Interaction of Time and Concentration

Effect	Best Model of Contrast[b]	Signficance[a]
Time	Linear/Quadratic/Cubic	<0.0001

Polynomial Model: Differences in Time and Interaction of Time and Concentration

Effect	Best Model of Contrast[b]	Signficance[a]
Time	Cubic	<0.0001

Effect of Added Cytokine: Multivariate ANOVA

Time Point	1 Hour	24 Hours	72 Hours
Significance	<0.0001	<0.0001	<0.0001

Post Hoc Tests

PDGF AA 1h	IL1β	IL6	TNF
None	1	1	1
IL1β		1	1
IL6			1

PDGF AA 24h	IL1β	IL6	TNF
None	1	1	1
IL1β		1	1
IL6			0.520

PDGF AA 72h	IL1β	IL6	TNF
None	1	1	1
IL1β		0.808	0.207
IL6			1

PDGF AB BB

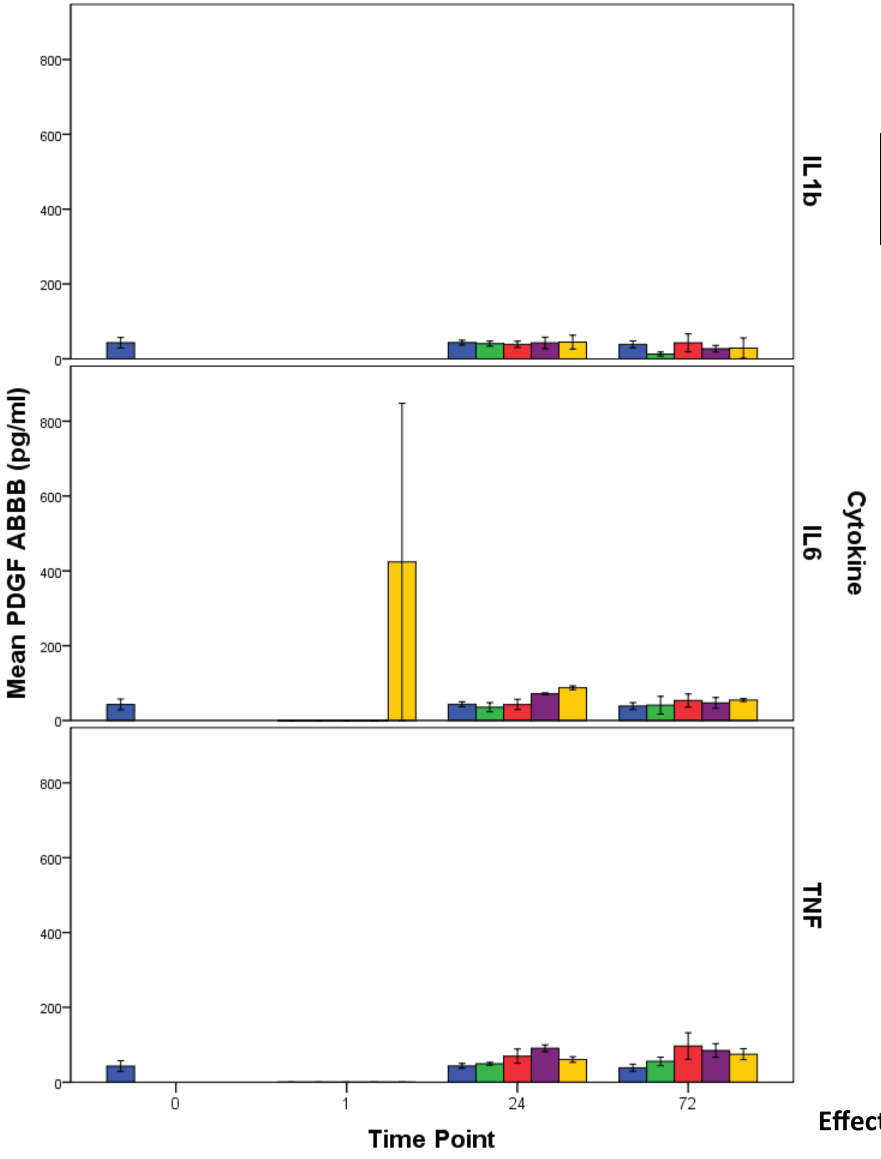

Two Way Mixed Anova

aGreenhouse-Geisser

Post Hoc Tests

bwithin well contrast with most stringent p-value (Linear vs Quadratic vs Cubic

IL1b

Effect	ANOVA significance
Time[a]	<0.0001
Time*Concentration[a]	0.960
Concentration	0.973

Polynomial Model: Differences in Time and Interaction of Time and Concentration

Effect	Best Model of Contrast[b]	Signficance[a]
Time	Cubic	<0.0001

IL6

Effect	ANOVA significance
Time[a]	0.671
Time*Concentration[a]	0.484
Concentration	0.370

TNF

Effect	ANOVA significance
Time[a]	<0.0001
Time*Concentration[a]	0.409
Concentration	0.290

Polynomial Model: Differences in Time and Interaction of Time and Concentration

Effect	Best Model of Contrast[b]	Signficance[a]
Time	Cubic	<0.0001

Effect of Added Cytokine: Multivariate ANOVA

Time Point	1 Hour	24 Hours	72 Hours
Significance	0.536	<0.0001	<0.0001

Post Hoc Tests

PDGF AB 24h	IL1β	IL6	TNF
None	1	1	0.651
IL1β		0.397	0.051
IL6			1

PDGF AB 72h	IL1β	IL6	TNF
None	1	1	0.308
IL1β		0.569	0.002
IL6			0.146

RANTES

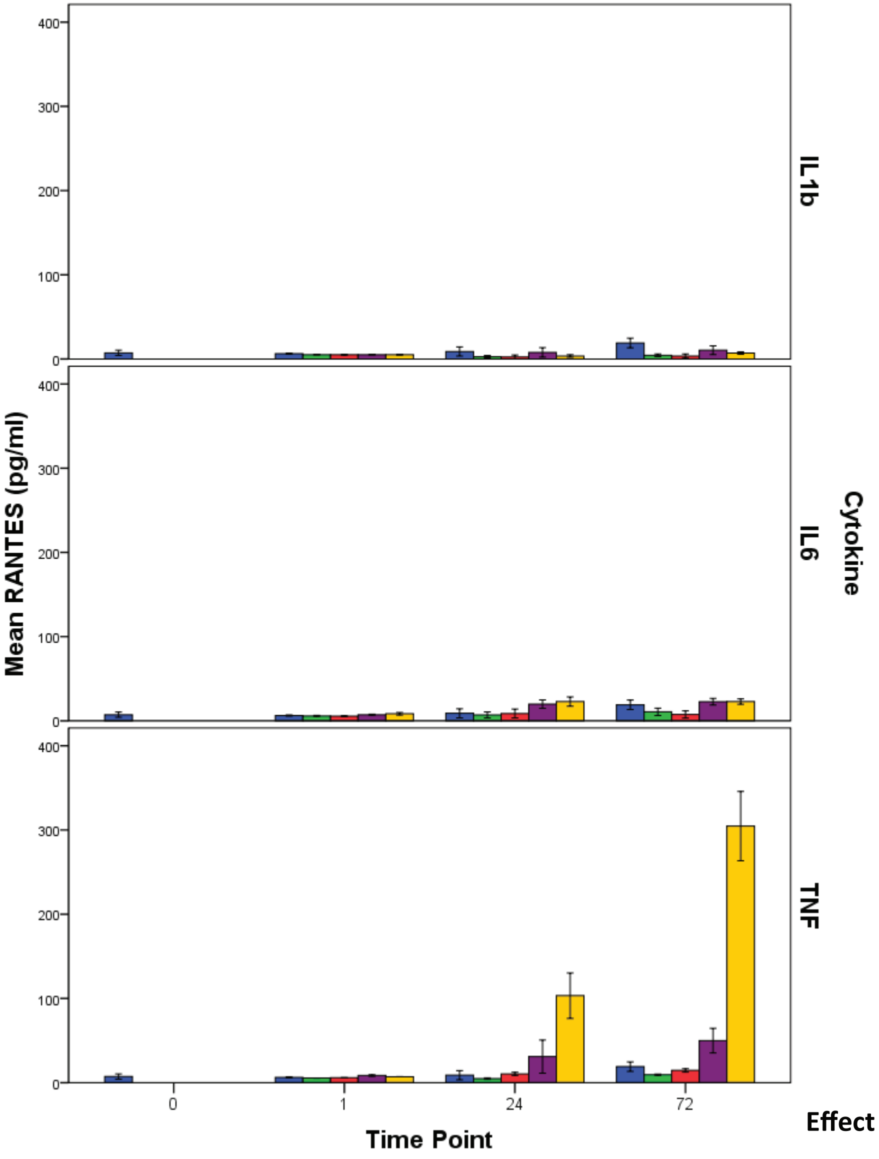

Two Way Mixed Anova
a Greenhouse-Geisser

Post Hoc Tests
b within well contrast with most stringent p-value (Linear vs Quadratic vs Cubic

IL1b

Effect	ANOVA significance
Time[a]	0.138
Time*Concentration[a]	0.321
Concentration	0.334

IL6

Effect	ANOVA significance
Time[a]	0.003
Time*Concentration[a]	0.240
Concentration	0.117

Polynomial Model: Differences in Time and Interaction of Time and Concentration

Effect	Best Model of Contrast[b]	Signficance[a]
Time	Cubic	0.012

TNF

Effect	ANOVA significance
Time[a]	<0.0001
Time*Concentration[a]	<0.0001
Concentration	<0.0001

Bonferroni: Differences Between Concentrations

Concentration	1	2	3	4
0	1	1	1	<0.0001
1		1	1	<0.0001
2			1	<0.0001
3				<0.0001

Polynomial Model: Differences in Time and Interaction of Time and Concentration

Effect	Best Model of Contrast[b]	Signficance[a]
Time	Linear/Quadratic	<0.0001
Time*Concentration	Linear/Quadratic	<0.0001

Effect of Added Cytokine: Multivariate ANOVA

Time Point	1 Hour	24 Hours	72 Hours
Significance	<0.0001	0.001	0.002

Post Hoc Tests

RANTES 1h	IL1β	IL6	TNF
None	1	1	1
IL1β		0.039	0.047
IL6			1

RANTES 24h	IL1β	IL6	TNF
None	1	1	0.722
IL1β		1	0.035
IL6			0.312

RANTES 72h	IL1β	IL6	TNF
None	1	1	0.739
IL1β		1	0.037
IL6			0.081

sCD40L

Two Way Mixed Anova
a Greenhouse-Geisser

Post Hoc Tests
b within well contrast with most stringent p-value (Linear vs Quadratic vs Cubic

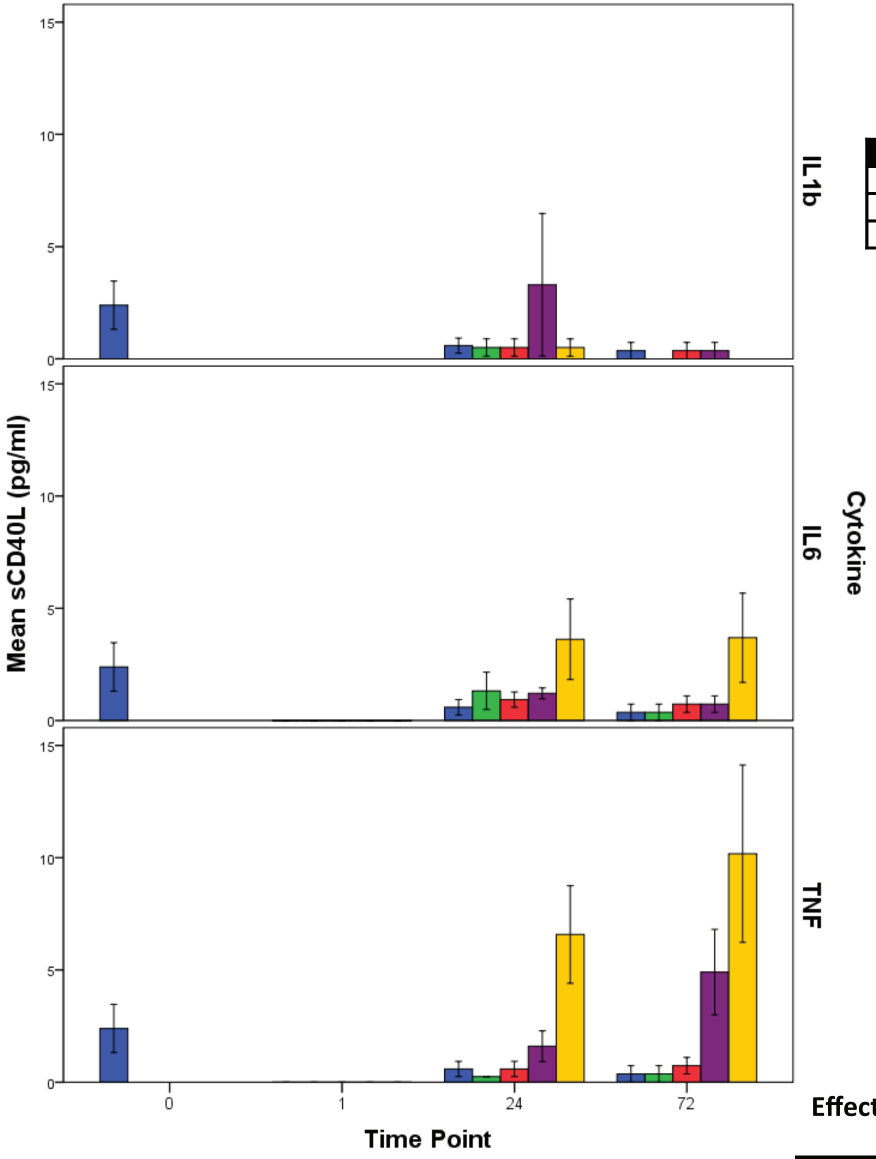

Effect	ANOVA significance
Time[a]	0.009
Time*Concentration[a]	0.880
Concentration	0.630

Polynomial Model: Differences in Time and Interaction of Time and Concentration

Effect	Best Model of Contrast[b]	Signficance[a]
Time	Linear	0.011

Effect	ANOVA significance
Time[a]	0.011
Time*Concentration[a]	0.687
Concentration	0.024

Bonferroni: Differences Between Concentrations

Concentration	1	2	3	4
0	1	1	1	0.042
1		1	1	0.085
2			1	0.083
3				0.109

Polynomial Model: Differences in Time and Interaction of Time and Concentration

Effect	Best Model of Contrast[b]	Signficance[a]
Time	Cubic	0.004

Effect	ANOVA significance
Time[a]	0.014
Time*Concentration[a]	0.045
Concentration	0.001

Bonferroni: Differences Between Concentrations

Concentration	1	2	3	4
0	1	1	0.737	0.002
1		1	0.601	0.002
2			0.918	0.002
3				0.042

Polynomial Model: Differences in Time and Interaction of Time and Concentration

Effect	Best Model of Contrast[b]	Signficance[a]
Time	Quadratic	0.019
Time*Concentration	Linear	0.013

Effect of Added Cytokine: Multivariate ANOVA

Time Point	24 Hours	72 Hours
Significance	0.005	0.002

Post Hoc Tests

sCD40L 24h	IL1β	IL6	TNF
None	1	1	1
IL1β		1	1
IL6			1

sCD40L 72h	IL1β	IL6	TNF
None	1	1	0.495
IL1β		1	0.032
IL6			0.289

sIL2Ra

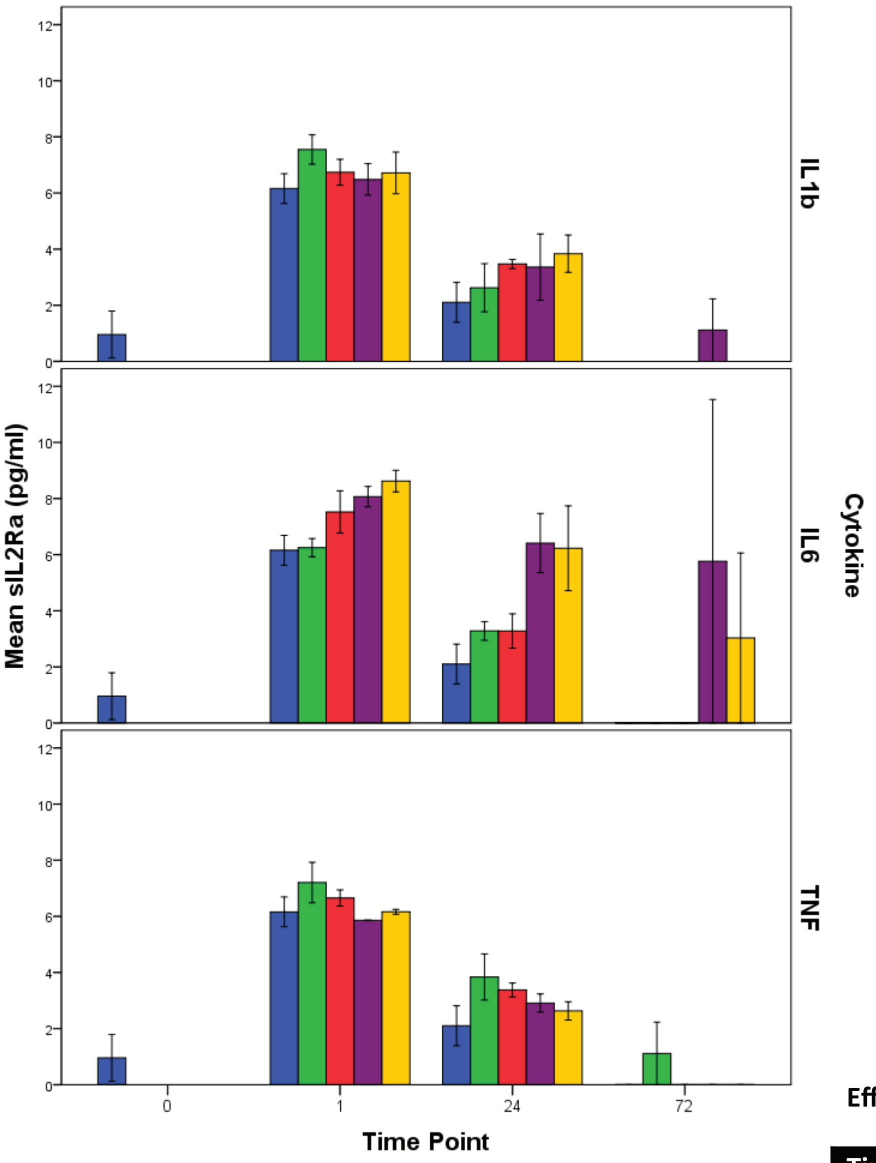

Two Way Mixed Anova

a Greenhouse-Geisser

Post Hoc Tests

b within well contrast with most stringent p-value (Linear vs Quadratic vs Cubic

IL1b

Effect	ANOVA significance
Time[a]	<0.0001
Time*Concentration[a]	0.788
Concentration	0.797

Polynomial Model: Differences in Time and Interaction of Time and Concentration

Effect	Best Model of Contrast[b]	Signficance[a]
Time	Linear/Quadratic/Cubic	<0.0001

IL6

Effect	ANOVA significance
Time[a]	0.001
Time*Concentration[a]	0.748
Concentration	0.037

Bonferroni: Differences Between Concentrations

Concentration	1	2	3	4
0	1	1	0.109	0.313
1		1	0.192	0.549
2			0.339	0.952
3				1

Polynomial Model: Differences in Time and Interaction of Time and Concentration

Effect	Best Model of Contrast[b]	Signficance[a]
Time	Quadratic/Cubic	0.001

TNF

Effect	ANOVA significance
Time[a]	<0.0001
Time*Concentration[a]	0.893
Concentration	0.342

Polynomial Model: Differences in Time and Interaction of Time and Concentration

Effect	Best Model of Contrast[b]	Signficance[a]
Time	Quadratic/Cubic	<0.0001

Effect of Added Cytokine: Multivariate ANOVA

Time Point	1 Hour	24 Hours	72 Hours
Significance	<0.0001	<0.0001	0.217

Post Hoc Tests

sIL2Ra 1h	IL1β	IL6	TNF
None	1	0.164	1
IL1β		0.432	1
IL6			0.042

sIL2Ra 24h	IL1β	IL6	TNF
None	1	0.055	1
IL1β		0.135	1
IL6			0.08

sIL2Ra 72h	IL1β	IL6	TNF
None	1	1	1
IL1β		0.856	1
IL6			0.856

TNF

Two Way Mixed Anova
aGreenhouse-Geisser

Post Hoc Tests
bwithin well contrast with most stringent p-value (Linear vs Quadratic vs Cubic

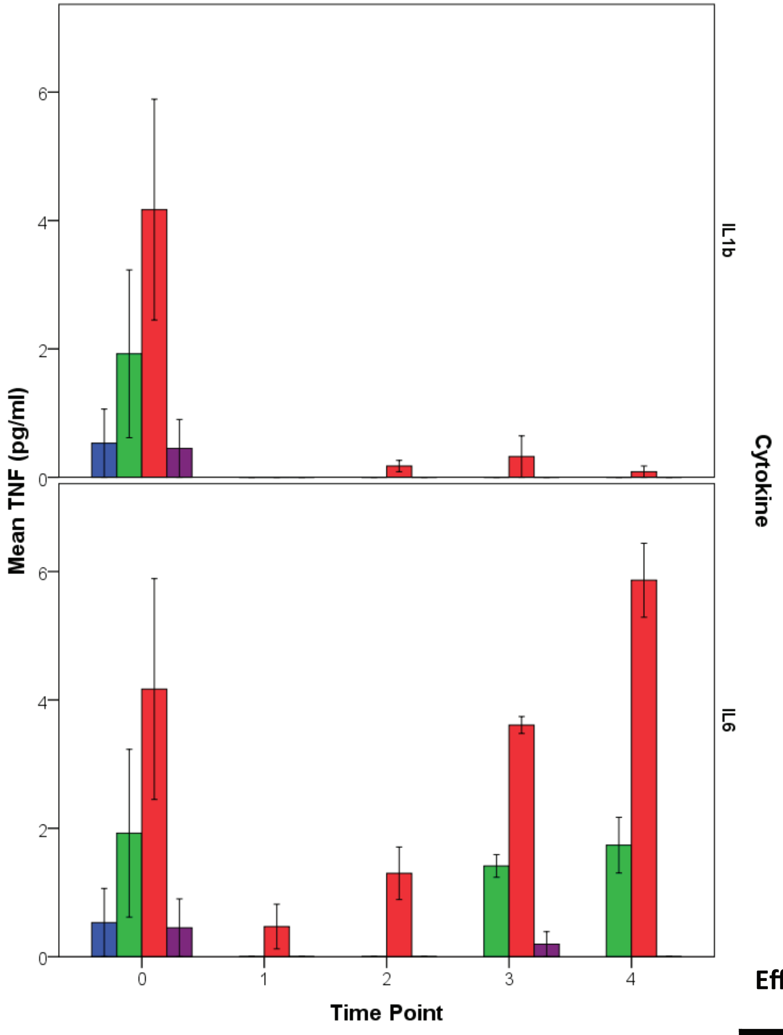

Effect	ANOVA significance
Time[a]	0.052
Time*Concentration[a]	0.010
Concentration	0.076

[a]Greenhouse-Geisser

Polynomial Model: Differences in Time and Interaction of Time and Concentration

Effect	Best Model of Contrast[b]	Signficance[a]
Time	Quadratic	0.006

[b]Within-well contrast with most stringent p-value(Linear vs Quadratic vs Cubic)

Effect	ANOVA significance
Time[a]	<0.0001
Time*Concentration[a]	0.002
Concentration	0.059

[a]Greenhouse-Geisser

Polynomial Model: Differences in Time and Interaction of Time and Concentration

Effect	Best Model of Contrast[b]	Signficance[a]
Time	Quadratic/Cubic	<0.0001
Time*Concentration	Quadratic	0.003

[b]Within-well contrast with most stringent p-value (Linear vs Quadratic vs Cubic)

Effect of Added Cytokine: Multivariate ANOVA

Time Point	1 Hour	24 Hours	72 Hours
Significance	0.001	<0.0001	0.043

Post Hoc Tests

Cytokine	IL1β	IL6
None	1	1
IL1β		1
IL6		

Cytokine	IL1β	IL6
None	1	1
IL1β		1
IL6		

Cytokine	IL1β	IL6
None	1	1
IL1β		1
IL6		

TNFb

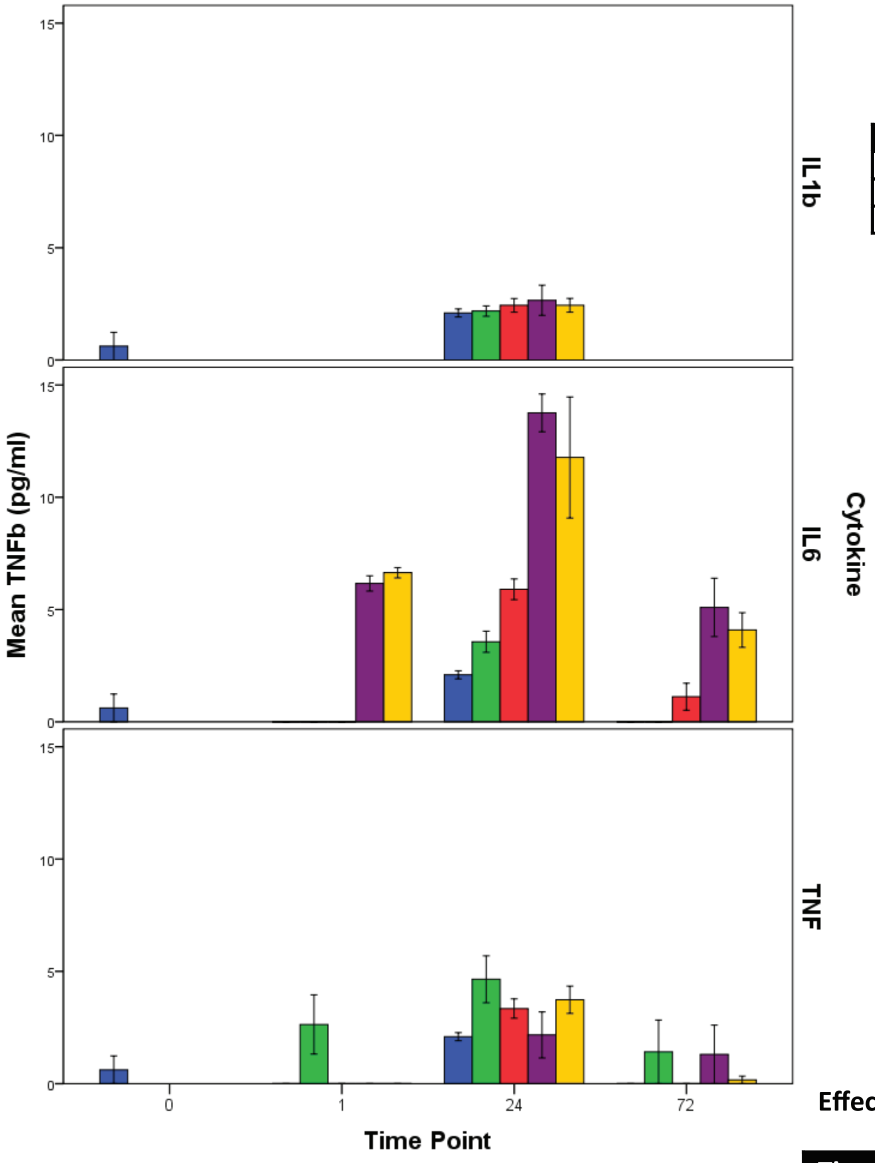

Two Way Mixed Anova

[a]Greenhouse-Geisser

Post Hoc Tests

[b]within well contrast with most stringent p-value (Linear vs Quadratic vs Cubic

IL1b

Effect	ANOVA significance
Time[a]	<0.0001
Time*Concentration[a]	0.996
Concentration	0.977

Polynomial Model: Differences in Time and Interaction of Time and Concentration

Effect	Best Model of Contrast[b]	Signficance[a]
Time	Quadratic/Cubic	<0.0001

IL6

Effect	ANOVA significance
Time[a]	<0.0001
Time*Concentration[a]	0.001
Concentration	<0.0001

Bonferroni: Differences Between Concentrations

Concentration	1	2	3	4
0	1	0.299	<0.0001	<0.0001
1		1	<0.0001	<0.0001
2			<0.0001	<0.0001
3				1

Polynomial Model: Differences in Time and Interaction of Time and Concentration

Effect	Best Model of Contrast[b]	Signficance[a]
Time	Linear/Quadratic/Cubic	<0.0001
Time*Concentration	Linear	<0.0001

TNF

Effect	ANOVA significance
Time[a]	<0.0001
Time*Concentration[a]	0.336
Concentration	0.173

Polynomial Model: Differences in Time and Interaction of Time and Concentration

Effect	Best Model of Contrast[b]	Signficance[a]
Time	Cubic	<0.0001

Effect of Added Cytokine: Multivariate ANOVA

Time Point	1 Hour	24 Hours	72 Hours
Significance[a]	<0.0001	<0.0001	<0.0001

Post Hoc Tests

TNFb 1h	IL1β	IL6	TNF
None	1	0.133	1
IL1β		0.003	1
IL6			0.029

TNFb 24h	IL1β	IL6	TNF
None	1	0.006	1
IL1β		<0.0001	1
IL6			<0.0001

TNFb 72h	IL1β	IL6	TNF
None	1	0.126	1
IL1β		0.003	1
IL6			0.056

VEGF

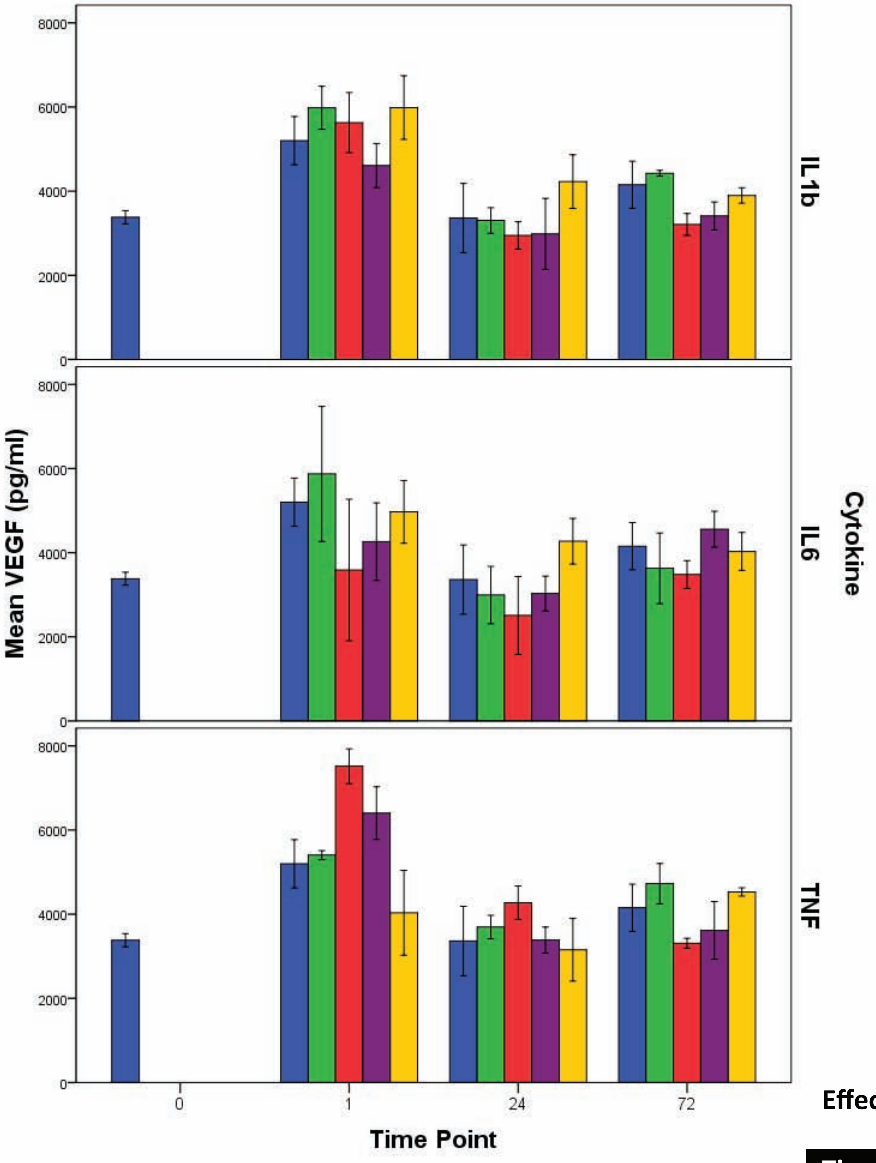

Two Way Mixed Anova

[a]Greenhouse-Geisser

Post Hoc Tests

[b]within well contrast with most stringent p-value (Linear vs Quadratic vs Cubic

IL1b

Effect	ANOVA significance
Time[a]	<0.0001
Time*Concentration[a]	0.508
Concentration	0.481

Polynomial Model: Differences in Time and Interaction of Time and Concentration

Effect	Best Model of Contrast[b]	Signficance[a]
Time	Cubic	<0.0001

IL6

Effect	ANOVA significance
Time[a]	0.010
Time*Concentration[a]	0.545
Concentration	0.792

Polynomial Model: Differences in Time and Interaction of Time and Concentration

Effect	Best Model of Contrast[b]	Signficance[a]
Time	Cubic	0.001

TNF

Effect	ANOVA significance
Time[a]	<0.0001
Time*Concentration[a]	0.007
Concentration	0.467

Polynomial Model: Differences in Time and Interaction of Time and Concentration

Effect	Best Model of Contrast[b]	Signficance[a]
Time	Cubic	<0.0001

Effect of Added Cytokine: Multivariate ANOVA

Time Point	1 Hour	24 Hours	72 Hours
Significance	<0.0001	<0.0001	<0.0001

Post Hoc Tests

Cytokine	IL1β	IL6	TNF
None	1	1	1
IL1β		1	1
IL6			0.543

Cytokine	IL1β	IL6	TNF
None	1	1	1
IL1β		1	1
IL6			1

Cytokine	IL1β	IL6	TNF
None	1	1	1
IL1β		1	1
IL6			1
